# Novel insights for a nonlinear deterministic-stochastic class of fractional-order Lassa fever model with varying kernels

**DOI:** 10.1038/s41598-023-42106-0

**Published:** 2023-09-15

**Authors:** Saima Rashid, Shazia Karim, Ali Akgül, Abdul Bariq, S. K. Elagan

**Affiliations:** 1 Department of Mathematics, Government College University, Faisalabad, 38000 Pakistan; 2 Department of Computer Science and Mathematics, Lebanese American University, Beirut, Lebanon; 3 Department of Basic Sciences, UET Lahore, FSD Campus, Lahore, 54800 Pakistan; 4 Department of Mathematics, Art and Science Faculty, Siirt University, 56100 Siirt, Turkey; 5 Department of Mathematics, Mathematics Research Center, Near East University, Near East Boulevard, 99138 Nicosia/Mersin 10, Turkey; 6 Department of Mathematics, Laghman University, Mehtarlam City, 2701 Laghman Afghanistan; 7 Department of Mathematics and Statistics, Taif University, Taif, Saudi Arabia

**Keywords:** Biophysics, Health care, Mathematics and computing

## Abstract

Lassa fever is a hemorrhagic virus infection that is usually spread by rodents. It is a fatal infection that is prevalent in certain West African countries. We created an analytical deterministic-stochastic framework for the epidemics of Lassa fever employing a collection of ordinary differential equations with nonlinear solutions to identify the influence of propagation processes on infected development in individuals and rodents, which include channels that are commonly overlooked, such as ecological emergent and aerosol pathways. The findings shed light on the role of both immediate and subsequent infectiousness via the power law, exponential decay and generalized Mittag-Leffler kernels. The scenario involves the presence of a steady state and an endemic equilibrium regardless of the fundamental reproduction number, $\Re _{0}<1$, making Lassa fever influence challenging and dependent on the severity of the initial sub-populations. Meanwhile, we demonstrate that the stochastic structure has an exclusive global positive solution via a positive starting point. The stochastic Lyapunov candidate approach is subsequently employed to determine sufficient requirements for the existence and uniqueness of an ergodic stationary distribution of non-negative stochastic simulation approaches. We acquire the particular configuration of the random perturbation associated with the model’s equilibrium $\Re _{0}^{s}<1$ according to identical environments as the presence of a stationary distribution. Ultimately, modeling techniques are used to verify the mathematical conclusions. Our fractional and stochastic findings exhibit that when all modes of transmission are included, the impact of Lassa fever disease increases. The majority of single dissemination pathways are less detrimental with fractional findings; however, when combined with additional spread pathways, they boost the Lassa fever stress.

## Introduction

Lassa fever , formerly known as Lassa hemorrhagic fever, is a deadly infectious species with serious consequences for the public’s health^1^. The Lassa fever is primarily circulated by rodents (a multi-mammate rat) and is prevalent in West African countries^1^. Lassa fever is an extremely infectious condition characterized by an elevated temperature (38 C$^{\circ }$) and the degeneration of internal organs (including the spleen)^2^. The condition has been named after Lassa, a municipality in Borno State, located in Nigeria’s northeastern region, where the initially identified Lassa fever case was discovered in 1969^2–4^.

Lassa fever ways of dissemination encompass rodent-to-rodent, rodent-to-human, human-to-human, human-to-rodent and human-to-environment^5–14^. In accordance with the World Health Organization^14^, approximately 80% of Lassa fever-affected individuals exhibit no clinical signs (i.e., are asymptomatic), and one in every five contaminated individuals has been determined to be in a severe inflammation scenario^13–15^.Lassa virus infection is the underlying cause of Lassa fever, and it has an elevated death rate, particularly among expectant mothers and individuals with pre-existing medical histories^4^. According to an investigation carried out by Richmond et al.^16^, Lassa virus could potentially be employed to deploy missiles featuring infectious bacteria or physical arsenals. The infection has impressive efficacy in its spread. One instance of Lassa fever becoming infected in an entire community may initiate a pandemic^17^. As a result of the socioeconomic and biological consequences of Lassa virus, more research on the virus is required to gain a better understanding of transmission mechanisms and control.

Undoubtedly, the Lassa virus’s host is a multi-mammate rodent (commonly referred to as Mastomys natalensis) that develops repeatedly and spreads extensively throughout the West Africa. Rodents from infected environments are seven times more likely to become infected compared with animals from controlled environments^16^. Yearly, roughly thirty thousand intriguing Lassa virus ailments occur, with 5500–15,000 casualties^3,5,13^. Despite this, there is currently no approved vaccine for Lassa fever. Nonetheless, it can be successfully alleviated by the antiviral drug, which is widely accessible and highly efficient if administered shortly after the start of the course of infection (i.e., throughout the six weeks of illness onset)^14^. Furthermore, as reported in^10^, Lassa fever implementation may necessitate medication for viruses, substance substitution, and bloodstream transplants. As a result, successful treatment for Lassa fever getting sick is unable to ensure permanent resistance to recurrence^10^.Certain elements may contribute to the prevalence (for example, human-to-human sickness, rodent-to-rodent illness, and ecological damage), whereas individuals (for example, therapy, ecological decontamination, and medical education campaigns) can lower the illness stress in a particular region.

**Figure 1:**
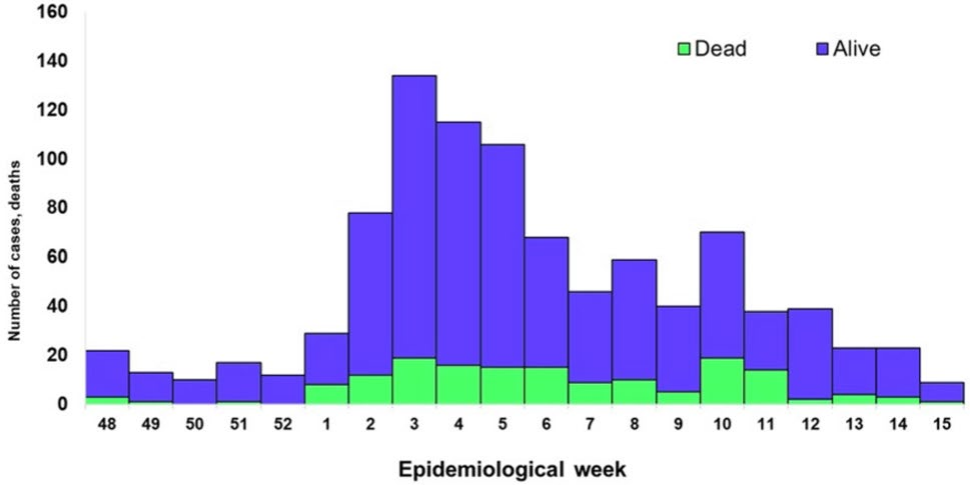
Confirmed Lassa fever cases in Nigeria epidemiological week 48, 2022 to week 15, 2023.

Because of its significant incidence and the possibility of dissemination, the World Health Organization decided to place Lassa fever on its model, identifying critical illnesses that require greater involvement via healthcare administrators and scientists to enable greater focus on mitigation and regulation strategies^18^. To the extent of our understanding, few research investigations have been conducted with the objective of shedding more insight into the prevalence and medical manifestations of Lassa fever. As a result, additional scientific backing and epidemiological inquiries on the evolution of the propagation of Lassa fever are required, particularly with regard to the effect of elements influencing the environment. The Lassa fever time series case data were obtained from the open website of the Nigeria Centre for Disease Control^19^ for the period of November 28, 2022, to April 13, 2023. All case data are laboratory confirmed by the Nigeria Centre for Disease Control situation report^19^. Figure 1 presents the number of Lassa fever laboratory cases confirmed weekly by states in Nigeria.

While searching for evidence, we discovered that multiple scholars have proposed finding algorithms that are capable of being utilized for obtaining fractional differential operators. The primary explanation for why this happens is that in practical application, obstacles prove manifestations of procedures that are analogous to the behaviours displayed by certain mathematical formulas. The discoveries of Hadamard, Caputo, Riez and Hilfer contribute to a fractional calculus that contains an index-law kernel. Because of Caputo’s subsequent improvements, which enabled the use of classical initial values, the resulting form has been used in a variety of scientific fields^20^. Prabhakar^21^ contemplated an alternative kernel via three settings as an outcome of the power-law and the generalized Mittag-Leffler function. Numerous investigators have felt drawn to this adaptation, and investigations on both concepts and their implementation were carried out^22–32^. Actually, both of the algorithms have distinct principles; e.g., the index-law kernel merely aids in the replication of procedures that demonstrate power-law actions, whereas the combination of the index-law and the generalized three-parameter kernel assists in the replication of procedures that indicate power-law behaviour. Mittag-Leffler encounters a sphere with potential as well^21,33^. Because the environment is convoluted, Caputo and Fabrizio^34^ proposed an innovative kernel: an unusual exponential kernel alongside Delta Dirac features. A differential operator that is well-noted currently since it has the capability to reproduce procedures after diminishing memory. In fact, the notion of the fractional derivative that works with a non-singular kernel was developed by this kernel, ushering in an entirely novel era in fractional calculus^35^. Several of the investigators observations of the kernel’s non-fractionality prompted the development of an additional kernel, the generalized Mittag-Leffler work, that had one setting. Atangana and Baleanu^35^ suggested this formulation, which signifies yet another expansion breakthrough in the field of fractional calculus. The fractional derivative techniques are being successfully implemented in a variety of research disciplines of research. Author^36^ presents the fundamental concepts of fractional differentiation, existence-uniqueness concepts and computational approaches to solving fractional differential equation. Nevertheless, whereas the crossover features of the Mittag-Leffler and the exponential kernel are widely identified as powerful mathematical approaches for illustrating practical problems, it is critical to recall that solely the core problems observing the crossover features of each of these approaches can be simulated according to multiple limitations, as in major difficulties, these two components are likely ineffective in confirming precisely at which the crossover happened.Atangana and Seda^37^ just introduced intriguing concepts referred to as piecewise differentiation and integration, of which a contemporary variant is defined as a piecewise within a specific time frame. The following is a previously developed mathematical instrument for facilitating multifaceted, significant challenges alongside convoluted cross-over practises. A new method of illustration will address an extensive variety of structural problems. But none of them observed random perturbation techniques in their inquiries, and just a handful of mathematical examinations via Lassa fever diseases of the model have been carried out. To the best of our understanding, no investigation has been performed to analyze the Lassa fever transmission pathways of the illnesses mentioned in a trustworthy and inexpensive manner. Rashid et al.^38^ presented the new numerical simulation for the fractional model of deathly Lassa hemorrhagic fever disease in pregnant women with optimal analysis. Atangana and Rashid^39^ contemplated the analysis of a deterministic-stochastic oncolytic M1 model involving immune response using piecewsie fractional differential equation technique. Shah et al.^40^ expounded the coupled system of drug therapy via piecewise fractional differential equations. Arik and Araz^41^ crossover behaviors via piecewise concept: a model of tumor growth and its response to radiotherapy.

It is apparent that the disease transmitted by rodents has data pertaining to its prior phases and an instructional mechanism; to be more particular, memory plays a crucial part in vector-borne disease transmission dynamics. The host population’s memory correlates with personal consciousness, thereby lowering the contact rate between vectors and hosts, whereas rodents employ previous information about the human’s location, blood selection, colour, and the smell of human sweat^38,39^. In mathematical modelling of infectious diseases, these sorts of phenomena can be readily represented by a fractional-order system. It ought to be additionally highlighted that a majority of real-world phenomena are not merely predictable, considering the outcome of an analysis is completely dictated by the attribute information and initial conditions. Uncertainty is a characteristic of random systems. The identical assortment of parameter settings and initial conditions will result in a combination with multiple outcomes. In a nutshell, predetermined designs constitute a system that takes numerals as components and generates information as the results. A randomly generated simulation involves a random element that takes an assortment as a source of information and produces a circulation as a consequence. These patterns of distribution might indicate the degree of unpredictability in the information being supplied (for example, predictable suggestions along with noise) or an arbitrary procedure (for example, randomly generated data)^42,43^.

Mathematical modeling is regarded as a crucial instrument to illustrate the evolving behaviors of various prevalent illnesses. Multiple epidemiological models for figuring out and managing multiple prevalent illnesses in a specific area are being constructed by various scientists and environmentalists. In the last two decades, numerical modeling has been extensively employed to characterize the transmission of multiple illnesses (see, for example,^44^). Different understandings are currently being investigated to broaden the knowledge of the Lassa fever, specifically capturing its significant deductions via predictive modeling^45^. Simulations define the fluctuating course of transmission; even so, because of their pragmatic strategy, stochastic differential equations are suitable for modelling biological phenomena. When juxtaposed with deterministic designs, stochastic algorithms produce superior outcomes because, after multiple runs of operation, a distribution of the anticipated outcomes, including the mean of ailments at any moment ${\textbf{t}}$, is capable of being developed, while deterministic frameworks produce one estimated value^39,46^. There are plenty of strategies and techniques for examining stochastic systems^47^.

Our research is an extension of the work of Peter et al.^48^ and Ibrahim and Denes^49^ by: (i) implementing ecological contaminants into an individual’s dissemination route. The context is defined by the substrates, buildings, and additional supplies in which the infectious agent is stored. (ii) Launching pollutants into an individual’s dissemination paths. By airborne particles, we mean airborne substances that have been focused on individuals and atmospheric activity. These two channels weren’t typically regarded as receiving massive amounts of sick drivers. Taking into account the Lassa fever transmission structure, we proposed a stochastic perturbation technique to simulate the propagation evolution of the Lassa fever that includes differing individual settings for long-term behaviour, employing the existing research on modelling outbreaks. We divide the entire community into ten different groups. Consequently, our research focuses on (i) human-to-human transmission, (ii) rodent-to-human transmission, (iii) rodent-to-rodent transmission, (iv) environment-to-human transmission, (v) aerosol to human transmission and (vi) environment to rodent transmission. These investigations constitute the cornerstone of our research with the piecewise differential equation techniques, and the knowledge acquired compared to them will assist us in developing and analyzing a broader investigation regarding additional dissemination channels when white noise and random perturbations are involved.

## Model configuration

The cumulative human community, denoted as ${\textbf{N}}_{{\textbf{h}}}({\textbf{t}})$, is classified into five categories, including those who are vulnerable to the pathogen, or ${\textbf{X}}_{{\textbf{h}}}({\textbf{t}})$, individuals who carry the pathogen but aren’t contagious, ${\textbf{P}}_{{\textbf{h}}}({\textbf{t}})$, those who are contagious yet do not exhibit symptoms, ${\textbf{Q}}_{{\textbf{h}}{\textbf{a}}}({\textbf{t}})$, those who are contagious but have symptoms, ${\textbf{Q}}_{{\textbf{h}}{\textbf{s}}}({\textbf{t}})$ and individuals who have healed from Lassa fever, or ${\textbf{R}}_{{\textbf{h}}}({\textbf{t}})$ are presented as:

1 $$\begin{aligned} {\textbf{N}}_{{\textbf{h}}}({\textbf{t}})={\textbf{X}}_{{\textbf{h}}} ({\textbf{t}})+{\textbf{P}}_{{\textbf{h}}}({\textbf{t}}) +{\textbf{Q}}_{{\textbf{h}}{\textbf{a}}}({\textbf{t}}) +{\textbf{Q}}_{{\textbf{h}}{\textbf{s}}}({\textbf{t}}) +{\textbf{R}}_{{\textbf{h}}}({\textbf{t}}). \end{aligned}$$

The overall rodent community, denoted as ${\textbf{N}}_{{\textbf{r}}}({\textbf{t}})$, is categorized as follows: rodents vulnerable to the pathogen, denoted as ${\textbf{X}}_{{\textbf{r}}}({\textbf{t}})$ rodents contaminated well with Lassa virus infection and not contagious, denoted as ${\textbf{P}}_{{\textbf{r}}}({\textbf{t}})$ and contaminated rodents, denoted as ${\textbf{N}}_{{\textbf{r}}}({\textbf{t}})$ to:

2 $$\begin{aligned} {\textbf{N}}_{{\textbf{r}}}({\textbf{t}}) ={\textbf{X}}_{{\textbf{r}}}({\textbf{t}}) +{\textbf{P}}_{{\textbf{r}}}({\textbf{t}}) +{\textbf{Q}}_{{\textbf{r}}}({\textbf{t}}). \end{aligned}$$

We take into consideration the aforementioned limited propagation routes: human-to-human, rodent-to-human and rodent-to-rodent. We also take into account informal pathogens like E-H interaction, A-H interaction and E-R interaction. We employ ${\textbf{G}}_{{\textbf{s}}}$ to represent the accumulation of the Lassa fever pathogen on ecological interfaces and ${\textbf{G}}_{{\textbf{a}}}$ to represent the accumulation of the viral infection in the atmosphere and as such, to account for unintended propagation mechanisms, where ${\textbf{G}}_{{\textbf{s}}}, {\textbf{G}}_{{\textbf{a}}}$, provides the highest pathogen maximum load on interfaces and equipment and in the atmosphere is presented by $\Phi _{{\textbf{v}}}$ with ${\textbf{G}}_{{\textbf{a}}}\le \Phi _{{\textbf{v}}}.$

**Figure 2:**
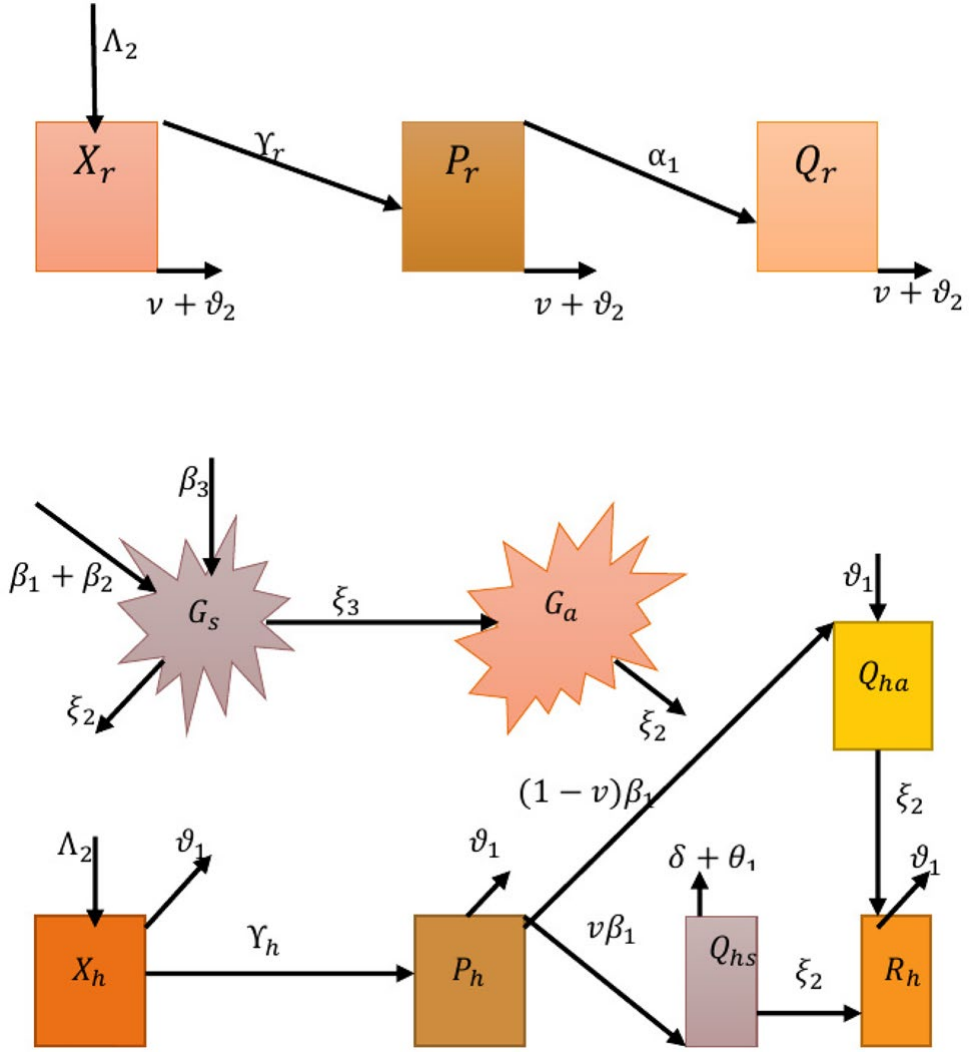
Schematic view of Lassa fever model.

We presume that $\Lambda _{1}$ represents the steady rate of vulnerable living organisms recruiting new members. Throughout an infectious disease outbreak, the vulnerable people advance to the exposure group ${\textbf{P}}_{{\textbf{h}}}$ as described $\Upsilon _{{\textbf{h}}}=\gamma _{{\textbf{h}}}\Big (\frac{{\textbf{Q}}_ {{\textbf{r}}}}{{\textbf{N}}_{{\textbf{r}}}}+\frac{\rho _{1}{\textbf{Q}}_ {{\textbf{h}}{\textbf{s}}}}{{\textbf{N}}_{{\textbf{h}}}}+\frac{\rho _{2} {\textbf{Q}}_{{\textbf{h}}{\textbf{a}}}}{{\textbf{N}}_{{\textbf{h}}}} +\frac{\rho _{3}{\textbf{G}}_{{\textbf{s}}}}{\Phi _{{\textbf{v}}}} +\frac{\rho _{4}{\textbf{G}}_{{\textbf{a}}}}{\Phi _{{\textbf{v}}}}\Big ).$

Here, $\rho _{1}$ is the reconfiguration value which suggests interaction with ${\textbf{Q}}_{{\textbf{h}}{\textbf{s}}}$ is less contagious than interacting to ${\textbf{Q}}_{{\textbf{r}}}$. $\gamma _{{\textbf{h}}}$ is the enhanced surface rate between highly vulnerable individuals and afflicted rodents, vulnerable beings and contagious beings, vulnerable beings, the viral disease in the atmosphere, and the pathogen in the atmosphere. In this manner, the adjustment specifications $\rho _{2},\rho _{3}$ and $\rho _{4}$ also consider the degree of reinfection of interaction with ${\textbf{Q}}_{{\textbf{h}}{\textbf{a}}},$ ${\textbf{G}}_{{\textbf{s}}}$ and ${\textbf{G}}_{{\textbf{a}}}$, respectively. Indications from the guarantees of inclusivity stated as $\rho _{4}<\rho _{3}<\rho _{1}<\rho _{2}<1.$

The unprotected individuals advance to the contagious cohort at a speed of $\alpha _{1}$, where $\mu \alpha _{1}$ is the ratio of affected populations who are latent and $(1-\mu )\alpha _{1}$ is the fraction who develop symptoms. All categories of individuals instinctually pass away at the speed $\vartheta _{1}$. Individuals who are infectiously indicative can pass away from the ailment at a speed of $\delta$, whereas there are no incidences of infectiously subclinical people passing away from the infestation. Individuals who are infectiously displaying symptoms or not come back at rates of $\phi _{1}$ and $\phi _{2}$, respectively. Throughout a power of infestation, the vulnerable rodents are attracted to the unprotected class ${\textbf{P}}_{{\textbf{r}}}$ at a steady rate $\Lambda _{2}$ and relocate there for $\Upsilon _{{\textbf{r}}}=\gamma _{{\textbf{r}}}\Big (\frac{{\textbf{Q}}_ {{\textbf{r}}}}{{\textbf{N}}_{{\textbf{r}}}}+\frac{\phi _{1}{\textbf{G}}_ {{\textbf{s}}}}{\Phi _{{\textbf{v}}}}\Big ),$ where $\gamma _{{\textbf{r}}}$ is the proportion of efficacious interaction between rodents that are vulnerable to getting sick and afflicted rodents, as well as between vulnerable rodents and potentially polluted in the surroundings. The modifying variable, $\phi _{1}$, demonstrates that interaction with ${\textbf{G}}_{{\textbf{s}}}$ becomes less contagious than interaction with ${\textbf{Q}}_{{\textbf{r}}}$. All rodents inherently pass away at a speed of $\vartheta _{2}$, and unprotected rodents transition to the contagious category at a rate of $\alpha _{2}$. Because they are consumed by human beings as meals, rodents can indeed perish at a rate of $\upsilon$. Since afflicted rodents can persist to absorb the pathogen for the rest of their lives, pathogens do not cause rodents to drop dead. By urinating, excreting feces, haemorrhage, and secreting mucus, afflicted rodents, contagious indicative beings, and contagious symptom less beings, respectively, release the Lassa fever pathogen into the surroundings at rates of $\beta _{1}$, $\beta _{2}$ and $\beta _{3}$, respectively. We also make the assumption that a component of the pathogen accumulation advances into the atmosphere via air flow and anthropogenic at a rate of $\xi _{2}$, whereas the remaining pathogen accumulation degrades on contaminated interfaces and in the atmosphere at a rate of $\xi _{2}$ and $\xi _{3}$, respectively. The first order nonlinear ordinary differential equations that represent the Lassa fever framework in Fig. 2 are as follows:

3 $$\begin{aligned} {\left\{ \begin{array}{ll} \dot{{\textbf{X}}_{{\textbf{h}}}}=\Lambda _{1}-\Upsilon _{{\textbf{h}}} {\textbf{X}}_{{\textbf{h}}}-\vartheta _{1}{\textbf{X}}_{{\textbf{h}}},\\ \dot{{\textbf{P}}_{{\textbf{h}}}}=\Upsilon _{{\textbf{h}}}{\textbf{X}}_{{\textbf{h}}}-(\alpha _{1}+\vartheta _{1}){\textbf{P}}_{{\textbf{h}}},\\ \dot{{\textbf{Q}}_{{\textbf{h}}{\textbf{a}}}}=\mu \alpha _{1}{\textbf{P}}_{{\textbf{h}}}-(\phi _{1}+\vartheta _{1}){\textbf{Q}}_{{\textbf{h}}{\textbf{a}}},\\ \dot{{\textbf{Q}}_{{\textbf{h}}{\textbf{s}}}}=(1-\mu )\alpha _{1}{\textbf{P}}_{{\textbf{h}}}-(\delta +\phi _{2}+\vartheta _{1}){\textbf{Q}}_{{\textbf{h}}{\textbf{s}}},\\ \dot{{\textbf{R}}_{{\textbf{h}}}}=\phi _{1}{\textbf{P}}_{{\textbf{h}}}+\phi _{2}{\textbf{Q}}_{{\textbf{h}}{\textbf{s}}}-\vartheta _{1}{\textbf{R}}_{{\textbf{h}}},\\ \dot{{\textbf{X}}_{{\textbf{r}}}}=\Lambda _{2}-\Upsilon _{{\textbf{r}}}{\textbf{X}}_{{\textbf{r}}}-(\vartheta _{2}+\upsilon ){\textbf{R}}_{{\textbf{h}}},\\ \dot{{\textbf{P}}_{{\textbf{r}}}}=\Upsilon _{{\textbf{r}}}{\textbf{X}}_{{\textbf{r}}}-(\alpha _{2}+\vartheta _{2}+\upsilon ){\textbf{P}}_{{\textbf{h}}},\\ \dot{{\textbf{Q}}_{{\textbf{r}}}}=\alpha _{2}{\textbf{P}}_{{\textbf{r}}}-(\upsilon +\vartheta _{2}){\textbf{Q}}_{{\textbf{r}}},\\ \dot{{\textbf{G}}_{{\textbf{s}}}}=\beta _{1}{\textbf{Q}}_{{\textbf{h}}{\textbf{a}}}+\beta _{2}{\textbf{Q}}_{{\textbf{h}}{\textbf{s}}}+\beta _{3}{\textbf{Q}}_{{\textbf{r}}}-(\xi _{2}+\xi _{3}){\textbf{G}}_{{\textbf{s}}},\\ \dot{{\textbf{G}}_{{\textbf{a}}}}=\xi _{3}{\textbf{G}}_{{\textbf{s}}}-\xi _{2}{\textbf{G}}_{{\textbf{a}}}, \\ \end{array}\right. } \end{aligned}$$

supplemented by the initial conditions (initial conditions):

4 $$\begin{aligned}{} & {} {\textbf{X}}_{{\textbf{h}}}(0)={\textbf{X}}_{{{\textbf{h}}}_{0}} \ge 0,~~{\textbf{P}}_{{\textbf{h}}}(0)={\textbf{P}}_{{{\textbf{h}}}_{0}} \ge 0,~~{\textbf{Q}}_{{\textbf{h}}{\textbf{a}}}(0)={\textbf{Q}}_{{{\textbf{h}}{\textbf{a}}}_{0}} \ge 0,~~{\textbf{Q}}_{{\textbf{h}}{\textbf{s}}}(0)={\textbf{Q}}_{{{\textbf{h}}{\textbf{s}}}_{0}}\ge 0,\nonumber \\{} & {} {\textbf{R}}_{{\textbf{h}}}(0)={\textbf{R}}_{{{\textbf{h}}}_{0}} \ge 0,~~{\textbf{X}}_{{\textbf{r}}}(0)={\textbf{X}}_{{{\textbf{r}}}_{0}}\ge 0,~~ {\textbf{P}}_{{\textbf{r}}}(0)={\textbf{P}}_{{{\textbf{r}}}_{0}}\ge 0,~~{\textbf{Q}}_ {{\textbf{r}}}(0)={\textbf{Q}}_{{{\textbf{r}}}_{0}}\ge 0,\nonumber \\{} & {} {\textbf{G}}_ {{\textbf{s}}}(0)={\textbf{G}}_{{{\textbf{s}}}_{0}}\ge 0,~~{\textbf{G}}_{{\textbf{a}}}(0) ={\textbf{G}}_{{{\textbf{a}}}_{0}}\ge 0,~\forall ~{\textbf{t}}\ge 0. \end{aligned}$$

Table 1 displays the elements and representations for the parameters and their variables.

**Table 1:** Explanation of system’s feature.

*Symbols*	Explanation	Values
$\mu$	Percentage of people who develop to ${\textbf{Q}}_{{\textbf{h}}{\textbf{a}}}$	Nil
$\delta$	Mortality from infection for people	Day$^{-1}$
$\Phi _{{\textbf{v}}}$	The greatest infection transmission rate	Virus
$\Lambda _{1}$	Individuals recruiting quantity	Human/day
$\Lambda _{2}$	Rodents recruiting quantity	Rodents/day
$\vartheta _{1}$	Individuals natural mortality rate	Day$^{-1}$
$\vartheta _{2}$	Rodents natural mortality rate	Day$^{-1}$
$\phi _{1}$	${\textbf{Q}}_{{\textbf{h}}{\textbf{a}}}$ rate of recuperation	Day$^{-1}$
$\phi _{2}$	${\textbf{Q}}_{{\textbf{h}}{\textbf{s}}}$ rate of recuperation	Day$^{-1}$
$\upsilon$	Rationale for rodent mortality resulting from human intake	Day$^{-1}$
$\xi _{2}$	Rate of infection deterioration in $V_{{\textbf{s}}}$	Day$^{-1}$
$\xi _{3}$	The speed of infection development from $V_{{\textbf{s}}}$ to $V_{{\textbf{a}}}$	Day$^{-1}$
$\beta _{1}$	The speed at which ${\textbf{Q}}_{{\textbf{h}}{\textbf{a}}}$ releases infection in ${\textbf{G}}_{{\textbf{s}}}$	Infection/individual $\times$day$^{-1}$
$\beta _{2}$	The speed at which ${\textbf{Q}}_{{\textbf{h}}{\textbf{s}}}$ releases infection in ${\textbf{G}}_{{\textbf{s}}}$	Infection/individual$\times$day$^{-1}$
$\beta _{3}$	The speed at which ${\textbf{Q}}_{{\textbf{r}}}$ releases infection in ${\textbf{G}}_{{\textbf{s}}}$	Infection/individual$\times$day$^{-1}$
$\alpha _{1}$	Individuals’ rate of switching from ${\textbf{P}}_{{\textbf{h}}}$ to ${\textbf{Q}}_{{\textbf{h}}{\textbf{a}}}$ and ${\textbf{Q}}_{{\textbf{h}}{\textbf{s}}}$	Day$^{-1}$
$\alpha _{2}$	Rodents’ rate of switching from ${\textbf{P}}_ {{\textbf{r}}}$ to ${\textbf{Q}}_{{\textbf{r}}}$	Day$^{-1}$
$\gamma _{{\textbf{r}}}$	Rate for interaction among ${\textbf{X}}_{{\textbf{r}}}, {\textbf{Q}}_{{\textbf{r}}}$ and ${\textbf{G}}_{{\textbf{s}}}$	Day$^{-1}$
$\gamma _{{\textbf{h}}}$	Rate for interaction among ${\textbf{X}}_{{\textbf{h}}}, {\textbf{Q}}_{{\textbf{h}}{\textbf{a}}}, {\textbf{Q}}_{{\textbf{h}} {\textbf{s}}}, {\textbf{Q}}_{{\textbf{r}}}, {\textbf{G}}_{{\textbf{a}}}$ and ${\textbf{G}}_{{\textbf{s}}}$	Day$^{-1}$
$\rho _{1}$	An altered factor	Nil
$\rho _{2}$	An altered factor	Nil
$\rho _{3}$	An altered factor	Nil
$\rho _{4}$	An altered factor	Nil

However, in the framework (3), it is assumed that people currently reside in a steady environment. Even so, the perturbation in the surroundings will indeed influence certain aspects of the outbreak model’s process variables. Having subsequently discovered that the stochastic framework can further adequately represent biological mechanisms and viral infections, there has been a significant rise in enthusiasm for taking random perturbation into account in virology configurations^42,43^. The framework can currently be perturbed stochastically in a variety of manners. Assuming that random perturbations constitute a single sort of white noise that is proportional to every component, respectively, comprises one of the most crucial steps. Considering the foregoing, it really is supposed in the proposed investigation that the white noise is individually proportional to the compartments ${\textbf{X}}_{{\textbf{h}}}^{*},{\textbf{P}}_{{\textbf{h}}}^{*},{\textbf{Q}}_{{\textbf{h}}{\textbf{a}}}^{*},{\textbf{Q}}_{{\textbf{h}}{\textbf{s}}}^{*},{\textbf{R}}_{{\textbf{h}}}^{*},{\textbf{X}}_{{\textbf{r}}}^{*},{\textbf{P}}_{{\textbf{r}}}^{*},{\textbf{Q}}_{{\textbf{r}}}^{*},{\textbf{G}}_{{\textbf{s}}}^{*}$ and ${\textbf{G}}_{{\textbf{a}}}^{*},$ respectively. Regarding that, the dynamical framework presumes the respective structure, linking the deterministic framework (3):

5 $$\begin{aligned} {\left\{ \begin{array}{ll} d{\textbf{X}}_{{\textbf{h}}}=\big [\Lambda _{1}-\Upsilon _{{\textbf{h}}}{\textbf{X}}_{{\textbf{h}}}-\vartheta _{1}{\textbf{X}}_{{\textbf{h}}}\big ]d{\textbf{t}}+\sigma _{1}{\textbf{X}}_{{\textbf{h}}}d{\mathcal {B}}_{1}({\textbf{t}}),\\ d{\textbf{P}}_{{\textbf{h}}}=\big [\Upsilon _{{\textbf{h}}}{\textbf{X}}_{{\textbf{h}}}-(\alpha _{1}+\vartheta _{1}){\textbf{P}}_{{\textbf{h}}}\big ]d{\textbf{t}}+\sigma _{2}{\textbf{P}}_{{\textbf{h}}}d{\mathcal {B}}_{2}({\textbf{t}}),\\ d{\textbf{Q}}_{{\textbf{h}}{\textbf{a}}}=\big [\mu \alpha _{1}{\textbf{P}}_{{\textbf{h}}}-(\phi _{1}+\vartheta _{1}){\textbf{Q}}_{{\textbf{h}}{\textbf{a}}}\big ]d{\textbf{t}}+\sigma _{3}{\textbf{Q}}_{{\textbf{h}}{\textbf{a}}}d{\mathcal {B}}_{3}({\textbf{t}}),\\ d{\textbf{Q}}_{{\textbf{h}}{\textbf{s}}}=\big [(1-\mu )\alpha _{1}{\textbf{P}}_{{\textbf{h}}}-(\delta +\phi _{2}+\vartheta _{1}){\textbf{Q}}_{{\textbf{h}}{\textbf{s}}}\big ]d{\textbf{t}}+\sigma _{4}{\textbf{Q}}_{{\textbf{h}}{\textbf{s}}}d{\mathcal {B}}_{4}({\textbf{t}}),\\ d{\textbf{R}}_{{\textbf{h}}}=\big [\phi _{1}{\textbf{P}}_{{\textbf{h}}}+\phi _{2}{\textbf{Q}}_{{\textbf{h}}{\textbf{s}}}-\vartheta _{1}{\textbf{R}}_{{\textbf{h}}}\big ]d{\textbf{t}}+\sigma _{5}{\textbf{R}}_{{\textbf{h}}}d{\mathcal {B}}_{5}({\textbf{t}}),\\ d{\textbf{X}}_{{\textbf{r}}}=\big [\Lambda _{2}-\Upsilon _{{\textbf{r}}}{\textbf{X}}_{{\textbf{r}}}-(\vartheta _{2}+\upsilon ){\textbf{R}}_{{\textbf{h}}}\big ]d{\textbf{t}}+\sigma _{6}{\textbf{X}}_{{\textbf{r}}}d{\mathcal {B}}_{6}({\textbf{t}}),\\ d{\textbf{P}}_{{\textbf{r}}}=\big [\Upsilon _{{\textbf{r}}}{\textbf{X}}_{{\textbf{r}}}-(\alpha _{2}+\vartheta _{2}+\upsilon ){\textbf{P}}_{{\textbf{h}}}\big ]d{\textbf{t}}+\sigma _{7}{\textbf{P}}_{{\textbf{r}}}d{\mathcal {B}}_{7}({\textbf{t}}),\\ d{\textbf{Q}}_{{\textbf{r}}}=\big [\alpha _{2}{\textbf{P}}_{{\textbf{r}}}-(\upsilon +\vartheta _{2}){\textbf{Q}}_{{\textbf{r}}}\big ]d{\textbf{t}}+\sigma _{8}{\textbf{Q}}_{{\textbf{r}}}d{\mathcal {B}}_{8}({\textbf{t}}),\\ d{\textbf{G}}_{{\textbf{s}}}=\big [\beta _{1}{\textbf{Q}}_{{\textbf{h}}{\textbf{a}}}+\beta _{2}{\textbf{Q}}_{{\textbf{h}}{\textbf{s}}}+\beta _{3}{\textbf{Q}}_{{\textbf{r}}}-(\xi _{2}+\xi _{3}){\textbf{G}}_{{\textbf{s}}}\big ]d{\textbf{t}}+\sigma _{9}{\textbf{G}}_{{\textbf{s}}}d{\mathcal {B}}_{9}({\textbf{t}}),\\ d{\textbf{G}}_{{\textbf{a}}}=\big [\xi _{3}{\textbf{G}}_{{\textbf{s}}}-\xi _{2}{\textbf{G}}_{{\textbf{a}}}\big ]d{\textbf{t}}+\sigma _{10}{\textbf{G}}_{{\textbf{a}}}d{\mathcal {B}}_{10}({\textbf{t}}),\end{array}\right. } \end{aligned}$$

Where ${\mathcal {B}}_{{\textbf{m}}}({\textbf{t}}),~{\textbf{m}}=1,...,10$ are mutually independent standard Brownian motions described on a complete probability space $(\Pi , {\mathfrak {F}},\{{\mathfrak {F}}_{{\textbf{t}}}\}_{{\textbf{t}}\ge 0},{\mathbb {P}})$ with a $\{{\mathfrak {F}}_{{\textbf{t}}}\}_{{\textbf{t}}\ge 0}$ filtration entertaining the regular requirements^50^, and $\sigma _{{\textbf{m}}},~~{\textbf{m}}=1,...,10$ represents the intensity of white noises ${\mathcal {B}}_{{\textbf{m}}},~({\textbf{m}}=1,...,10)$, respectively).

For the sake of inconvenience, we use the following symbols:

$$\Re _{+}^{\bar{d}}=\big \{\bar{x}=(x_{1},...,x_{\bar{d}})^{\bar{T}} \in \Re ^{\bar{d}};~\bar{x}_{\iota }>0,~\iota \in [1,\bar{d}]\big \},~~{\bar{\Re }}_{+} ^{\bar{d}}=\big \{\bar{x}=(x_{1},...,x_{\bar{d}})^{\bar{T}}\in \Re ^{\bar{d}};~ \bar{x}_{\iota }\ge 0,~\iota \in [1,\bar{d}]\big \}.$$

For any $$\bar{x},\bar{y}\in \Re , ~then~\bar{x}\vee \bar{y}=\max \{\bar{x},\bar{y}\}~and ~ \bar{x}\wedge \bar{y}=\min \{\bar{x},\bar{y}\}.$$

The stochastic differential equation in ${\mathfrak {d}}$-dimensions is presented below:

6 $$\begin{aligned} d{\textbf{v}}(\zeta )={\textbf{u}}({\textbf{v}}(\zeta ),\zeta )d\zeta +{\textbf{q}}({\textbf{v}}(\zeta ),\zeta )d{\mathcal {B}}(\zeta ),~{\textbf{v}}(\zeta _{0})={\textbf{v}}_{0},~\forall ~\zeta _{0}\le \zeta \le {\tilde{T}}<\infty , \end{aligned}$$

where ${\textbf{u}}:\Re ^{{\mathfrak {d}}}\times [\zeta _{0},{\tilde{T}}]\mapsto \Re ^{{\mathfrak {d}}}$ and ${\textbf{q}}:\Re ^{{\mathfrak {d}}}\times [\zeta _{0},{\tilde{T}}]\mapsto \Re ^{{\mathfrak {d}}\times m_{1}}$ are Borel measurable having ${\mathcal {B}}=\{{\mathcal {B}}(\zeta )\}_{\zeta \ge \zeta _{0}}$ is an $\Re ^{m_{1}}$-valued Wiener process, and ${\textbf{v}}_{0}$ is an $\Re ^{{\mathfrak {d}}}$-valued random variable presented as $\Omega .$

Therefore, $\bar{C}^{2,1}(\Re ^{{\mathfrak {d}}}\times [\zeta _{0},\infty );\Re _{+})$ is considered as the family of all non-negative functions ${\mathcal {V}}({\textbf{v}},\zeta )$ on $\Re ^{{\mathfrak {d}}}\times [\zeta _{0},\infty )$ that are continuously twice differentiable in ${\textbf{v}}\in \Re ^{{\mathfrak {d}}}$ and once in $\zeta \in [\zeta _{0},\infty )$. The differential formulation ${\mathbb {L}}$ for the stochastic differential Eq. (6) is given as

$$\begin{aligned} {\mathbb {L}}=\frac{\partial }{\partial \zeta }+\sum \limits _{\varsigma =1}^{{\mathfrak {d}}}{\textbf{u}}_{\varsigma }({\textbf{v}},\zeta )\frac{\partial }{\partial {\textbf{v}}_{\varsigma }}+\frac{1}{2}\sum \limits _{{\textbf{i}},\varsigma =1}^{{\mathfrak {d}}}\sum \limits _{{\textbf{m}}=1}^{m_{1}}{{\textbf{q}}}_{\varsigma {\textbf{m}}}({\textbf{v}},\zeta ){{\textbf{q}}}_{\varsigma {\textbf{m}}}({\textbf{v}},\zeta )\frac{\partial ^{2}}{\partial {\textbf{v}}_{\varsigma }\partial {\textbf{v}}_{{\textbf{i}}}}. \end{aligned}$$

Introducing the functional ${\mathcal {V}}\in \bar{C}^{2,1}(\Re ^{{\mathfrak {d}}}\times [\zeta _{0},\infty ),$ then

$$\begin{aligned} {\mathbb {L}}{\mathcal {V}}({\textbf{v}},\zeta )={\mathcal {V}}_{\zeta }({\textbf{v}},\zeta )+{\mathcal {V}}_{{\textbf{v}}}({\textbf{v}},\zeta ){\textbf{f}}({\textbf{v}},\zeta )+\frac{1}{2}\sum \limits _{{\textbf{i}},\varsigma =1}^{{\mathfrak {d}}}\sum \limits _{{\textbf{m}}=1}^{m_{1}}{{\textbf{q}}}_{{\textbf{i}}{\textbf{m}}}({\textbf{v}},\zeta ){{\textbf{g}}}_{\varsigma {\textbf{m}}}({\textbf{v}},\zeta ){\mathcal {V}}_{{\textbf{v}}{\textbf{v}}}({\textbf{v}},\zeta ), \end{aligned}$$

where $${\mathcal {V}}_{\zeta }:=\frac{\partial {\mathcal {V}}}{\partial \zeta };~{\mathcal {V}}_{{\textbf{s}}_{1}}=({\mathcal {V}}_{{\textbf{v}}_{\varsigma }},...,{\mathcal {V}}_{{\textbf{v}}_{{\mathfrak {d}}}}),~~{\mathcal {V}}_{{\textbf{v}}{\textbf{v}}}=({\mathcal {V}}_{{\textbf{v}}_{\varsigma }},{\mathcal {V}}_{{\textbf{v}}_{\varsigma }})_{{\mathfrak {d}}\times {\mathfrak {d}}}.$$

For ${\textbf{v}}(\zeta )\in \Re ^{{\mathfrak {d}}},$ then Itô’s method can be described as:

$$\begin{aligned} d{\mathcal {V}}({\textbf{v}}(\zeta ),\zeta )={\mathbb {L}}{\mathcal {V}}({\textbf{v}}(\zeta ),\zeta ) d\zeta +{\mathcal {V}}_{{\textbf{v}}}({\textbf{v}}(\zeta ),\zeta ){{\textbf{q}}}({\textbf{v}}(\zeta ),\zeta )d{\mathcal {B}}(\zeta ). \end{aligned}$$

Here, we furnish the associated overview here to assist viewers who are familiar with FC (see;^20,34,35^).

$$\begin{aligned} \,_{0}^{C}{\textbf{D}}_{\zeta }^{\chi } {\mathcal {G}}(\zeta )=\frac{1}{\Gamma (1-\chi )}\int \limits _{0}^{\zeta }{\mathcal {G}}^{\prime }({\textbf{w}})(\zeta -{\textbf{w}})^{\chi }d{\textbf{w}},~~\chi \in (0,1]. \end{aligned}$$

The Caputo fractional derivative involves the power-law function. The Caputo fractional-order derivative allows usual initial conditions when playing with the integral transform, for instance the Laplace transform^51,52^.

$$\begin{aligned} \,_{0}^{CF}{\textbf{D}}_{\zeta }^{\chi } {\mathcal {G}}(\zeta )=\frac{\bar{{\mathcal {M}}}(\chi )}{1-\chi }\int \limits _{0}^{\zeta }{\mathcal {G}}^{\prime }({\textbf{w}})\exp \bigg [-\frac{\chi }{1-\chi }(\zeta -{\textbf{w}})\bigg ]d{\textbf{w}},~~\chi \in (0,1], \end{aligned}$$

where $\bar{{\mathcal {M}}}(\chi )$ is stated to be normalized mapping with $\bar{{\mathcal {M}}}(0)=\bar{{\mathcal {M}}}(1)=1.$

The Caputo-Fabrizio operator which has attracted many research scholars due to the fact that it has a non-singular kernel. Also the Caputo-Fabrizio operator is most appropriate for modeling some class of real-world problem which follows the exponential decay law^53^. With the passage of time, developing a mathematical model using the Caputo-Fabrizio fractional-order derivative became a remarkable field of research. In recent times, several mathematicians were busy in development and simulation of Caputo-Fabrizio fractional differential equations^54^.

The fractional derivative operator of the Atangana-Baleanu of Caputo type is defined as:

$$\begin{aligned} \,_{0}^{ABC}{\textbf{D}}_{\zeta }^{\chi } {\mathcal {G}}(\zeta )=\frac{ABC(\chi )}{1-\chi }\int \limits _{0}^{\zeta }{\mathcal {G}}^{\prime }({\textbf{w}})E_{\chi }\bigg [-\frac{\chi }{1-\chi }(\zeta -{\textbf{w}})^{\chi }\bigg ]d{\textbf{w}},~~\chi \in (0,1], \end{aligned}$$

where $ABC(\chi )=1-\chi +\frac{\chi }{\Gamma (\chi )}$ indicates the normalization mapping.

The kernel used in Atangana-Baleanu fractional differentiation appears naturally in several physical problems as generalized exponential decay and as a power-law asymptotic for a very large time^55,56^. The choice of this derivative is motivated by the fact that the interaction is not local, but global, and also, the trend observed in the field does not follow the power-law. The generalized Mittag-Leffler function completely induced the effect of memory, which is very important in the nonlinear Baggs-Freedman model^57^.

As fractional-order models describe the non-local behavior of biological systems and posses hereditary property, moreover, it provides information about its past and present state for the future, therefore, we represent the dynamical system (3) of Lassa virus in the framework of fractional-order Caputo’s derivative to conceptualize the transmission of Lassa fever in a more accurate way. Thus, the system consist of fractional derivatives is presented by

7 $$\begin{aligned} {\left\{ \begin{array}{ll} \,_{0}^{c}{\textbf{D}}_{{\textbf{t}}}^{\chi }{{\textbf{X}}_{{\textbf{h}}}}=\Lambda _{1}-\Upsilon _{{\textbf{h}}}{\textbf{X}}_{{\textbf{h}}}-\vartheta _{1}{\textbf{X}}_{{\textbf{h}}},\\ \,_{0}^{c}{\textbf{D}}_{{\textbf{t}}}^{\chi }{{\textbf{P}}_{{\textbf{h}}}}=\Upsilon _{{\textbf{h}}}{\textbf{X}}_{{\textbf{h}}}-(\alpha _{1}+\vartheta _{1}){\textbf{P}}_{{\textbf{h}}},\\ \,_{0}^{c}{\textbf{D}}_{{\textbf{t}}}^{\chi }{{\textbf{Q}}_{{\textbf{h}}{\textbf{a}}}}=\mu \alpha _{1}{\textbf{P}}_{{\textbf{h}}}-(\phi _{1}+\vartheta _{1}){\textbf{Q}}_{{\textbf{h}}{\textbf{a}}},\\ \,_{0}^{c}{\textbf{D}}_{{\textbf{t}}}^{\chi }{{\textbf{Q}}_{{\textbf{h}}{\textbf{s}}}}=(1-\mu )\alpha _{1}{\textbf{P}}_{{\textbf{h}}}-(\delta +\phi _{2}+\vartheta _{1}){\textbf{Q}}_{{\textbf{h}}{\textbf{s}}},\\ \,_{0}^{c}{\textbf{D}}_{{\textbf{t}}}^{\chi }{{\textbf{R}}_{{\textbf{h}}}}=\phi _{1}{\textbf{P}}_{{\textbf{h}}}+\phi _{2}{\textbf{Q}}_{{\textbf{h}}{\textbf{s}}}-\vartheta _{1}{\textbf{R}}_{{\textbf{h}}},\\ \,_{0}^{c}{\textbf{D}}_{{\textbf{t}}}^{\chi }{{\textbf{X}}_{{\textbf{r}}}}=\Lambda _{2}-\Upsilon _{{\textbf{r}}}{\textbf{X}}_{{\textbf{r}}}-(\vartheta _{2}+\upsilon ){\textbf{R}}_{{\textbf{h}}},\\ \,_{0}^{c}{\textbf{D}}_{{\textbf{t}}}^{\chi }{{\textbf{P}}_{{\textbf{r}}}}=\Upsilon _{{\textbf{r}}}{\textbf{X}}_{{\textbf{r}}}-(\alpha _{2}+\vartheta _{2}+\upsilon ){\textbf{P}}_{{\textbf{h}}},\\ \,_{0}^{c}{\textbf{D}}_{{\textbf{t}}}^{\chi }{{\textbf{Q}}_{{\textbf{r}}}}=\alpha _{2}{\textbf{P}}_{{\textbf{r}}}-(\upsilon +\vartheta _{2}){\textbf{Q}}_{{\textbf{r}}},\\ \,_{0}^{c}{\textbf{D}}_{{\textbf{t}}}^{\chi }{{\textbf{G}}_{{\textbf{s}}}}=\beta _{1}{\textbf{Q}}_{{\textbf{h}}{\textbf{a}}}+\beta _{2}{\textbf{Q}}_{{\textbf{h}}{\textbf{s}}}+\beta _{3}{\textbf{Q}}_{{\textbf{r}}}-(\xi _{2}+\xi _{3}){\textbf{G}}_{{\textbf{s}}},\\ \,_{0}^{c}{\textbf{D}}_{{\textbf{t}}}^{\chi }{{\textbf{G}}_{{\textbf{a}}}}=\xi _{3}{\textbf{G}}_{{\textbf{s}}}-\xi _{2}{\textbf{G}}_{{\textbf{a}}}. \end{array}\right. } \end{aligned}$$

The structure of this essay can be described as follows: In Section “Model configuration”, we demonstrate that the deterministic framework (3) has a forward and backward at $\Re _{0}=1.$ In Section “Stochastic analysis”, we use a stochastic Lyapunov candidate technique to develop the necessary requirements for an ergodic stationary distribution of effective solutions to the stochastic system (5) to arise and be distinct. Also, the unique global positive solution for every positive initial conditions is provided in detail. We accurately communicate the piecewise fractional differential equations with varying kernels of the stochastic system (5) in Section “Numerical simulations” under the same assumptions as stated in^37^, reflecting the strong extinction and persistence of the illness. In Section “Results and discussion”, simulation results are provided to certify our diagnostic results gained in Sections “Stochastic analysis and Numerical simulations”. This manuscript is concluded with a concise summary.

### Deterministic behaviour

Here, we demonstrate the mathematical and biophysical significance of our framework. Additionally, we will calculate the fundamental reproduction number and evaluate the steady state’s consistency.

#### Theorem 2.1

The closed set${\tilde{\Theta }}:=\Big ({\textbf{X}}_{{\textbf{h}}},{\textbf{P}}_{{\textbf{h}}}, {\textbf{Q}}_{{\textbf{h}}{\textbf{a}}},{\textbf{Q}}_{{\textbf{h}}{\textbf{s}}},{\textbf{R}}_{{\textbf{h}}} ,{\textbf{X}}_{{\textbf{r}}},{\textbf{P}}_{{\textbf{r}}},{\textbf{Q}}_{{\textbf{r}}}, {\textbf{G}}_{{\textbf{s}}},{\textbf{G}}_{{\textbf{a}}}\Big )$ is a positive invariant set for the proposed fractional-order system (7).

#### Proof

To prove that the system of Eq. (7) has a non-negative solution, the system of Eq. (7) implies

8 $$\begin{aligned} {\left\{ \begin{array}{ll} \,_{0}^{c}{\textbf{D}}_{{\textbf{t}}}^{\chi }{{\textbf{X}}_{{\textbf{h}}}}\big \vert _{{\textbf{X}}_{{\textbf{h}}}=0}=\Lambda _{1}\ge 0,\\ \,_{0}^{c}{\textbf{D}}_{{\textbf{t}}}^{\chi }{{\textbf{P}}_{{\textbf{h}}}}\big \vert _{{\textbf{P}}_{{\textbf{h}}}=0}=\Upsilon _{{\textbf{h}}}{\textbf{X}}_{{\textbf{h}}}\ge 0,\\ \,_{0}^{c}{\textbf{D}}_{{\textbf{t}}}^{\chi }{{\textbf{Q}}_{{\textbf{h}}{\textbf{a}}}}\big \vert _{{\textbf{Q}}_{{\textbf{h}}{\textbf{a}}}=0}=\mu \alpha _{1}{\textbf{P}}_{{\textbf{h}}}\ge 0,\\ \,_{0}^{c}{\textbf{D}}_{{\textbf{t}}}^{\chi }{{\textbf{Q}}_{{\textbf{h}}{\textbf{s}}}}\big \vert _{{\textbf{Q}}_{{\textbf{h}}{\textbf{s}}}=0}=(1-\mu )\alpha _{1}{\textbf{P}}_{{\textbf{h}}}\ge 0,\\ \,_{0}^{c}{\textbf{D}}_{{\textbf{t}}}^{\chi }{{\textbf{R}}_{{\textbf{h}}}}\big \vert _{{\textbf{R}}_{{\textbf{h}}}=0}=\phi _{1}{\textbf{P}}_{{\textbf{h}}}+\phi _{2}{\textbf{Q}}_{{\textbf{h}}{\textbf{s}}}\ge 0,\\ \,_{0}^{c}{\textbf{D}}_{{\textbf{t}}}^{\chi }{{\textbf{X}}_{{\textbf{r}}}}\big \vert _{{\textbf{X}}_{{\textbf{r}}}=0}=\Lambda _{2}-\Upsilon _{{\textbf{r}}}{\textbf{X}}_{{\textbf{r}}}-(\vartheta _{2}+\upsilon ){\textbf{R}}_{{\textbf{h}}}\ge 0,\\ \,_{0}^{c}{\textbf{D}}_{{\textbf{t}}}^{\chi }{{\textbf{P}}_{{\textbf{r}}}}\big \vert _{{\textbf{P}}_{{\textbf{r}}}=0}=\Upsilon _{{\textbf{r}}}{\textbf{X}}_{{\textbf{r}}}-(\alpha _{2}+\vartheta _{2}+\upsilon ){\textbf{P}}_{{\textbf{h}}}\ge 0,\\ \,_{0}^{c}{\textbf{D}}_{{\textbf{t}}}^{\chi }{{\textbf{Q}}_{{\textbf{r}}}}\big \vert _{{\textbf{Q}}_{{\textbf{r}}}=0}=\alpha _{2}{\textbf{P}}_{{\textbf{r}}}\ge 0,\\ \,_{0}^{c}{\textbf{D}}_{{\textbf{t}}}^{\chi }{{\textbf{G}}_{{\textbf{s}}}}\big \vert _{{\textbf{G}}_{{\textbf{s}}}=0}=\beta _{1}{\textbf{Q}}_{{\textbf{h}}{\textbf{a}}}+\beta _{2}{\textbf{Q}}_{{\textbf{h}}{\textbf{s}}}+\beta _{3}{\textbf{Q}}_{{\textbf{r}}}\ge 0,\\ \,_{0}^{c}{\textbf{D}}_{{\textbf{t}}}^{\chi }{{\textbf{G}}_{{\textbf{a}}}}\big \vert _{{\textbf{G}}_{{\textbf{a}}}=0}=\xi _{3}{\textbf{G}}_{{\textbf{s}}}\ge 0. \end{array}\right. } \end{aligned}$$

Thus, the fractional system (7) has non-negative solutions. In the end, from the first four equations of the fractional system (7), we obtain

$$\begin{aligned} \,_{0}^{c}{\textbf{D}}_{{\textbf{t}}}^{\chi }{\tilde{\Theta }}{} & {} \le \Lambda _{1}+\Upsilon _{{\textbf{h}}}{\textbf{X}}_{{\textbf{h}}} +\mu \alpha _{1}{\textbf{P}}_{{\textbf{h}}}+(1-\mu )\alpha _{1}{\textbf{P}}_{{\textbf{h}}} +\phi _{1}{\textbf{P}}_{{\textbf{h}}}+\phi _{2}{\textbf{Q}}_{{\textbf{h}}{\textbf{s}}}\nonumber \\{} & {} \le \Lambda _{1}-\vartheta _{1}{\textbf{N}}_{{\textbf{h}}}. \end{aligned}$$

Solving the above inequality, we obtain

$$\begin{aligned} {\tilde{\Theta }}({\textbf{t}})\le \big ({\tilde{\Theta }}(0)-\frac{\Lambda _{1}}{\vartheta _{1}}\big ) E_{\rho }(-\vartheta {\textbf{t}}^{\rho })+\frac{\Lambda _{1}}{\vartheta _{1}}, \end{aligned}$$

so by the asymptotic behavior of Mittag-Leffer function^33^, we obtain

$$\begin{aligned} {\tilde{\Theta }}({\textbf{t}})\le \frac{\Lambda _{1}}{\vartheta _{1}}\approx {\textbf{N}}_{{\textbf{h}}}, \end{aligned}$$

Taking the same steps for the sixth, seventh and eighth of system (8), we get ${\textbf{N}}_{{\textbf{r}}}=\frac{\Lambda _{2}}{\nu +\vartheta _{2}}.$ Analogously, we can deal the ninth and tenth compartment of (8), which yields ${\textbf{G}}_{{\textbf{s}}}\le \frac{(\beta _{1}+\beta _{2})\Lambda _{1}\vartheta _{1} +\beta _{3}\Lambda _{2}\vartheta _{1}}{\vartheta _{1}\vartheta _{2}(\xi _{2}+\xi _{3})}, {\textbf{G}}_{{\textbf{a}}}\le \frac{(\beta _{1}+\beta _{2})\Lambda _{1}\vartheta _{2}\xi _{3} +\xi _{3}\beta _{3}\Lambda _{2}\vartheta _{1}}{\vartheta _{1}\vartheta _{2}\xi _{2}(\xi _{2}+\xi _{3})}.$ Hence, the closed set ${\tilde{\Theta }}$ is a positive invariant region for the fractional-order Lassa fever model (7). $\square$

$\bullet$ We demonstrate that the solutions continue to stay positive and bounded in the suggested region, $\Pi$, under the assumption that all specifications are positive for time ${\textbf{t}}$. We shall examine the framework for Lassa fever ${\tilde{\Theta }}:=\Big ({\textbf{X}}_{{\textbf{h}}},{\textbf{P}}_{{\textbf{h}}},{\textbf{Q}}_{{\textbf{h}}{\textbf{a}}},{\textbf{Q}}_{{\textbf{h}}{\textbf{s}}},{\textbf{R}}_{{\textbf{h}}},{\textbf{X}}_{{\textbf{r}}},{\textbf{P}}_{{\textbf{r}}},{\textbf{Q}}_{{\textbf{r}}},{\textbf{G}}_{{\textbf{s}}},{\textbf{G}}_{{\textbf{a}}}\Big )$ spreads in the domain, which is as follows:

9 $$\begin{aligned} \Pi :=\Big \{{\tilde{\Theta }}\in \Re _{+}^{10}:{\textbf{N}}_{{\textbf{h}}}\le \frac{\Lambda _{1}}{\vartheta _{1}},{\textbf{N}}_{{\textbf{r}}}\le \frac{\Lambda _{2}}{\upsilon +\vartheta _{2}},{\textbf{G}}_{{\textbf{s}}}\le \frac{(\beta _{1}+\beta _{2})\Lambda _{1}\vartheta _{1}+\beta _{3}\Lambda _{2}\vartheta _{1}}{\vartheta _{1}\vartheta _{2}(\xi _{2}+\xi _{3})},{\textbf{G}}_{{\textbf{a}}}\le \frac{(\beta _{1}+\beta _{2})\Lambda _{1}\vartheta _{2}\xi _{3}+\xi _{3}\beta _{3}\Lambda _{2}\vartheta _{1}}{\vartheta _{1}\vartheta _{2}\xi _{2}(\xi _{2}+\xi _{3})}\Big \}. \end{aligned}$$

$\bullet$ The biological meaningful equilibria of fractional system (7) are disease-free equilibrium and endemic equilibrium, depending on infected classes in both the populations. To obtain the infection-free equilibrium, we set the fractional derivative $\,_{0}^{c}{\textbf{D}}_{{\textbf{t}}}^{\chi }{{\textbf{X}}_{{\textbf{h}}}},~\,_{0}^{c}{\textbf{D}}_{{\textbf{t}}}^{\chi }{{\textbf{P}}_{{\textbf{h}}}},\,_{0}^{c}{\textbf{D}}_{{\textbf{t}}}^{\chi }{{\textbf{Q}}_{{{\textbf{h}}}{{\textbf{a}}}}},\,_{0}^{c}{\textbf{D}}_{{\textbf{t}}}^{\chi }{{\textbf{Q}}_{{{\textbf{h}}}{{\textbf{s}}}}},\,_{0}^{c}{\textbf{D}}_{{\textbf{t}}}^{\chi }{{\textbf{R}}_{{\textbf{h}}}},\,_{0}^{c}{\textbf{D}}_{{\textbf{t}}}^{\chi }{{\textbf{X}}_{{\textbf{r}}}},\,_{0}^{c}{\textbf{D}}_{{\textbf{t}}}^{\chi }{{\textbf{P}}_{{\textbf{r}}}},\,_{0}^{c}{\textbf{D}}_{{\textbf{t}}}^{\chi }{{\textbf{Q}}_{{\textbf{r}}}},\,_{0}^{c}{\textbf{D}}_{{\textbf{t}}}^{\chi }{{\textbf{G}}_{{\textbf{s}}}},\,_{0}^{c}{\textbf{D}}_{{\textbf{t}}}^{\chi }{{\textbf{G}}_{{\textbf{a}}}}$ to zero of the fractional system (7) without infection, and get

$$\begin{aligned} {\mathcal {E}}_{0}=\Big (\frac{\Lambda _{1}}{\vartheta _{1}},0,0,0,0,\frac{\Lambda _{2}}{\upsilon +\vartheta _{2}},0,0,0,0\Big ). \end{aligned}$$

$\bullet$ To use the next generation matrix strategy^58^, the dominant eigenvalue of the matrix ${\textbf{F}}{\textbf{G}}^{-1}$ corresponds to the fundamental reproduction number $\Re _{0}$ of system (3). Therefore, we have

$$\begin{aligned}{\mathcal {F}}= \begin{pmatrix} \Upsilon _{{\textbf{h}}}{\textbf{X}}_{{\textbf{h}}}\\ 0\\ 0\\ \Upsilon _{{\textbf{r}}}{\textbf{X}}_{{\textbf{r}}}\\ 0\\ 0\\ 0 \end{pmatrix},~~~{\mathcal {V}}=\begin{pmatrix} (\vartheta _{1}+\alpha _{1}){\textbf{P}}_{{\textbf{h}}}\\ (\vartheta _{1}+\phi _{1}){\textbf{Q}}_{{\textbf{h}}{\textbf{a}}}-\mu \alpha _{1}{\textbf{P}}_{{\textbf{h}}}\\ (\vartheta _{1}+\phi _{2}+\delta ){\textbf{Q}}_{{\textbf{h}}{\textbf{s}}}-(1-\mu )\alpha _{1}{\textbf{P}}_{{\textbf{h}}}\\ (\vartheta _{2}+\alpha _{2}+\upsilon ){\textbf{P}}_{{\textbf{r}}}\\ (\upsilon +\vartheta _{2}){\textbf{Q}}_{{\textbf{r}}}-\alpha _{2}{\textbf{P}}_{{\textbf{r}}}\\ -\beta_{1}{\textbf{Q}}_{{\textbf{h}}{\textbf{a}}}-\beta _{2}{\textbf{Q}}_{{\textbf{h}}{\textbf{s}}}-\beta _{3}{\textbf{Q}}_{{\textbf{r}}}+(\xi _{2}+\xi _{3}){\textbf{G}}_{{\textbf{s}}}\\ \xi _{2}{\textbf{G}}_{{\textbf{a}}}-\xi _{3}{\textbf{G}}_{{\textbf{s}}} \end{pmatrix}. \end{aligned}$$

After, making use of the Jacobian of ${\textbf{F}}$ and ${\textbf{G}}$ reviewed at ${\mathcal {E}}_{0}$, we obtain the next generation matrix at disease-free equilibrium is

$$\begin{aligned} {\textbf{F}}{\textbf{G}}^{-1}=\begin{pmatrix} \tilde{b_{11}}&{}\tilde{b_{12}}&{}\tilde{b_{13}}&{}\tilde{b_{14}}&{}\tilde{b_{15}}&{}\tilde{b_{16}}&{}\tilde{b_{17}}\\ 0&{}0&{}0&{}0&{}0&{}0&{}0\\ 0&{}0&{}0&{}0&{}0&{}0&{}0\\ \tilde{b_{41}}&{}\tilde{b_{42}}&{}\tilde{b_{43}}&{}\tilde{b_{44}}&{}\tilde{b_{45}}&{}\tilde{b_{46}}&{}\tilde{b_{47}}\\ 0&{}0&{}0&{}0&{}0&{}0&{}0\\ 0&{}0&{}0&{}0&{}0&{}0&{}0\\ 0&{}0&{}0&{}0&{}0&{}0&{}0 \end{pmatrix}, \end{aligned}$$

where

$$\begin{aligned}\tilde{b_{\iota {\textbf{k}}}}= {\left\{ \begin{array}{ll} \frac{\gamma _{{\textbf{h}}}\big (\Phi _{{\textbf{v}}}\xi _{2}(\xi _{2}+\xi _{3})\vartheta _{1}(\rho _{2}(\delta +\phi _{2}+\vartheta _{1})\mu \alpha _{1})+(1-\mu )\rho _{1}(\vartheta _{1}+\phi _{1})\alpha _{1}\big )}{\Phi _{{\textbf{v}}}\xi _{2}(\xi _{2}+\xi _{3})\vartheta _{1}(\phi _{1}+\vartheta _{1})(\delta +\phi _{2}+\vartheta _{1})(\vartheta _{1}+\alpha _{1})}\\ + \frac{\gamma _{{\textbf{h}}}\big (\Lambda _{1}(\rho _{3}\xi _{2}+\rho _{4}\xi _{3})(\delta +\phi _{2}+\vartheta _{1})\mu \alpha _{1}\beta _{1}+(1-\mu )\phi _{1}(\vartheta _{1}+\phi _{1})\alpha _{1}\beta _{2}\big )}{\Phi _{{\textbf{v}}}\xi _{2}(\xi _{2}+\xi _{3})\vartheta _{1}(\phi _{1}+\vartheta _{1})(\delta +\phi _{2}+\vartheta _{1})(\vartheta _{1}+\alpha _{1})},~~\iota =1,~{\textbf{k}}=1,\\ \frac{\gamma _{{\textbf{h}}}\rho _{2}}{\vartheta _{1}+\phi _{1}}+\frac{\Lambda _{1}\gamma _{{\textbf{h}}}\rho _{3}\beta _{1}}{\Phi _{{\textbf{v}}}(\xi _{2}+\xi _{3})(\vartheta _{1}+\phi _{1})\vartheta _{1}}+\frac{\Lambda _{1}\gamma _{{\textbf{h}}}\rho _{4}\xi _{3}\beta _{2}}{\Phi _{{\textbf{v}}}\vartheta _{1}\xi _{2}(\xi _{2}+\xi _{3})(\phi _{1}+\vartheta _{1})},~~\iota =1,~{\textbf{k}}=2,\\ \frac{\gamma _{{\textbf{h}}}\rho _{1}}{\vartheta _{1}+\phi _{2}+\delta }+\frac{\Lambda _{1}\gamma _{{\textbf{h}}\rho _{3}\beta _{2}}}{\Phi _{{\textbf{v}}}(\xi _{2}+\xi _{3})(\vartheta _{1}+\phi _{1})\vartheta _{1}}+\frac{\Lambda _{1}\gamma _{{\textbf{h}}}\rho _{4}\xi _{3}\beta _{2}}{\Phi _{{\textbf{v}}}\vartheta _{1}\xi _{2}(\xi _{2}+\xi _{3})(\phi _{2}+\delta +\vartheta _{1})},~~\iota =1,~{\textbf{k}}=3,\\ \frac{\Lambda _{1}\gamma _{{\textbf{h}}}(\Phi _{{\textbf{v}}}\xi _{2}(\xi _{2}+\xi _{3})\vartheta _{2}+\Lambda _{2}\beta _{3}(\rho _{3}\xi _{2}+\rho _{4}\xi _{3})\alpha _{2})}{\Phi _{{\textbf{v}}}\vartheta _{1}\xi _{2}\Lambda _{2}(\xi _{2}+\xi _{3})(\upsilon +\alpha _{2})(\vartheta _{2}+\upsilon +\alpha _{2})},~~\iota =1,~{\textbf{k}}=4,\\ \frac{\Lambda _{1}\gamma _{{\textbf{h}}}\vartheta _{2}}{\vartheta _{1}\Lambda _{2}(\upsilon +\alpha _{2})}+\frac{\Lambda _{1}\gamma _{{\textbf{h}}}\rho _{3}\beta _{3}}{\Phi _{{\textbf{v}}}(\xi _{2}+\xi _{3})\vartheta _{1}(\upsilon +\alpha _{2})}+\frac{\Lambda _{1}\gamma _{{\textbf{h}}}\rho _{4}\xi _{3}\beta _{3}}{\Phi _{{\textbf{v}}}\xi _{2}(\xi _{2}+\xi _{3})\vartheta _{1}(\upsilon +\alpha _{2})},~~\iota =1,~{\textbf{k}}=5,\\ \frac{\Lambda _{1}\gamma _{{\textbf{h}}}\rho _{3}}{\Phi _{{\textbf{v}}}\vartheta _{1}(\xi _{2}+\xi _{3})}+\frac{\Lambda _{1}\gamma _{{\textbf{h}}}\rho _{4}\xi _{3}}{\Phi _{{\textbf{v}}}\xi _{2}\vartheta _{1}(\xi _{2}+\xi _{3})},~~\iota =1,~{\textbf{k}}=6,\\ \frac{\Lambda _{1}\gamma _{{\textbf{h}}}\rho _{4}}{\Phi _{{\textbf{v}}}\xi _{2}\vartheta _{1}},~~\iota =1,~{\textbf{k}}=7,\\ \frac{\Lambda _{2}\gamma _{{\textbf{r}}}\phi _{1}\big ((\delta +\phi _{2}+\vartheta _{1})\mu \alpha _{1}\beta _{1}+(1-\mu )(\vartheta _{1}+\phi _{1})\alpha _{1}\beta _{2}\big )}{\Phi _{{\textbf{v}}}\vartheta _{1}(\xi _{2}+\xi _{3})(\vartheta _{1}+\phi _{1})(\vartheta _{1}+\delta +\phi _{2})(\vartheta _{1}+\alpha _{1})},~~\iota =4,~{\textbf{k}}=1,\\ \frac{\Lambda _{2}\gamma _{{\textbf{r}}}\phi _{1}\beta _{1}}{\Phi _{{\textbf{v}}}\vartheta _{2}(\xi _{2}+\xi _{3})(\vartheta _{1}+\phi _{1})},~~\iota =4,~{\textbf{k}}=2,\\ \frac{\Lambda _{2}\gamma _{{\textbf{r}}}\phi _{1}\beta _{2}}{\Phi _{{\textbf{v}}}\vartheta _{2}(\xi _{2}+\xi _{3})(\delta +\vartheta _{1}+\phi _{2})},~~\iota =4,~{\textbf{k}}=3,\\ \frac{\gamma _{{\textbf{r}}}\big (\Phi _{{\textbf{v}}}(\xi _{2}+\xi _{3})\vartheta _{2}+\phi _{1}\Lambda _{2}\beta _{3}\big )\alpha _{2}}{\Phi _{{\textbf{v}}}\vartheta _{2}(\xi _{2}+\xi _{3})(\upsilon +\vartheta _{2}+\alpha _{2})(\upsilon +\alpha _{2})},~~\iota =4,~{\textbf{k}}=4,\\ \frac{\gamma _{{\textbf{r}}}}{\upsilon +\alpha _{2}}+\frac{\Lambda _{2}\gamma _{{\textbf{r}}}\phi _{1}\beta _{3}}{\Phi _{{\textbf{v}}}\vartheta _{2}(\xi _{2}+\xi _{3})(\upsilon +\alpha 
_{2})},~~\iota =4,~{\textbf{k}}=5,\\ \frac{\Lambda _{2}\gamma _{{\textbf{r}}}\phi _{1}}{\Phi _{{\textbf{v}}}\vartheta _{2}(\xi _{2}+\xi _{3})},~~\iota =4,~{\textbf{k}}=6. \end{array}\right. } \end{aligned}$$

The fundamental reproductive number $\Re _{0}$ can be formulated as

10 $$\begin{aligned} \Re _{0}=\frac{(\tilde{b_{11}}+\tilde{b_{44}})+\sqrt{(\tilde{b_{11}}-\tilde{b_{44}})^{2}+4\tilde{b_{14}}\tilde{b_{41}}}}{2}, \end{aligned}$$

which is employed to establish whether the ailment manifests itself or not.

Next, we will illustrate the persistence of infection in the fractional-order system. It describes the level of endemicity of infection in the system. Biologically speaking, the infection persists in the system if the level of infected fraction stays at a higher level for ${\textbf{t}}$ large enough.

(i)  When $\Re _{0}\le 1,$ then fractional-order model (7) has a steady state ${\mathcal {E}}_{0}=\Big (\frac{\Lambda _{1}}{\vartheta _{1}},0,0,0,0,\frac{\Lambda _{2}}{\upsilon +\vartheta _{2}},0,0,0,0\Big )$ and is globally asymptotically stable in the positive invariant set $\Pi .$

(ii) When $\Re _{0}>1,$ then ${\mathcal {E}}_{0}$ is unstable and the fractional-order system (7) is uniformly persistent. Thus, there is a unique globally asymptotically stable endemic equilibrium ${\mathcal {E}}_{1}=\big ({\textbf{X}}_{{\textbf{h}}}^{*},{\textbf{P}}_{{\textbf{h}}}^{*},{\textbf{Q}}_{{\textbf{h}}{\textbf{a}}}^{*},{\textbf{Q}}_{{\textbf{h}}{\textbf{s}}}^{*},{\textbf{R}}_{{\textbf{h}}}^{*},{\textbf{X}}_{{\textbf{r}}}^{*},{\textbf{P}}_{{\textbf{r}}}^{*},{\textbf{Q}}_{{\textbf{r}}}^{*},{\textbf{G}}_{{\textbf{s}}}^{*},{\textbf{G}}_{{\textbf{a}}}^{*}\big )$ in the interior of $\Pi ,$ where

11 $$\begin{aligned}{} & {} {\textbf{X}}_{{\textbf{h}}}^{*}=\frac{\Lambda _{1}}{\Upsilon _{{\textbf{h}}}^{*}+\vartheta _{1}},~~~{\textbf{P}}_{{\textbf{h}}}^{*}=\frac{\Lambda _{1}\Upsilon _{{\textbf{h}}}^{*}}{(\vartheta _{1}+\alpha _{1})(\Upsilon _{{\textbf{h}}}^{*}+\vartheta _{1})},~~~{\textbf{Q}}_{{\textbf{h}}{\textbf{a}}}^{*}=\frac{\Lambda _{1}\mu \alpha _{1}\Upsilon _{{\textbf{h}}}^{*}}{(\vartheta _{1}+\phi _{1})(\Upsilon _{{\textbf{h}}}^{*}+\vartheta _{1})(\vartheta _{1}+\alpha _{1})},\nonumber \\{} & {} {\textbf{Q}}_{{\textbf{h}}{\textbf{s}}}^{*}=\frac{(1-\mu )\Lambda _{1}\alpha _{1}\Upsilon _{{\textbf{h}}}^{*}}{(\vartheta _{1}+\phi _{2}+\delta )(\alpha _{1}+\vartheta _{1})(\vartheta _{1}+\alpha _{1})(\vartheta _{1}+\Upsilon _{{\textbf{h}}})},\nonumber \\{} & {} {\textbf{R}}_{{\textbf{h}}}^{*}=\frac{\mu \Lambda _{1}\alpha _{1}\phi _{1}\Upsilon _{{\textbf{h}}}^{*}}{\vartheta _{1}(\vartheta _{1}+\phi _{1})(\alpha _{1}+\vartheta _{1})(\vartheta _{1}+\Upsilon _{{\textbf{h}}})}+\frac{\phi _{2}\Lambda _{1}\alpha _{1}(1-\mu )\Upsilon _{{\textbf{h}}}^{*}}{\vartheta _{1}(\alpha _{1}+\vartheta _{1})(\Upsilon _{{\textbf{h}}}^{*}+\vartheta _{1})(\vartheta _{1}+\delta +\phi _{2})},\nonumber \\{} & {} {\textbf{X}}_{{\textbf{r}}}^{*}=\frac{\Lambda _{2}}{\Upsilon _{{\textbf{r}}}^{*}+\vartheta _{2}+\upsilon },~~~{\textbf{P}}_{{\textbf{r}}}^{*}=\frac{\Lambda _{2}\Upsilon _{{\textbf{r}}}^{*}}{(\vartheta _{2}+\alpha _{2}+\upsilon )(\Upsilon _{{\textbf{r}}}^{*}+\vartheta _{2}+\upsilon )},~~~{\textbf{Q}}_{{\textbf{r}}}^{*}=\frac{\Lambda _{2}\alpha _{2}\Upsilon _{{\textbf{r}}}^{*}}{(\vartheta _{2}+\upsilon )(\Upsilon _{{\textbf{r}}}^{*}+\vartheta _{2}+\upsilon )(\vartheta _{2}+\alpha _{2}+\upsilon )},\nonumber \\{} & {} {\textbf{G}}_{{\textbf{s}}}^{*}=\frac{\beta _{1}\mu \alpha _{1}\Lambda _{1}\Upsilon _{{\textbf{h}}}^{*}}{(\xi _{2}+\xi _{3})(\vartheta _{1}+\phi _{1})(\vartheta _{1}+\alpha _{1})(\vartheta _{1}+\Upsilon _{{\textbf{h}}}^{*})}+\frac{(1-\mu )\beta _{2}\alpha _{1}\Lambda _{1}\Upsilon _{{\textbf{h}}}^{*}}{(\xi _{2}+\xi _{3})(\vartheta _{1}+\alpha _{1})(\vartheta _{1}+\phi _{2}+\delta )(\vartheta _{1}+\Upsilon _{{\textbf{h}}}^{*})}\nonumber \\{} & {} \qquad \quad +\frac{\beta _{3}\alpha _{2}\Lambda _{2}\Upsilon _{{\textbf{r}}}^{*}}{(\xi _{2}+\xi _{3})(\upsilon +\vartheta _{2})(\upsilon +\vartheta _{2}+\alpha _{2})(\upsilon +\vartheta _{2}+\Upsilon _{{\textbf{r}}}^{*})},\nonumber \\{} & {} {\textbf{G}}_{{\textbf{a}}}^{*}=\frac{\xi _{3}\beta _{1}\mu \alpha _{1}\Lambda _{1}}{\xi _{2}(\xi _{2}+\xi _{3})(\vartheta _{1}+\phi _{1})(\vartheta _{1}+\alpha _{1})(\vartheta _{1}+\Upsilon _{{\textbf{h}}}^{*})}+\frac{\xi _{3}\beta _{2}\alpha _{1}\Lambda _{1}\Upsilon _{{\textbf{h}}}^{*}(1-\mu )}{\xi _{2}(\xi _{2}+\xi _{3})(\vartheta _{1}+\alpha _{1})(\vartheta _{1}+\phi _{1}+\delta )(\vartheta _{1}+\Upsilon _{{\textbf{h}}}^{*})}\nonumber \\{} & {} \qquad \quad +\frac{\xi _{3}\beta _{3}\alpha _{2}\Lambda _{2}\Upsilon _{{\textbf{r}}}^{*}}{\xi _{2}(\xi _{2}+\xi _{3})(\vartheta _{2}+\upsilon )(\vartheta _{2}+\alpha _{2}+\upsilon )(\vartheta _{2}+\upsilon +\Upsilon _{{\textbf{r}}}^{*})}, \end{aligned}$$

and

$$\begin{aligned}{} & {} \Upsilon _{{\textbf{h}}}^{*}=\gamma _{{\textbf{h}}}\Big (\frac{{\textbf{Q}}_{{\textbf{r}}}^{*}}{{\textbf{N}}_{{\textbf{r}}}^{*}}+\frac{\rho _{1}{\textbf{Q}}_{{\textbf{h}}{\textbf{s}}}^{*}}{{\textbf{N}}_{{\textbf{h}}}^{*}}+\frac{\rho _{2}{\textbf{Q}}_{{\textbf{h}}{\textbf{a}}}^{*}}{{\textbf{N}}_{{\textbf{h}}}^{*}}+\frac{\rho _{3}{\textbf{G}}_{{\textbf{s}}}^{*}}{\Phi _{{\textbf{v}}}^{*}}+\frac{\rho _{4}{\textbf{G}}_{{\textbf{a}}}^{*}}{\Phi _{{\textbf{v}}}^{*}}\Big ),\nonumber \\{} & {} \Upsilon _{{\textbf{r}}}^{*}=\gamma _{{\textbf{r}}}\Big (\frac{{\textbf{Q}}_{{\textbf{r}}}^{*}}{{\textbf{N}}_{{\textbf{r}}}^{*}}+\frac{\phi _{1}{\textbf{G}}_{{\textbf{s}}}^{*}}{\Phi _{{\textbf{v}}}^{*}}\Big ).\end{aligned}$$

#### Theorem 2.2

**(i)**  When $\frac{\gamma _{{\textbf{h}}}\vartheta _{2}{\textbf{w}}_{1}}{\Lambda _{2}}\mu _{2}<\frac{\gamma _{{\textbf{h}}\Lambda _{1}\vartheta _{2}^{2}}}{\Lambda _{2}^{2}\vartheta _{1}}\mu _{2}{\textbf{w}}_{6}{\textbf{w}}_{8}$ with $b_{1}>0,$ then the model (3) will endure a forward bifurcation at $\Re _{0}=1.$ (ii)  When $\gamma _{w_{\prime }\mu }<\frac{\gamma _{{\textbf{h}}}\vartheta _{2}}{\Lambda _{2}^{2}}\mu _{2}{\textbf{w}}_{1}^{2}-\frac{\gamma _{{\textbf{h}}}\Lambda _{1}\vartheta _{2}^{2}}{\Lambda _{2}^{2}\vartheta _{1}}\mu _{2}{\textbf{w}}_{2}{\textbf{w}}_{6}$ with $b_{1}>0,$ then the model (3) will endure a backward bifurcation at $\Re _{0}=1.$

#### Proof

Suppose ${\textbf{y}}_{{\textbf{m}}}=({\textbf{y}}_{1},{\textbf{y}}_{2},{\textbf{y}}_{3},{\textbf{y}}_{4},{\textbf{y}}_{5},{\textbf{y}}_{6},{\textbf{y}}_{7},{\textbf{y}}_{8},{\textbf{y}}_{9},{\textbf{y}}_{10})^{{\mathbb {T}}}=\big ({\textbf{X}}_{{\textbf{h}}},{\textbf{P}}_{{\textbf{h}}},{\textbf{Q}}_{{\textbf{h}}{\textbf{a}}},{\textbf{Q}}_{{\textbf{h}}{\textbf{s}}},{\textbf{R}}_{{\textbf{h}}},{\textbf{X}}_{{\textbf{r}}},{\textbf{P}}_{{\textbf{r}}},{\textbf{Q}}_{{\textbf{r}}},{\textbf{G}}_{{\textbf{s}}}\big )^{{\mathbb {T}}}.$ Then, framework (3) can be composed as ${\dot{y}}_{{\textbf{m}}}=g_{1}(x_{1})$ as shown in:

12 $$\begin{aligned} {\left\{ \begin{array}{ll} \dot{{\textbf{y}}_{1}}=g_{1}=\Lambda _{1}-\Upsilon _{{\textbf{h}}}{\textbf{y}}_{1}-\vartheta _{1}{\textbf{y}}_{1},\\ \dot{{\textbf{y}}_{2}}=g_{2}=\Upsilon _{{\textbf{h}}}{\textbf{y}}_{1}-(\alpha _{1}+\vartheta _{1}){\textbf{y}}_{2},\\ \dot{{\textbf{y}}_{3}}=g_{3}=\mu \alpha _{1}{\textbf{y}}_{2}-(\phi _{1}+\vartheta _{1}){\textbf{y}}_{3},\\ \dot{{\textbf{y}}_{4}}=g_{4}=(1-\mu )\alpha _{1}{\textbf{y}}_{2}-(\delta +\phi _{2}+\vartheta _{1}){\textbf{y}}_{3},\\ \dot{{\textbf{y}}_{5}}=g_{5}=\phi _{1}{\textbf{y}}_{3}+\phi _{2}{\textbf{y}}_{4}-\vartheta _{1}{\textbf{y}}_{5},\\ \dot{{\textbf{y}}_{6}}=g_{6}=\Lambda _{2}-\Upsilon _{{\textbf{r}}}{\textbf{y}}_{6}-(\vartheta _{2}+\upsilon ){\textbf{y}}_{6},\\ \dot{{\textbf{y}}_{7}}=g_{7}=\Upsilon _{{\textbf{r}}}{\textbf{y}}_{6}-(\alpha _{2}+\vartheta _{2}+\upsilon ){\textbf{y}}_{7},\\ \dot{{\textbf{y}}_{8}}=g_{8}=\alpha _{2}{\textbf{y}}_{7}-(\upsilon +\vartheta _{2}){\textbf{y}}_{8},\\ \dot{{\textbf{y}}_{9}}=g_{9}=\beta _{1}{\textbf{y}}_{3}+\beta _{2}{\textbf{y}}_{4} +\beta _{3}{\textbf{y}}_{8}-(\xi _{2}+\xi _{3}){\textbf{y}}_{9},\\ \dot{{\textbf{y}}_{10}}=g_{10}=\xi _{3}{\textbf{y}}_{9}-\xi _{2}{\textbf{y}}_{10}, \\ \end{array}\right. } \end{aligned}$$

where $$\Upsilon _{{\textbf{h}}}=\frac{\gamma _{{\textbf{h}}}}{{\textbf{y}}_{6} +{\textbf{y}}_{7}+{\textbf{y}}_{8}}+\frac{\gamma _{{\textbf{h}}\rho _{1}}{\textbf{y}}_{4}}{{\textbf{y}}_{1}+{\textbf{y}}_{2}+{\textbf{y}}_{3}+{\textbf{y}}_{4}+{\textbf{y}}_{5}} +\frac{\gamma _{{\textbf{h}}\rho _{2}}{\textbf{y}}_{3}}{{\textbf{y}}_{1}+{\textbf{y}}_{2} +{\textbf{y}}_{3}+{\textbf{y}}_{4}+{\textbf{y}}_{5}}+\frac{\gamma _{{\textbf{h}}}\rho _{3} {\textbf{y}}_{9}}{\Phi _{{\textbf{v}}}}+\frac{\gamma _{{\textbf{h}}}\rho _{4}{\textbf{y}}_{10}}{\Phi _{{\textbf{v}}}}$$ and $$\Upsilon _{{\textbf{r}}}=\gamma _{{\textbf{r}}}\Big (\frac{\phi _{1} {\textbf{y}}_{9}}{\Phi _{{\textbf{v}}}}+\frac{{\textbf{y}}_{8}}{{\textbf{y}}_{6}+{\textbf{y}}_{7}+{\textbf{y}}_{8}}\Big ).$$

By adjusting $\Re _{0}=1$, we select $\gamma _{{\textbf{h}}}$ as the bifurcation deviates. Let $\gamma _{{\textbf{r}}}\propto \gamma _{{\textbf{h}}}$, which suggests that $\gamma _{{\textbf{r}}}=\tau \gamma _{{\textbf{h}}}$ for such ${\textbf{t}}>0$. Following that we obtain from the value of $\Re _{0}$

13 $$\begin{aligned} \gamma _{{\textbf{h}}}=\gamma _{{\textbf{h}}}^{*}=\frac{2}{\gamma _{{\textbf{h}}^{\prime }}^{*}+\gamma _{H_{4}^{\prime }}^{*}+\sqrt{(\gamma _{{\textbf{h}}^{\prime }}^{*}-\gamma _{H_{4}^{\prime }}^{*})^{2}+4\gamma _{H_{2}^{\prime }}^{*}\gamma _{H_{3}^{\prime }}^{*}}}, \end{aligned}$$

where

14 $$\begin{aligned}{} & {} \gamma _{{\textbf{h}}^{\prime }}^{*}=\frac{\vartheta _{1}\Phi _{{\textbf{v}}}\xi _{2}(\xi _{2}+\xi _{3})\big (\rho _{2}\mu \alpha _{1}(\vartheta _{1}+\phi _{2}+\delta )-\rho _{1}\alpha _{1}(\mu -1)(\vartheta _{1}+\phi _{1})\big )}{\vartheta _{1}\Phi _{{\textbf{v}}}\xi _{2}(\xi _{2}+\xi _{3})(\vartheta _{1}+\phi _{1})(\delta +\phi _{2}+\vartheta _{1})(\vartheta _{1}+\alpha _{1})}\nonumber \\{} & {} \qquad \quad +\frac{\Lambda _{1}(\rho _{3}\xi _{2}+\rho _{4}\xi _{3})\big (\mu \alpha _{1}\beta _{1}(\vartheta _{1}+\phi _{1}+\delta )-(\mu -1)(\vartheta _{1}+\phi _{1})\alpha _{1}\beta _{2}\big )}{\vartheta _{1}\Phi _{{\textbf{v}}}\xi _{2}(\xi _{2}+\xi _{3})(\vartheta _{1}+\phi _{1})(\delta +\phi _{2}+\vartheta _{1})(\vartheta _{1}+\alpha _{1})},\nonumber \\{} & {} \gamma _{H_{3}^{\prime }}^{*}=\frac{\Lambda _{1}\alpha _{2}\big (\Phi _{{\textbf{v}}}\xi _{2})(\xi _{2}+\xi _{3})\vartheta _{2}+\Lambda _{2}\beta _{3}(\rho _{3}\xi _{2}+\rho _{4}\xi _{3})\big )}{\Phi _{{\textbf{v}}}\Lambda _{2}\vartheta _{1}\xi _{2}(\xi _{2}+\xi _{3})(\upsilon +\alpha _{2})(\upsilon +\vartheta _{2}+\alpha _{2})},\nonumber \\{} & {} \gamma _{H_{2}^{\prime }}^{*}=\frac{\Lambda _{2}\tau \phi _{1}\big (\mu \alpha _{1}\beta _{1}(\vartheta _{1}+\delta +\phi _{1})-\alpha _{1}\beta _{2}(\mu -1)(\vartheta _{1}+\phi _{1})\big )}{\vartheta _{2}\Phi _{{\textbf{v}}}(\xi _{2}+\xi _{3})(\vartheta _{1}+\phi _{1})(\vartheta _{1}+\phi _{2}+\delta )(\vartheta _{1}+\alpha _{1})},\nonumber \\{} & {} \gamma _{H_{4}^{\prime }}^{*}=\frac{\tau \alpha _{2}\big (\Phi _{{\textbf{v}}}\vartheta _{2}(\xi _{2}+\xi _{3})+\Lambda _{2}\phi _{1}\beta _{3}\big )}{\Phi _{{\textbf{v}}}\vartheta _{2}(\xi _{2}+\xi _{3})(\upsilon +\alpha _{2})(\upsilon +\vartheta _{2}+\alpha _{2})}. \end{aligned}$$

Now, the Jacobian matrix of model (3) is provided as assessed at the DFE ${\mathcal {E}}_{0}$ in view of the bifurcation criterion $\gamma _{{\textbf{h}}}^{*}$ presented as

15 $$\begin{aligned} {\mathcal {J}}_{{\mathcal {E}}_{0}}=\begin{pmatrix} -\vartheta _{1}&{}0&{}-\gamma _{{\textbf{h}}}^{*}\rho _{2}&{}-\gamma _{{\textbf{h}}}^{*}\rho _{1}&{}0&{}0&{}0&{}-\frac{\gamma _{{\textbf{h}}}^{*}\vartheta _{2}\Lambda _{1}}{\Lambda _{2}\vartheta _{1}}&{}-\frac{\gamma _{{\textbf{h}}}^{*}\rho _{3}\Lambda _{1}}{\Phi _{{\textbf{v}}}\vartheta _{1}}&{}-\frac{\gamma _{{\textbf{h}}}^{*}\rho _{4}\Lambda _{1}}{\Phi _{{\textbf{v}}}\vartheta _{1}}\\ 0&{}{\textbf{k}}_{22}&{}\gamma _{{\textbf{h}}}^{*}\rho _{2}&{}*\gamma _{{\textbf{h}}}^{*}\rho _{1}&{}0&{}0&{}0&{}\frac{\gamma _{{\textbf{h}}}^{*}\vartheta _{2}\Lambda _{1}}{\Lambda _{2}\vartheta _{1}}&{}\frac{\gamma _{{\textbf{h}}}^{*}\rho _{3}\Lambda _{1}}{\Phi _{{\textbf{v}}}\vartheta _{1}}&{}\frac{\gamma _{{\textbf{h}}}^{*}\rho _{4}\Lambda _{1}}{\Phi _{{\textbf{v}}}\vartheta _{1}}\\ 0&{}\mu \alpha _{1}&{}{\textbf{k}}_{33}&{}0&{}0&{}0&{}0&{}0&{}0&{}0\\ 0&{}(1-\mu )\alpha _{1}&{}0&{}{\textbf{k}}_{44}&{}0&{}0&{}0&{}0&{}0&{}0\\ 0&{}0&{}\phi _{1}&{}\phi _{2}&{}-\vartheta _{1}&{}0&{}0&{}0&{}0&{}0\\ 0&{}0&{}0&{}0&{}0&{}-(\upsilon +\vartheta _{2})&{}0&{}-\tau \gamma _{{\textbf{h}}}^{*}&{}-\frac{\tau \gamma _{{\textbf{h}}}^{*}\phi _{1}\vartheta _{2}}{\vartheta _{2}\Phi _{{\textbf{v}}}}&{}0\\ 0&{}0&{}0&{}0&{}0&{}0&{}{\textbf{k}}_{77}&{}\tau \gamma _{{\textbf{h}}}^{*}&{}\frac{\tau \gamma _{{\textbf{h}}}^{*}\phi _{1}\vartheta _{2}}{\vartheta _{2}\Phi _{{\textbf{v}}}}&{}0\\ 0&{}0&{}0&{}0&{}0&{}0&{}\alpha _{2}&{}{\textbf{k}}_{88}&{}0&{}0\\ 0&{}0&{}\beta _{1}&{}\beta _{2}&{}0&{}0&{}0&{}\beta _{3}&{}-(\xi _{2}+\xi _{3})&{}0\\ 0&{}0&{}0&{}0&{}0&{}0&{}0&{}0&{}\xi _{3}&{}-\xi _{2} \end{pmatrix}, \end{aligned}$$

where $${\textbf{k}}_{22}=-(\vartheta _{1}+\alpha _{1}),~~{\textbf{k}}_{33}=-(\vartheta _{1}+\phi _{1}),~~ {\textbf{k}}_{44}=-(\vartheta _{1}+\phi _{2}+\delta ),~~{\textbf{k}}_{77}=-(\vartheta _{2}+\upsilon +\alpha _{2}),~~{\textbf{k}}=-(\upsilon +\vartheta _{2}).$$

The zero eigenvalue is connected with an appropriate eigenvector $\bar{w}=({\textbf{w}}_{1},{\textbf{w}}_{2},{\textbf{w}}_{3},{\textbf{w}}_{4}, {\textbf{w}}_{5},{\textbf{w}}_{6},{\textbf{w}}_{7},{\textbf{w}}_{8},{\textbf{w}}_{9},{\textbf{w}}_{10})^{{\mathbb {T}}}.$

It is constructed using the respective formulae:

16 $$\begin{aligned}{} & {} -\gamma _{{\textbf{h}}}^{*}\rho _{2}{\textbf{w}}_{3}-\vartheta _{1}{\textbf{w}}_{1}-\gamma _{{\textbf{h}}}^{*}\rho _{1}{\textbf{w}}_{4}-\frac{\vartheta _{2}\Lambda _{1}\gamma _{{\textbf{h}}}^{*}{\textbf{w}}_{8}}{\vartheta _{1}\Lambda _{2}}-\frac{\Lambda _{1}\gamma _{{\textbf{h}}}^{*}\rho _{3}{\textbf{w}}_{9}}{\vartheta _{1}\Phi _{{\textbf{v}}}}-\frac{\Lambda _{1}\gamma _{{\textbf{h}}}^{*}\rho _{4}{\textbf{w}}_{10}}{\vartheta _{1}\Phi _{{\textbf{v}}}}=0,\nonumber \\ {}{} & {} \frac{\vartheta _{2}\Lambda _{1}\gamma _{{\textbf{h}}}^{*}{\textbf{w}}_{8}}{\vartheta _{1}\Lambda _{2}}+\frac{\rho _{3}\Lambda _{1}\gamma _{{\textbf{h}}}^{*}{\textbf{w}}_{9}}{\vartheta _{1}\Phi _{{\textbf{v}}}}+\frac{\rho _{4}\Lambda _{1}\gamma _{{\textbf{h}}}^{*}{\textbf{w}}_{10}}{\vartheta _{1}\Phi _{{\textbf{v}}}}-(\vartheta _{1}+\alpha _{1}){\textbf{w}}_{2}+\gamma _{{\textbf{h}}}\rho _{2}{\textbf{w}}_{3}+\gamma _{{\textbf{h}}}^{*}\rho _{1}W_{4}=0,\nonumber \\ {}{} & {} \mu \alpha _{1} {\textbf{w}}_{2}-(\phi _{1}+\vartheta _{1}){\textbf{w}}_{3}=0,\nonumber \\ {}{} & {} (1-\mu )\alpha _{1}{\textbf{w}}_{2}-(\delta +\phi _{2}+\vartheta _{1}){\textbf{w}}_{4}=0,\nonumber \\ {}{} & {} -\vartheta _{1}{\textbf{w}}_{5}+\phi _{1}{\textbf{w}}_{3}+\phi _{2}{\textbf{w}}_{4}=0,\nonumber \\ {}{} & {} -(\upsilon +\vartheta _{2}){\textbf{w}}_{6}-\tau \gamma _{{\textbf{h}}}^{*}{\textbf{w}}_{8}-\frac{\tau \gamma _{{\textbf{h}}}^{*}\phi _{1}\Lambda _{2}}{\vartheta _{2}\Phi _{{\textbf{v}}}}{\textbf{w}}_{9}=0,\nonumber \\ {}{} & {} \tau \gamma _{{\textbf{h}}}^{*}{\textbf{w}}_{8}-(\alpha _{2}+\upsilon +\vartheta _{2}){\textbf{w}}_{7}+\frac{\tau \gamma _{{\textbf{h}}}^{*}\phi _{1}\Lambda _{2}{\textbf{w}}_{9}}{\vartheta _{2}\Phi _{{\textbf{v}}}}=0,\nonumber \\ {}{} & {} \alpha _{2}{\textbf{w}}_{7}-(\upsilon +\vartheta _{2}){\textbf{w}}_{8}=0,\nonumber \\ {}{} & {} \beta _{1}{\textbf{w}}_{3}+\beta _{2}{\textbf{w}}_{4}+\beta _{3}{\textbf{w}}_{8}-(\xi _{2}+\xi _{3}{\textbf{w}}_{9})=0,\nonumber \\ {}{} & {} \xi _{3}{\textbf{w}}_{9}-\xi _{2}{\textbf{w}}_{10}=0. \end{aligned}$$

The findings for (16) yields

$$\begin{aligned}{} & {} {\textbf{w}}_{1}=\frac{\gamma _{{\textbf{h}}}^{*}\rho _{2}{\textbf{w}}_{3}}{\vartheta _{1}-\gamma _{{\textbf{h}}}^{*}\rho _{1}{\textbf{w}}_{4}}-\frac{\gamma _{{\textbf{h}}}^{*}\vartheta _{2}\Lambda _{1} {\textbf{w}}_{8}}{\vartheta _{1}^{2}\Lambda _{2}}-\frac{\gamma _{{\textbf{h}}}^{*}\rho _{3}\Lambda _{1} {\textbf{w}}_{9}-\gamma _{{\textbf{h}}}^{*}\rho _{4}\Lambda _{1} {\textbf{w}}_{10}}{\vartheta _{1}^{2}\Phi _{{\textbf{v}}}},\nonumber \\ {}{} & {} {\textbf{w}}_{2}={\textbf{w}}_{2}>0,~~{\textbf{w}}_{3}=\frac{\mu \alpha _{1}{\textbf{w}}_{2}}{\vartheta _{1}+\phi _{1}},~~{\textbf{w}}_{4}=\frac{(1-\mu )\alpha _{1}{\textbf{w}}_{2}}{\vartheta _{1}+\phi _{2}+\delta },~~{\textbf{w}}_{5}=\frac{\phi _{1}{\textbf{w}}_{3}+\phi _{2}{\textbf{w}}_{4}}{\vartheta _{1}},\nonumber \\ {}{} & {} {\textbf{w}}_{6}=\frac{\tau \gamma _{{\textbf{h}}}^{*}(\vartheta _{2}\Phi _{{\textbf{v}}}{\textbf{w}}_{8}-\phi _{1}\Lambda _{2})}{\vartheta _{2}\Phi _{{\textbf{v}}}(\upsilon +\vartheta _{2})},~~{\textbf{w}}_{7}=\frac{\tau \gamma _{{\textbf{h}}}^{*}(\vartheta _{2}\Phi _{{\textbf{v}}}{\textbf{w}}_{8}+\phi _{1}\Lambda _{2}{\textbf{w}}_{9})}{\vartheta _{2}\Phi _{{\textbf{v}}}(\upsilon +\vartheta _{2}+\alpha _{2})},~~{\textbf{w}}_{8}=\frac{\alpha _{2}{\textbf{w}}_{7}}{\upsilon +\vartheta _{2}},\nonumber \\ {}{} & {} {\textbf{w}}_{9}=\frac{\beta _{1}{\textbf{w}}_{3}+\beta _{2}{\textbf{w}}_{4}+\beta _{3}{\textbf{w}}_{8}}{\xi _{2}+\xi _{3}},~~{\textbf{w}}_{10}=\frac{\xi _{3}{\textbf{w}}_{9}}{\xi _{2}}. \end{aligned}$$

Additionally, a left eigenvector (connected to zero eigenvalue) provided by $\bar{u}=(\Im _{1},\Im _{2},\Im _{3},\Im _{4},\Im _{5},\Im _{6},\Im _{7},\Im _{8},\Im _{9},\Im _{10})^{{\mathbb {T}}},$ which meets $\bar{u}.\bar{w}=1$ is procured by transposing the matrix presented as

$$\begin{aligned} {\mathcal {J}}_{{\mathcal {E}}_{0}}=\begin{pmatrix} -\vartheta _{1}&{}0&{}0&{}0&{}0&{}0&{}0&{}0&{}0&{}0\\ 0&{}-(\vartheta _{1}+\alpha _{1})&{}\mu \alpha _{1}&{}(1-\mu )\alpha _{1}&{}0&{}0&{}0&{}0&{}0&{}0\\ -\gamma _{{\textbf{h}}}^{*}\rho _{2}&{}\gamma _{{\textbf{h}}}^{*}\rho _{2}&{}-(\vartheta _{1}+\phi _{1})&{}0&{}\phi _{1}&{}0&{}0&{}0&{}\beta _{1}&{}0\\ -\gamma _{{\textbf{h}}}^{*}\rho _{1}&{}\gamma _{{\textbf{h}}}^{*}\rho _{1}&{}0&{}-(\vartheta _{1}+\phi _{2}+\delta )&{}\phi _{2}&{}0&{}0&{}0&{}\beta _{2}&{}0\\ 0&{}0&{}0&{}0&{}-\vartheta _{1}&{}0&{}0&{}0&{}0&{}0\\ 0&{}0&{}0&{}0&{}0&{}-(\upsilon +\vartheta _{2})&{}0&{}0&{}0&{}0\\ 0&{}0&{}0&{}0&{}0&{}0&{}-(\vartheta _{2}+\upsilon +\alpha _{2})&{}\alpha _{2}&{}0&{}0\\ -\frac{\gamma _{{\textbf{h}}}^{*}\vartheta _{2}\Lambda _{1}}{\vartheta _{1}\Lambda _{2}}&{}\frac{\gamma _{{\textbf{h}}}^{*}\vartheta _{2}\Lambda _{1}}{\vartheta _{1}\Lambda _{2}}&{}0&{}0&{}0&{}-\tau \gamma _{{\textbf{h}}}^{*}&{}\tau \gamma _{{\textbf{h}}}^{*}&{}-(\upsilon +\vartheta _{2})&{}\beta _{3}&{}0\\ -\frac{\gamma _{{\textbf{h}}}^{*}\rho _{3}\Lambda _{1}}{\vartheta _{1}\Phi _{{\textbf{v}}}}&{}\frac{\gamma _{{\textbf{h}}}^{*}\rho _{3}\Lambda _{1}}{\vartheta _{1}\Phi _{{\textbf{v}}}}&{}0&{}0&{}0&{}-\frac{\tau \gamma _{{\textbf{h}}}^{*}\phi _{1}\Lambda _{2}}{\vartheta _{2}\Phi _{{\textbf{v}}}}&{}\frac{\tau \gamma _{{\textbf{h}}}^{*}\phi _{1}\Lambda _{2}}{\vartheta _{2}\Phi _{{\textbf{v}}}}&{}0&{}-(\xi _{2}+\xi _{3})&{}\xi _{3}\\ -\frac{\Lambda _{1}\rho _{4}\gamma _{{\textbf{h}}}^{*}}{\vartheta _{1}\Phi _{{\textbf{v}}}}&{}\frac{\Lambda _{1}\rho _{4}\gamma _{{\textbf{h}}}^{*}}{\vartheta _{1}\Phi _{{\textbf{v}}}}&{}0&{}0&{}0&{}0&{}0&{}0&{}0&{}-\xi _{2} \end{pmatrix} \end{aligned}$$

The above system of equations gives

17 $$\begin{aligned}{} & {} \Im _{1}=0,~~\Im _{2}=\Im _{2}>0,~~\Im _{3}=\frac{\gamma _{{\textbf{h}}}^{*}\rho _{2}\Im _{2}+\beta _{1}\Im _{9}}{\vartheta _{1}+\phi _{1}},~~\Im _{4}=\frac{\gamma _{{\textbf{h}}}^{*}\rho _{1}\Im _{2}+\beta _{2}\Im _{9}}{\vartheta _{1}+\delta +\phi _{2}},~~\Im _{5}=0,~~\Im _{6}=0,\nonumber \\ {}{} & {} \Im _{7}=\frac{\alpha _{2}\Im _{8}}{\vartheta _{2}+\upsilon +\alpha _{2}},~~\Im _{8}=\frac{\tau \gamma _{{\textbf{h}}}^{*}\Im _{7}}{\upsilon +\vartheta _{2}}+\frac{\gamma _{{\textbf{h}}}^{*}\vartheta _{2}\Lambda _{1} \Im _{2}}{\vartheta _{1}\Lambda _{2}(\upsilon +\vartheta _{2})}+\frac{\beta _{3}\Im _{9}}{\vartheta _{2}+\upsilon },\nonumber \\ {}{} & {} \Im _{9}=\frac{\gamma _{{\textbf{h}}}^{*}\rho _{3}\Lambda _{1} \Im _{2}}{\vartheta _{1}\Phi _{{\textbf{v}}}(\xi _{2}+\xi _{3})}+\frac{\tau \gamma _{{\textbf{h}}}^{*}\phi _{1}\Lambda _{2}\Im _{7}}{\vartheta _{2}\Phi _{{\textbf{v}}}(\xi _{2}+\xi _{3})}+\frac{\xi _{3}\Im _{10}}{\xi _{2}+\xi _{3}},~~\Im _{10}=\frac{\gamma _{{\textbf{h}}}^{*}\rho _{4}\Lambda _{1} \Im _{2}}{\vartheta _{1}\xi _{2}\Phi _{{\textbf{v}}}}. \end{aligned}$$

To obtain, we employ the property $u_{{\textbf{m}}}\dot{w}_{{\textbf{m}}}=1,~({\textbf{m}}=1,...,10).$ Then, taking ${\textbf{w}}_{2}=1,$ without loss of generality provides us $\Im _{2}=\frac{1}{1+({\mathcal {H}}_{1}{\textbf{w}}_{3}+{\mathcal {H}}_{2}{\textbf{w}}_{4}+{\mathcal {H}}_{3}{\textbf{w}}_{7}+{\mathcal {H}}_{4}{\textbf{w}}_{8}+{\mathcal {H}}_{5}{\textbf{w}}_{9}+{\mathcal {H}}_{6}{\textbf{w}}_{10})}>0,$ where

18 $$\begin{aligned}{} & {} {\mathcal {H}}_{1}=\frac{\gamma _{{\textbf{h}}}}{\vartheta _{1}+\phi _{1}}\bigg \{\rho _{2}+\frac{\Lambda _{1}\vartheta _{2}\beta _{1}(\tau \gamma _{{\textbf{h}}}\xi _{2}\phi _{1}\alpha _{2}+(\rho _{3}\xi _{2}+\rho _{4}\xi _{3})(\upsilon ^{2}+\vartheta _{2}^{2}+(\upsilon -\tau \gamma _{{\textbf{h}}})\alpha _{2}+\vartheta _{2}(2\upsilon +\alpha _{2}))}{\xi _{2}\vartheta _{1}\vartheta _{2}(-\tau \Lambda _{2}\gamma _{{\textbf{h}}}\phi _{1}\beta _{3}\alpha _{2}+\Phi _{{\textbf{v}}}\vartheta _{2}(\xi _{2}+\xi _{3})(\upsilon ^{2}+\vartheta _{2}^{2}+(\upsilon -\tau \gamma _{{\textbf{h}}})\alpha _{2}+\vartheta _{2}(\alpha _{2}+2\upsilon )))}\bigg \},\nonumber \\ {}{} & {} {\mathcal {H}}_{2}=\frac{\gamma _{{\textbf{h}}}}{\vartheta _{1}+\phi _{2}+\delta }\bigg \{\rho _{2}+\frac{\Lambda _{1}\vartheta _{2}\beta _{1}(\tau \gamma _{{\textbf{h}}}\xi _{2}\phi _{1}\alpha _{2}+(\rho _{3}\xi _{2}+\rho _{4}\xi _{3})(\upsilon ^{2}+\vartheta _{2}^{2}+(\upsilon -\tau \gamma _{{\textbf{h}}})\alpha _{2}+\vartheta _{2}(2\upsilon +\alpha _{2}))}{\xi _{2}\vartheta _{1}\vartheta _{2}(-\tau \Lambda _{2}\gamma _{{\textbf{h}}}\phi _{1}\beta _{3}\alpha _{2}+\Phi _{{\textbf{v}}}\vartheta _{2}(\xi _{2}+\xi _{3})(\upsilon ^{2}+\vartheta _{2}^{2}+(\upsilon -\tau \gamma _{{\textbf{h}}})\alpha _{2}+\vartheta _{2}(\alpha _{2}+2\upsilon )))}\bigg \},\nonumber \\ {}{} & {} {\mathcal {H}}_{3}=-\frac{\Lambda _{1}\gamma _{{\textbf{h}}}\vartheta _{2}\alpha _{2}\big (\Phi _{{\textbf{v}}}\xi _{2}\vartheta _{2}(\xi _{2}+\xi _{3})+\Lambda _{2}\beta _{3}(\rho _{3}\xi _{2}+\rho _{4}\xi _{3})\big )}{\Lambda _{2}\xi _{2}\vartheta _{1}(\tau \Lambda _{2}\gamma _{{\textbf{h}}}\phi _{1}\beta _{3}\alpha _{2}-\vartheta _{1}\Phi _{{\textbf{v}}}(\xi _{2}+\xi _{3}))(\upsilon ^{2}+\vartheta _{2}^{2}+(\upsilon -\tau \gamma _{{\textbf{h}}})\alpha 
_{2}+\vartheta _{2}(2\upsilon +\alpha _{2}))},~~{\mathcal {H}}_{4}=\frac{\gamma _{{\textbf{h}}}\rho _{4}\Lambda _{1}}{\vartheta _{1}\xi _{2}\Phi _{{\textbf{v}}}},\nonumber \\ {}{} & {} {\mathcal {H}}_{5}=\frac{\Lambda _{1}\gamma _{{\textbf{h}}}\vartheta _{2}\big (\tau \gamma _{{\textbf{h}}}\xi _{2}\phi _{1}\alpha _{2}+(\rho _{3}\xi _{2}+\rho _{4}\xi _{3})(\upsilon ^{2}+\vartheta _{2}^{2}+(\upsilon -\tau \gamma _{{\textbf{h}}})\alpha _{2}+\vartheta _{2}(2\upsilon +\alpha _{2}))\big )}{\vartheta _{1}\xi _{2}(-\tau \Lambda _{2}\gamma _{{\textbf{h}}}\phi _{1}\beta _{3}\alpha _{2}+\Phi _{{\textbf{v}}}\vartheta _{2}(\xi _{2}+\xi _{3})(\upsilon ^{2}+\vartheta _{2}^{2}+(\upsilon -\tau \gamma _{{\textbf{h}}})\alpha _{2}+\vartheta _{2}(2\upsilon +\alpha _{2})))}. \end{aligned}$$

The specified formula is satisfied by this valuation of $\Im _{2}$ and ${\textbf{w}}_{2}$. To obtain the second order partial derivatives of $g_{{\textbf{m}}}$ at the steady state ${\mathcal {E}}_{0},$ we determine

19 $$\begin{aligned}{} & {} \frac{\partial ^{2}g_{2}}{\partial {\textbf{y}}_{1}\partial {\textbf{y}}_{8}}=\frac{\gamma _{{\textbf{h}}}\vartheta _{2}}{\Lambda _{2}},~~\frac{\partial ^{2}g_{2}}{\partial {\textbf{y}}_{1}\partial {\textbf{y}}_{9}}=\frac{\gamma _{{\textbf{h}}}\rho _{3}}{\Phi _{{\textbf{v}}}},~~ \frac{\partial ^{2}g_{2}}{\partial {\textbf{y}}_{1}\partial {\textbf{y}}_{10}}=\frac{\gamma _{{\textbf{h}}}\rho _{4}}{\Phi _{{\textbf{v}}}},~~\frac{\partial ^{2}g_{2}}{\partial {\textbf{y}}_{2}\partial {\textbf{y}}_{3}}=\frac{-\gamma _{{\textbf{h}}}\rho _{2}\vartheta _{1}}{\Lambda _{1}},\nonumber \\ {}{} & {} \frac{\partial ^{2}g_{2}}{\partial {\textbf{y}}_{2}\partial {\textbf{y}}_{4}}=\frac{-\gamma _{{\textbf{h}}}\vartheta _{1}\rho _{1}}{\Lambda _{1}},~~\frac{\partial ^{2}g_{2}}{\partial {\textbf{y}}_{3}\partial {\textbf{y}}_{4}}=\frac{-\gamma _{{\textbf{h}}}\vartheta _{1}(\rho _{1}+\rho _{2})}{\Lambda _{1}},~~ \frac{\partial ^{2}g_{2}}{\partial {\textbf{y}}_{3}\partial {\textbf{y}}_{5}}=\frac{-\gamma _{{\textbf{h}}}\rho _{2}\vartheta _{1}}{\Lambda _{1}},~~\frac{\partial ^{2}g_{2}}{\partial {\textbf{y}}_{3}\partial {\textbf{y}}_{3}}=\frac{-2\gamma _{{\textbf{h}}}\rho _{2}\vartheta _{1}}{\Lambda _{1}},\nonumber \\ {}{} & {} \frac{\partial ^{2}g_{2}}{\partial {\textbf{y}}_{4}\partial {\textbf{y}}_{4}}=\frac{-2\gamma _{{\textbf{h}}}\rho _{1}\vartheta _{1}}{\Lambda _{1}},~~\frac{\partial ^{2}g_{2}}{\partial {\textbf{y}}_{4}\partial {\textbf{y}}_{5}}=\frac{-\gamma _{{\textbf{h}}}\rho _{1}\vartheta _{1}}{\Lambda _{1}},~~ \frac{\partial ^{2}g_{2}}{\partial {\textbf{y}}_{6}\partial {\textbf{y}}_{8}}=\frac{-\gamma _{{\textbf{h}}}\Lambda _{1}\vartheta _{2}^{2}}{\vartheta _{1}\Lambda _{2}^{2}},~~\frac{\partial ^{2}g_{2}}{\partial {\textbf{y}}_{7}\partial {\textbf{y}}_{8}}=\frac{-\gamma _{{\textbf{h}}}\Lambda _{1}\vartheta _{2}^{2}}{\Lambda _{2}^{2}\vartheta _{1}},\nonumber \\ {}{} & {} \frac{\partial ^{2}g_{2}}{\partial {\textbf{y}}_{8}\partial {\textbf{y}}_{8}}=\frac{-2\gamma _{{\textbf{h}}}\vartheta _{2}^{2}\Lambda _{1}}{\Lambda _{2}^{2}\vartheta _{1}},~~\frac{\partial ^{2}g_{7}}{\partial {\textbf{y}}_{7}\partial {\textbf{y}}_{8}}=\frac{-\gamma _{{\textbf{h}}}\vartheta _{2}}{\Lambda _{2}},~~ \frac{\partial ^{2}g_{7}}{\partial {\textbf{y}}_{8}\partial {\textbf{y}}_{8}}=\frac{-2\gamma _{{\textbf{h}}}\vartheta _{2}}{\Lambda _{2}},~~\frac{\partial ^{2}g_{7}}{\partial {\textbf{y}}_{6}\partial {\textbf{y}}_{9}}=\frac{\gamma _{{\textbf{h}}}\phi _{1}}{\Phi _{{\textbf{v}}}}.\end{aligned}$$

Now, we calculates $\bar{a}$ and $\bar{b}$ parameters to obtain

$$\begin{aligned} \bar{a}{} & {} =\sum \limits _{\kappa ,\iota ,{\textbf{k}}=1}^{10}u_{\kappa }w_{\iota }w_{{\textbf{k}}}\frac{\partial ^{2}g_{\kappa }(0,0)}{\partial {\textbf{y}}_{\iota }\partial {\textbf{y}}_{{\textbf{k}}}}\nonumber \\ {}{} & {} =\frac{\gamma _{{\textbf{h}}}\vartheta _{2}}{\Lambda _{2}}\Im _{2}{\textbf{w}}_{1}^{2}-\frac{\gamma _{{\textbf{h}}}\Lambda _{1}\vartheta _{2}^{2}}{\Lambda _{2}^{2}\Lambda _{1}}\Im _{2}{\textbf{w}}_{6}{\textbf{w}}_{8}-({\textbf{w}}_{7}+2{\textbf{w}}_{8}){\textbf{w}}_{8}\Im _{2}-\frac{\gamma _{{\textbf{h}}}\rho _{1}\vartheta _{1}}{\Lambda _{1}}\Im _{2}{\textbf{w}}_{4}({\textbf{w}}_{2}+w_{3+2{\textbf{w}}_{4}+{\textbf{w}}_{5}})\nonumber \\ {}{} & {} \quad -\frac{\gamma _{{\textbf{h}}}\vartheta _{2}}{\Lambda _{2}}\Im _{7}{\textbf{w}}_{8}({\textbf{w}}_{7}+2{\textbf{w}}_{8})-\frac{\gamma _{{\textbf{h}}}\vartheta _{1}\rho _{2}}{\Lambda _{1}}\Im _{2}{\textbf{w}}_{3}({\textbf{w}}_{2}+{\textbf{w}}_{5}+2{\textbf{w}}_{3}+{\textbf{w}}_{4})+\frac{\gamma _{{\textbf{h}}}\phi _{1}}{\Phi _{{\textbf{v}}}}\Im _{7}{\textbf{w}}_{6}{\textbf{w}}_{9}\nonumber \\ {}{} & {} \quad +\frac{\gamma _{{\textbf{h}}}(\rho _{3}{\textbf{w}}_{9}+\rho _{4}{\textbf{w}}_{10})\Im _{2}{\textbf{w}}_{1}}{\Phi _{{\textbf{v}}}} \end{aligned}$$

and

$$\begin{aligned} \bar{b}{} & {} =\sum \limits _{\kappa ,\iota ,{\textbf{k}}=1}^{10}u_{\kappa }w_{\iota }\frac{\partial ^{2}g_{\kappa }(0,0)}{\partial {\textbf{y}}_{\iota }\partial \gamma _{{\textbf{h}}}}\nonumber \\ {}{} & {} =\frac{\Lambda _{1}\vartheta _{2}}{\vartheta _{1}\Phi _{{\textbf{v}}}}\Im _{2}{\textbf{w}}_{8}+\frac{\Lambda _{1}\rho _{3}}{\vartheta _{1}\Phi _{{\textbf{v}}}}\Im _{2}{\textbf{w}}_{9}+\frac{\Lambda _{1}\rho _{4}}{\vartheta _{1}\Phi _{{\textbf{v}}}}\Im _{2}{\textbf{w}}_{10}+\frac{\Lambda _{2}\phi _{1}}{\vartheta _{2}\Phi _{{\textbf{v}}}}\Im _{7}{\textbf{w}}_{9}+\Im _{7}{\textbf{w}}_{8}+\Im _{2}{\textbf{w}}_{3}\rho _{2}+\Im _{2}{\textbf{w}}_{4}\rho _{1}, \end{aligned}$$

where

20 $$\begin{aligned} \gamma _{\bar{w}\bar{u}}{} & {} =\frac{\gamma _{{\textbf{h}}}\phi _{1}}{\Phi _{{\textbf{v}}}}\Im _{7}{\textbf{w}}_{6}{\textbf{w}}_{9}+\frac{\gamma _{{\textbf{h}}}(\rho _{3}{\textbf{w}}_{9}+\rho _{4}{\textbf{w}}_{10})\Im _{2}{\textbf{w}}_{1}}{\Phi _{{\textbf{v}}}}-\frac{\gamma _{{\textbf{h}}}\rho _{1}\vartheta _{1}}{\Lambda _{1}}\Im _{2}{\textbf{w}}_{4}({\textbf{w}}_{2}+w_{3+2{\textbf{w}}_{4}+{\textbf{w}}_{5}})\nonumber \\ {}{} & {} \quad -\frac{\gamma _{{\textbf{h}}}\vartheta _{2}}{\Lambda _{2}}\Im _{7}{\textbf{w}}_{8}({\textbf{w}}_{7}+2{\textbf{w}}_{8})-\frac{\gamma _{{\textbf{h}}}\vartheta _{1}\rho _{2}}{\Lambda _{1}}\Im _{2}{\textbf{w}}_{3}({\textbf{w}}_{2}+{\textbf{w}}_{5}+2{\textbf{w}}_{3}+{\textbf{w}}_{4})-\Im _{2}{\textbf{w}}_{8}({\textbf{w}}_{7}+2{\textbf{w}}_{8}). \end{aligned}$$

$$\square$$

Bifurcations are important in dynamics investigations. As a result, in this part, bifurcation analysis is used to investigate the rich dynamical behaviour of the Lassa fever model (3) as follows:

#### Theorem 2.3

The fractional-order Lassa fever model (7) possess:

(ai) If $\bar{a}<0$ if and only if $\Re _{0}>1$, then we get a unique endemic equilibria.

(aii)  If $\bar{b}<0$ and $\bar{a}=0$, then we get a unique endemic equilibria.

(aiii)  If $\bar{a}>0,~\bar{b}<0$ and $\bar{b}^{2}-4\bar{a}\gamma _{\bar{a\omega }}>0$, two endemic equilibria exists.

Theorem 3, Case (ai), clearly shows the existence of a unique endemic equilibrium of the fractional-order Lassa fever model (7). when $\Re _{0}>1$. From Case (aiii), we can see existence of bifurcation possibly, in which the local asymptotically stability of disease-free equilibrium coexists with local asymptotically stability of endemic equilibrium, when $\Re _{0}<1.$ To determine the occurrence of bifurcation in the Lassa fever model (7), we set $\bar{b}^{2}-4\bar{a}\gamma _{\bar{a\omega }}=0$, and then evaluating for their critical values of $\Re _{0}$, shown by $\Re _{c}$, given by $\Re _{c}=\sqrt{1-\frac{\bar{b}^{2}}{4\gamma _{{\textbf{h}}} \Lambda _{2}\xi _{2}\vartheta _{1}\phi _{2}\delta \gamma _{\bar{a\omega }}}}$. Bifurcation would occur for values of $\Re _{0}$ such that $\Re _{c}<\Re _{0}<1.$ Considering the parameter values shown in Table 2, except for $\zeta _{1}=0.167$. With these parameters and the rest from Table 2, we have $\Re _{0}=0.5321<1$, indicating that the fractional-order Lassa fever model (7) exhibits bifurcation.

The occurrence of bifurcation in the fractional-order Lassa fever transmission model (7) has important epidemiological implications. It implies that the conventional criterion of $\Re _{0}<1$ is no longer sufficient for disease eradication, although it is still necessary. In this case, disease eradication would be determined by the initial sizes of the sub population in the model (i.e., state variables). Therefore, the practicality of controlling Lassa fever when $\Re _{0}<1$ may depend on the starting sizes of the sub population.

**Table 2:** Explanation of system’s feature.

*Symbols*	Values	References
$\mu$	0.8	^63^
$\delta$	0.0005	^64^
$\Lambda _{1}$	0.497	Estimated
$\Lambda _{2}$	2.74	Estimated
$\vartheta _{1}$	0.0000497	^65^
$\vartheta _{2}$	0.00274	^66^
$\phi _{1}$	0.0000476	Calculated
$\phi _{2}$	0.0000323	Calculated
$\upsilon$	0.0006	^67^
$\xi _{2}$	0.01868	Calculated
$\xi _{3}$	0.00701	^68^
$\alpha _{1}$	0.0094	Estimated
$\alpha _{2}$	0.048	Estimated
$\zeta _{1}$	0.167	Assumed
$\beta _{1}$	0.0667	Estimated
$\beta _{2}$	0.0357	Estimated
$\beta _{3}$	0.02569	Estimated
$\rho _{1}$	0.94	Estimated
$\rho _{2}$	0.95	Estimated
$\rho _{3}$	0.9	Estimated
$\rho _{4}$	0.85	Estimated
$\gamma _{{\textbf{r}}}$	0.004	Estimated
$\gamma _{{\textbf{h}}}$	0.00017	Estimated

## Stochastic analysis

Before providing insights into the system dynamics of an Lassa fever model (5), we must guarantee that the solution is both global and non-negative. The existence and uniqueness of the global non-negative solution of system (5) with a certain non-negative initial value are guaranteed by the following formula.

### Theorem 3.1

Assume that there is an initial value ${\tilde{\Theta }}(0)=\Big ({\textbf{X}}_{{\textbf{h}}}(0),{\textbf{P}}_{{\textbf{h}}}(0), {\textbf{Q}}_{{\textbf{h}}{\textbf{a}}}(0),{\textbf{Q}}_{{\textbf{h}}{\textbf{s}}}(0),{\textbf{R}}_{{\textbf{h}}}(0), {\textbf{X}}_{{\textbf{r}}}(0),{\textbf{P}}_{{\textbf{r}}}(0), {\textbf{Q}}_{{\textbf{r}}}(0),{\textbf{G}}_{{\textbf{s}}}(0),{\textbf{G}}_{{\textbf{a}}}(0)\Big )\in \Re _{+}^{10},$ there is a non-negative solution ${\tilde{\Theta }}({\textbf{t}})=\Big ({\textbf{X}}_{{\textbf{h}}}({\textbf{t}}),{\textbf{P}}_{{\textbf{h}}}({\textbf{t}}), {\textbf{Q}}_{{\textbf{h}}{\textbf{a}}}({\textbf{t}}),{\textbf{Q}}_{{\textbf{h}}{\textbf{s}}}({\textbf{t}}),{\textbf{R}}_ {{\textbf{h}}}({\textbf{t}}),{\textbf{X}}_{{\textbf{r}}}({\textbf{t}}),{\textbf{P}}_{{\textbf{r}}}({\textbf{t}}), {\textbf{Q}}_{{\textbf{r}}}({\textbf{t}}),{\textbf{G}}_{{\textbf{s}}}({\textbf{t}}),{\textbf{G}}_{{\textbf{a}}}({\textbf{t}})\Big )$ of the stochastic system (5) for ${\textbf{t}}\ge 0$ and the solution will stay in $\Re _{+}^{10}$ almost surely (a.s).

### Proof

Because the parameters in the mathematical formulas are locally Lipschitz continuous for the specified preliminary community composition ${\tilde{\Theta }}(0)\in \Re _{+}^{10}$, there exists a distinctive local solution ${\tilde{\Theta }}({\textbf{t}})\in \Re _{+}^{10}$ when ${\textbf{t}}\in [0,\tau _{\epsilon })$ (for information, see^50^). To demonstrate that this finding is global in nature, we must demonstrate that $\tau _{\epsilon }=\infty$ a.s. Suppose ${\mathfrak {T}}\ge 0$ be large enough that ${\tilde{\Theta }}(0)$ all fall inside that interval $\big [\frac{1}{{\mathfrak {T}}_{0}},{\mathfrak {T}}_{0}\big ]$. Determine the stopping time for every integer ${\mathfrak {T}}\ge {\mathfrak {T}}_{0}$. Introducing the stopping time

21 $$\begin{aligned} \tau _{{\mathfrak {T}}}=\inf \Big \{{\textbf{t}}\in (0,\tau _{\epsilon }):\min \{{\tilde{\Theta }}({\textbf{t}})\}\le \frac{1}{{\mathfrak {T}}} ~~or~~\max \{{\tilde{\Theta }}({\textbf{t}})\}\ge {{\mathfrak {T}}}\Big \}. \end{aligned}$$

In this investigation, we designated $\inf \emptyset =\infty$ so when $\emptyset$ signifies the empty set. By interpretation, $\tau _{{\mathfrak {T}}}$ increases as ${\mathfrak {T}}\mapsto \infty$. Select $\tau _{\infty }=\lim \limits _{{\mathfrak {T}}\mapsto \infty }\tau _{{\mathfrak {T}}}$ having $\tau _{\infty }\in [0,\tau _{\epsilon }]$ a.s. By asserting $\tau _{\infty }=\infty$ a.s., we can show that $\tau _{\epsilon }=\infty$ and ${\tilde{\Theta }}({\textbf{t}})$ a.s for all ${\textbf{t}}\ge 0$. To put it another way, we have to demonstrate that $\tau _{\epsilon }=\infty$ a.s. If the assertion is false, a couple of parameters ${\tilde{T}}>0$ and $\varepsilon \in (0,1)$ exist such that

22 $$\begin{aligned} {\mathcal {P}}\{\tau _{\infty }\le {\tilde{T}}\}>\varepsilon .\end{aligned}$$

Since ${\textbf{N}}_{{\textbf{h}}}({\textbf{t}})={\textbf{X}}_{{\textbf{h}}}({\textbf{t}}),{\textbf{P}}_{{\textbf{h}}}({\textbf{t}}),{\textbf{Q}}_{{\textbf{h}}{\textbf{a}}}({\textbf{t}}),{\textbf{Q}}_{{\textbf{h}}{\textbf{s}}}({\textbf{t}}),{\textbf{R}}_{{\textbf{h}}}({\textbf{t}}),$ then for ${\textbf{t}}\le \tau _{{\mathfrak {T}}},$ as is evident,

23 $$\begin{aligned} d{\textbf{N}}({\textbf{t}})\le (\Lambda _{1}-\vartheta _{1}{\textbf{N}}_{{\textbf{h}}}({\textbf{t}}))d{\textbf{t}}.\end{aligned}$$

By attempting to solve (23), we obtain

24 $$\begin{aligned} {\textbf{N}}_{{\textbf{h}}}({\textbf{t}})\le {\left\{ \begin{array}{ll} \frac{\Lambda _{1}}{\vartheta _{1}}~~~~~~~~if~~{\textbf{N}}_{{\textbf{h}}}(0)\le \frac{\Lambda _{1}}{\vartheta _{1}}\\ {\textbf{N}}_{{\textbf{h}}}(0),~~~~if~~{\textbf{N}}_{{\textbf{h}}}(0)>\frac{\Lambda _{1}}{\vartheta _{1}}, \end{array}\right. }:=\tilde{{\textbf{U}}} \end{aligned}$$

Analogously, we assume ${\textbf{N}}_{{\textbf{r}}}({\textbf{t}})={\textbf{X}}_{{\textbf{r}}}({\textbf{t}}),{\textbf{P}}_{{\textbf{r}}}({\textbf{t}}),{\textbf{Q}}_{{\textbf{r}}}({\textbf{t}}),{\textbf{G}}_{{\textbf{s}}}({\textbf{t}}),{\textbf{G}}_{{\textbf{a}}}({\textbf{t}}) ,$ then for ${\textbf{t}}\le \tau _{{\mathfrak {T}}},$ we have

25 $$\begin{aligned} d{\textbf{N}}_{{\textbf{r}}}({\textbf{t}})\le \big (\Lambda _{2}-(\upsilon +\vartheta _{2}){\textbf{N}}_{R{1}}\big )d{\textbf{t}}. \end{aligned}$$

Again, solving (25), we have

26 $$\begin{aligned} {\textbf{N}}_{{\textbf{r}}}({\textbf{t}})\le {\left\{ \begin{array}{ll} \frac{\Lambda _{2}}{\vartheta _{2}}~~~~~~~~if~~{\textbf{N}}_{{\textbf{r}}}(0)\le \frac{\Lambda _{2}}{\vartheta _{2}}\\ {\textbf{N}}_{{\textbf{r}}}(0),~~~~if~~{\textbf{N}}_{{\textbf{r}}}(0)>\frac{\Lambda _{1}}{\mu _{0}}, \end{array}\right. }:=M_{2}. \end{aligned}$$

Furthermore, we introduce a $\bar{C}^{2}$ mapping ${\textbf{h}}:\Re _{+}^{10}\mapsto \Re _{+}$ such that

27 $$\begin{aligned} {\textbf{h}}({\tilde{\Theta }}({\textbf{t}})){} & {} ={\textbf{X}}_{{\textbf{h}}}({\textbf{t}}),{\textbf{P}}_{{\textbf{h}}}({\textbf{t}}),{\textbf{Q}}_{{\textbf{h}}{\textbf{a}}}({\textbf{t}}),{\textbf{Q}}_{{\textbf{h}}{\textbf{s}}}({\textbf{t}}),{\textbf{R}}_{{\textbf{h}}}({\textbf{t}})+{\textbf{X}}_{{\textbf{r}}}({\textbf{t}}),{\textbf{P}}_{{\textbf{r}}}({\textbf{t}}),{\textbf{Q}}_{{\textbf{r}}}({\textbf{t}}),{\textbf{G}}_{{\textbf{s}}}({\textbf{t}}),{\textbf{G}}_{{\textbf{a}}}({\textbf{t}})\nonumber \\ {}{} & {} \quad -10-\big (\ln {\textbf{X}}_{{\textbf{h}}}({\textbf{t}})+\ln {\textbf{P}}_{{\textbf{h}}}({\textbf{t}})+\ln {\textbf{Q}}_{{\textbf{h}}{\textbf{a}}}({\textbf{t}})+\ln {\textbf{Q}}_{{\textbf{h}}{\textbf{s}}}({\textbf{t}})+\ln {\textbf{R}}_{{\textbf{h}}}({\textbf{t}})+\ln {\textbf{X}}_{{\textbf{r}}}({\textbf{t}})\nonumber \\ {}{} & {} \quad +\ln {\textbf{P}}_{{\textbf{r}}}({\textbf{t}})+\ln {\textbf{Q}}_{{\textbf{r}}}({\textbf{t}})+\ln {\textbf{G}}_{{\textbf{s}}}({\textbf{t}})+\ln {\textbf{G}}_{{\textbf{a}}}({\textbf{t}})\big ). \end{aligned}$$

Obviously, the function ${\textbf{h}}$ is positive, as demonstrated by the reality that ${\textbf{y}}_{1}-ln(e{\textbf{y}}_{1})\ge 0$ for all ${\textbf{y}}_{1}\ge 0$. Assume that ${\mathfrak {T}}\ge {\mathfrak {T}}_{0}$ and ${\tilde{T}}>0$ be arbitrary, and the Itô methodology applied to (27) generates

28 $$\begin{aligned} d{\textbf{h}}({\tilde{\Theta }}){} & {} =\Big (1-\frac{1}{{\textbf{X}}_{{\textbf{h}}}}\Big )+\sigma _{1}({\textbf{X}}_{{\textbf{h}}}-1)d{\mathcal {B}}_{1}({\textbf{t}})+\Big (1-\frac{1}{{\textbf{P}}_{{\textbf{h}}}}\Big )+\sigma _{2}({\textbf{P}}_{{\textbf{h}}}-1)d{\mathcal {B}}_{2}({\textbf{t}})\nonumber \\ {}{} & {} \quad + \Big (1-\frac{1}{{\textbf{Q}}_{{\textbf{h}}{\textbf{a}}}}\Big )+\sigma _{3}({\textbf{Q}}_{{\textbf{h}}{\textbf{a}}}-1)d{\mathcal {B}}_{3}({\textbf{t}})+\Big (1-\frac{1}{{\textbf{Q}}_{{\textbf{h}}{\textbf{s}}}}\Big )+\sigma _{4}({\textbf{Q
}}_{{\textbf{h}}{\textbf{s}}}-1)d{\mathcal {B}}_{4}({\textbf{t}})\nonumber \\ {}{} & {} \quad + \Big (1-\frac{1}{{\textbf{R}}_{{\textbf{h}}}}\Big )+\sigma _{5}({\textbf{R}}_{{\textbf{h}}}-1)d{\mathcal {B}}_{5}({\textbf{t}})+\Big (1-\frac{1}{{\textbf{X}}_{{\textbf{r}}}}\Big )+\sigma _{6}({\textbf{X}}_{{\textbf{r}}}-1)d{\mathcal {B}}_{6}({\textbf{t}})\nonumber \\ {}{} & {} \quad + \Big (1-\frac{1}{{\textbf{P}}_{{\textbf{r}}}}\Big )+\sigma _{7}({\textbf{P}}_{{\textbf{r}}}-1)d{\mathcal {B}}_{7}({\textbf{t}})+\Big (1-\frac{1}{{\textbf{Q}}_{{\textbf{r}}}}\Big )+\sigma _{8}({\textbf{Q}}_{{\textbf{r}}}-1)d{\mathcal {B}}_{8}({\textbf{t}})\nonumber \\ {}{} & {} \quad + \Big (1-\frac{1}{{\textbf{G}}_{{\textbf{s}}}}\Big )+\sigma _{9}({\textbf{G}}_{{\textbf{s}}}-1)d{\mathcal {B}}_{9}({\textbf{t}})+\Big (1-\frac{1}{{\textbf{G}}_{{\textbf{a}}}}\Big )+\sigma _{10}({\textbf{G}}_{{\textbf{a}}}-1)d{\mathcal {B}}_{10}({\textbf{t}})\nonumber \\ {}{} & {} ={\mathcal {L}}{\textbf{h}}({\tilde{\Theta }})d{\textbf{t}}+\sigma _{1}({\textbf{X}}_{{\textbf{h}}}-1)d{\mathcal {B}}_{1}({\textbf{t}})+\sigma _{2}({\textbf{P}}_{{\textbf{h}}}-1)d{\mathcal {B}}_{2}({\textbf{t}})+\sigma _{3}({\textbf{Q}}_{{\textbf{h}}{\textbf{a}}}-1)d{\mathcal {B}}_{3}({\textbf{t}})\nonumber \\ {}{} & {} \quad +\sigma _{4}({\textbf{Q}}_{{\textbf{h}}{\textbf{s}}}-1)d{\mathcal {B}}_{4}({\textbf{t}})+\sigma _{5}({\textbf{R}}_{{\textbf{h}}}-1)d{\mathcal {B}}_{5}({\textbf{t}})+\sigma _{6}({\textbf{X}}_{{\textbf{r}}}-1)d{\mathcal {B}}_{6}({\textbf{t}})+\sigma _{7}({\textbf{P}}_{{\textbf{r}}}-1)d{\mathcal {B}}_{7}({\textbf{t}})\nonumber \\ {}{} & {} \quad +\sigma _{8}({\textbf{Q}}_{{\textbf{r}}}-1)d{\mathcal {B}}_{8}({\textbf{t}})+ \sigma _{9}({\textbf{G}}_{{\textbf{s}}}-1)d{\mathcal {B}}_{9}({\textbf{t}})+\sigma _{10}({\textbf{G}}_{{\textbf{a}}}-1)d{\mathcal {B}}_{10}({\textbf{t}}).\end{aligned}$$

In view of (28), ${\mathcal {L}}{\textbf{h}}:\Re _{+}^{10}\mapsto \Re _{+}$ the continuity formula defines as

29 $$\begin{aligned} {\mathcal {L}}{\textbf{h}}({\tilde{\Theta }}){} & {} =\Big (1-\frac{1}{{\textbf{X}}_{{\textbf{h}}}}\Big )\Big \{\Lambda _{1}-\Upsilon _{{\textbf{h}}}{\textbf{X}}_{{\textbf{h}}}-\vartheta _{1}{\textbf{X}}_{{\textbf{h}}}\Big \}+\frac{\sigma _{1}^{2}}{2}\nonumber \\ {}{} & {} \quad +\Big (1-\frac{1}{{\textbf{P}}_{{\textbf{h}}}}\Big )\Big \{\Upsilon _{{\textbf{h}}}{\textbf{X}}_{{\textbf{h}}}-(\alpha _{1}+\vartheta _{1}){\textbf{P}}_{{\textbf{h}}}\Big \}+\frac{\sigma _{2}^{2}}{2}\nonumber \\ {}{} & {} \quad + \Big (1-\frac{1}{{\textbf{Q}}_{{\textbf{h}}{\textbf{a}}}}\Big )\Big \{\mu \alpha _{1}{\textbf{P}}_{{\textbf{h}}}-(\phi _{1}+\vartheta _{1}){\textbf{Q}}_{{\textbf{h}}{\textbf{a}}}\Big \}+\frac{\sigma _{3}^{2}}{2}\nonumber \\ {}{} & {} \quad +\Big (1-\frac{1}{{\textbf{Q}}_{{\textbf{h}}{\textbf{s}}}}\Big )\Big \{(1-\mu )\alpha _{1}{\textbf{P}}_{{\textbf{h}}}-(\delta +\phi _{2}+\vartheta _{1}){\textbf{Q}}_{{\textbf{h}}{\textbf{s}}}\Big \}+\frac{\sigma _{4}^{2}}{2}\nonumber \\ {}{} & {} \quad + \Big (1-\frac{1}{{\textbf{R}}_{{\textbf{h}}}}\Big )\Big \{\phi _{1}{\textbf{P}}_{{\textbf{h}}}+\phi _{2}{\textbf{Q}}_{{\textbf{h}}{\textbf{s}}}-\vartheta _{1}{\textbf{R}}_{{\textbf{h}}}\Big \}+\frac{\sigma _{5}^{2}}{2}\nonumber \\ {}{} & {} \quad +\Big (1-\frac{1}{{\textbf{X}}_{{\textbf{r}}}}\Big )\Big \{\Lambda _{2}-\Upsilon _{{\textbf{r}}}{\textbf{X}}_{{\textbf{r}}}-(\vartheta _{2}+\upsilon ){\textbf{R}}_{{\textbf{h}}}\Big \}+\frac{\sigma _{6}^{2}}{2}\nonumber \\ {}{} & {} \quad + \Big (1-\frac{1}{{\textbf{P}}_{{\textbf{r}}}}\Big )\Big \{\Upsilon _{{\textbf{r}}}{\textbf{X}}_{{\textbf{r}}}-(\alpha _{2}+\vartheta _{2}+\upsilon ){\textbf{P}}_{{\textbf{h}}}\Big \}+\frac{\sigma _{7}^{2}}{2}\nonumber \\ {}{} & {} \quad +\Big (1-\frac{1}{{\textbf{Q}}_{{\textbf{r}}}}\Big )\Big \{\alpha _{2}{\textbf{P}}_{{\textbf{r}}}-(\upsilon +\vartheta _{2}){\textbf{Q}}_{{\textbf{r}}}\Big \}+\frac{\sigma _{8}^{2}}{2}\nonumber \\ {}{} & {} \quad + \Big (1-\frac{1}{{\textbf{G}}_{{\textbf{s}}}}\Big )\Big \{\beta _{1}{\textbf{Q}}_{{\textbf{h}}{\textbf{a}}}+\beta _{2}{\textbf{Q}}_{{\textbf{h}}{\textbf{s}}}+\beta _{3}{\textbf{Q}}_{{\textbf{r}}}-(\xi _{2}+\xi _{3}){\textbf{G}}_{{\textbf{s}}}\Big \}+\frac{\sigma _{9}^{2}}{2}\nonumber \\ {}{} & {} \quad +\Big (1-\frac{1}{{\textbf{G}}_{{\textbf{a}}}}\Big )\Big \{\xi _{3}{\textbf{G}}_{{\textbf{s}}}-\xi _{2}{\textbf{G}}_{{\textbf{a}}}\Big \}+\frac{\sigma _{10}^{2}}{2}\nonumber \\ {}{} & {} \le \Lambda _{1}+\Lambda _{2}+\Upsilon _{{\textbf{h}}+5\vartheta _{1}}+\alpha _{1}+\phi _{1}+\delta +\phi _{2}+\Upsilon _{{\textbf{r}}}+\alpha _{2}+2\upsilon +3\vartheta _{2}+2\xi _{2}+\xi _{3}\nonumber \\ {}{} & {} \quad +\frac{\sigma _{1}^{2}+\sigma _{2}^{2}+\sigma _{3}^{2}+\sigma _{4}^{2}+\sigma _{5}^{2}+\sigma _{6}^{2}+\sigma _{7}^{2}+\sigma _{8}^{2}+\sigma _{9}^{2}+\sigma _{10}^{2}}{2}:={\mathcal {K}}. \end{aligned}$$

Here, ${\mathcal {K}}$ is a positive fixed number that is free of ${\tilde{\Theta }}({\textbf{t}})$ and ${\textbf{t}}$. Accordingly,

30 $$\begin{aligned} d{\textbf{h}}({\tilde{\Theta }}){} & {} \le {\mathcal {K}}d{\textbf{t}}+\sigma _{1}({\textbf{X}}_{{\textbf{h}}}-1)d{\mathcal {B}}_{1}({\textbf{t}})+\sigma _{2}({\textbf{P}}_{{\textbf{h}}}-1)d{\mathcal {B}}_{2}({\textbf{t}})+\sigma _{3}({\textbf{Q}}_{{\textbf{h}}{\textbf{a}}}-1)d{\mathcal {B}}_{3}({\textbf{t}})\nonumber \\ {}{} & {} \quad +\sigma _{4}({\textbf{Q}}_{{\textbf{h}}{\textbf{s}}}-1)d{\mathcal {B}}_{4}({\textbf{t}})+\sigma _{5}({\textbf{R}}_{{\textbf{h}}}-1)d{\mathcal {B}}_{5}({\textbf{t}})+\sigma _{6}({\textbf{X}}_{{\textbf{r}}}-1)d{\mathcal {B}}_{6}({\textbf{t}})+\sigma _{7}({\textbf{P}}_{{\textbf{r}}}-1)d{\mathcal {B}}_{7}({\textbf{t}})\nonumber \\ {}{} & {} \quad +\sigma _{8}({\textbf{Q}}_{{\textbf{r}}}-1)d{\mathcal {B}}_{8}({\textbf{t}})+ \sigma _{9}({\textbf{G}}_{{\textbf{s}}}-1)d{\mathcal {B}}_{9}({\textbf{t}})+\sigma _{10}({\textbf{G}}_{{\textbf{a}}}-1)d{\mathcal {B}}_{10}({\textbf{t}}).\end{aligned}$$

Performing integration over 0 to $\tau _{{\mathfrak {T}}}\wedge {\tilde{T}},$ we have

31 $$\begin{aligned} {\mathfrak {E}}\big [{\textbf{h}}\big ({\tilde{\Theta }}(\tau _{{\mathfrak {T}}}\wedge {\tilde{T}})\big )\big ]{} & {} \le {\textbf{h}}\big ({\tilde{\Theta }}(0)\big )+{\mathcal {K}}(\tau _{{\mathfrak {T}}}\wedge {\tilde{T}})\nonumber \\ {}{} & {} \quad +{\mathfrak {E}}\bigg \{\int \limits _{0}^{\tau _{{\mathfrak {T}}}\wedge {\tilde{T}}}\sigma _{1}({\textbf{X}}_{{\textbf{h}}}-1)d{\mathcal {B}}_{1}({\textbf{t}})+\sigma _{2}({\textbf{P}}_{{\textbf{h}}}-1)d{\mathcal {B}}_{2}({\textbf{t}})+\sigma _{3}({\textbf{Q}}_{{\textbf{h}}{\textbf{a}}}-1)d{\mathcal {B}}_{3}({\textbf{t}})\nonumber \\ {}{} & {} \quad +\sigma _{4}({\textbf{Q}}_{{\textbf{h}}{\textbf{s}}}-1)d{\mathcal {B}}_{4}({\textbf{t}})+\sigma _{5}({\textbf{R}}_{{\textbf{h}}}-1)d{\mathcal {B}}_{5}({\textbf{t}})+\sigma _{6}({\textbf{X}}_{{\textbf{r}}}-1)d{\mathcal {B}}_{6}({\textbf{t}})\nonumber \\ {}{} & {} \quad +\sigma _{7}({\textbf{P}}_{{\textbf{r}}}-1)d{\mathcal {B}}_{7}({\textbf{t}})+\sigma _{8}({\textbf{Q}}_{{\textbf{r}}}-1)d{\mathcal {B}}_{8}({\textbf{t}})+ \sigma _{9}({\textbf{G}}_{{\textbf{s}}}-1)d{\mathcal {B}}_{9}({\textbf{t}})\nonumber \\ {}{} & {} \quad +\sigma _{10}({\textbf{G}}_{{\textbf{a}}}-1)d{\mathcal {B}}_{10}({\textbf{t}})\bigg \}\nonumber \\ {}{} & {} \le {\textbf{h}}({\tilde{\Theta }}(0))+{\mathcal {K}}{\tilde{T}}. \end{aligned}$$

Inserting $\Pi _{{\mathfrak {T}}}=\tau _{{\mathfrak {T}}}\le {\tilde{T}}$ for ${\mathfrak {T}}\ge {\mathfrak {T}}_{1}$ and utilizing (32), ${\mathcal {P}}(\Pi _{{\mathfrak {T}}})\ge \varepsilon .$ Additionally, it is important to keep in mind that for every $\omega \in \Pi _{{\mathfrak {T}}},$ $\exists$ at least one ${\tilde{\Theta }}(\tau _{{\mathfrak {T}}},\omega )$ that are identical to ${\mathfrak {T}}$ or $1/{\mathfrak {T}},$ and therefore

$$\begin{aligned} {\textbf{h}}({\tilde{\Theta }}(\tau _{{\mathfrak {T}}})) \end{aligned}$$

is not less than ${\mathfrak {T}}-1-log{\mathfrak {T}}$ or $\frac{1}{{\mathfrak {T}}}-1+\log {\mathfrak {T}}.$ As a result,

$$\begin{aligned} {\textbf{h}}({\tilde{\Theta }}(\tau _{{\mathfrak {T}}}))\ge {\mathfrak {E}}\big ({\mathfrak {T}}-1-log{\mathfrak {T}}\big )\wedge \Big (\frac{1}{{\mathfrak {T}}}-1+\log {\mathfrak {T}}\Big ).\end{aligned}$$

As a result of (22) and (31), it describes that

$$\begin{aligned} {\textbf{h}}({\tilde{\Theta }}(0))+{\mathcal {K}}{\tilde{T}}{} & {} \ge {\mathfrak {E}}\big [1_{\Pi (\omega )}{\textbf{h}}({\tilde{\Theta }}(\tau _{{\mathfrak {T}}}))\big ]\nonumber \\ {}{} & {} \varepsilon \Big \{\big ({\mathfrak {T}}-1-log{\mathfrak {T}}\big )\wedge \Big (\frac{1}{{\mathfrak {T}}}-1+\log {\mathfrak {T}}\Big )\Big \}, \end{aligned}$$

where $1_{\Pi (\omega )}$ denotes the indicator mapping of $\Pi .$ Choosing ${\mathfrak {T}}\mapsto \infty$ shows the contradiction $\infty ={\tilde{T}}(\tilde{{\textbf{U}}}+M_{2})+{\textbf{h}}({\tilde{\Theta }}(0))<\infty ,$ which implies that $\tau _{\infty }=\infty$ a.s and this is the immediate consequence.$\square$

### Extinction and ergodic stationary distribution of Lassa fever model

We are interested in establishing adequate prerequisites for the extinction and existence-uniqueness of an ergodic stationary distribution of non-negative solutions to the dynamical model (5) in this segment. We begin by discussing a few explanations about stationary distribution (see; Khasminskii^59^). Allow for the sake of simplicity

32 $$\begin{aligned} \langle Z_{1}({\textbf{t}})\rangle =\frac{1}{{\textbf{t}}}\int \limits _{0}^{{\textbf{t}}}z_{1}(r_{1})dr_{1}. \end{aligned}$$

Our next result is the strong law of large numbers, which is mainly due to^60^.

#### Lemma 3.2

(^60^) Suppose there be a continuous and real-valued martingale, $\tilde{{\textbf{U}}}=\{\tilde{{\textbf{U}}}\}_{{\textbf{t}}\ge 0},$ which will be disappeared at ${\textbf{t}}\mapsto 0,$ then

33 $$\begin{aligned}{} & {} \lim \limits _{{\textbf{t}}\mapsto \infty }\langle \tilde{{\textbf{U}}},\tilde{{\textbf{U}}}\rangle _{{\textbf{t}}}=\infty ,~a.s,~\implies ~~\lim \limits _{{\textbf{t}}\mapsto \infty }\frac{\tilde{{\textbf{U}}}}{\langle \tilde{{\textbf{U}}},\tilde{{\textbf{U}}}\rangle _{{\textbf{t}}}}=0,~~a.s,~~and ~furthermore,\nonumber \\ {}{} & {} \lim \limits _{{\textbf{t}}\mapsto \infty }\sup \frac{\langle \tilde{{\textbf{U}}},\tilde{{\textbf{U}}}\rangle _{{\textbf{t}}}}{{\textbf{t}}}<0,~~a.s.,~\implies \lim \limits _{{\textbf{t}}\mapsto \infty }\frac{{\tilde{{\textbf{U}}}}_{{\textbf{t}}}}{{\textbf{t}}}=0,~a.s. \end{aligned}$$

#### Lemma 3.3

(^61,62^) Suppose there is a function $\hbar \in \bar{C}([0,\infty )\times \Pi (0,\infty ))$ and ${\mathcal {H}}_{1}\in \bar{C}([0,\infty )\times \Pi ,\Re ).$ Suppose there are positive constants $\Upsilon _{0},\Upsilon _{1}$ and ${\tilde{T}}$ such that

34 $$\begin{aligned}{} & {} \log \hbar ({\textbf{t}})\le -\Upsilon _{0}\int \limits _{0}^{{\textbf{t}}}\hbar (s_{1})ds_{1}+\Upsilon _{1} {\textbf{t}}+{\mathcal {H}}_{1}({\textbf{t}})~a.s.,\forall ~{\textbf{t}}\ge {\tilde{T}}~~~and ~~~\lim \limits _{{\textbf{t}}\mapsto \infty }\frac{{\mathcal {H}}_{1}({\textbf{t}})}{{\textbf{t}}}=0,~~a.s,\nonumber \\ {}{} & {} then~~\lim \limits _{{\textbf{t}}\mapsto \infty }\frac{1}{{\textbf{t}}}\int \limits _{0}^{{\textbf{t}}}\hbar (s_{1})ds_{1}\le \frac{\Upsilon _{1}}{\Upsilon _{0}},~~a.s. \end{aligned}$$

Let us identify some other threshold parameter for our immediate plans.

35 $$\begin{aligned} \Re _{0}^{p}=\frac{\mu _{1}(1-\mu _{1})\alpha _{1}^{2}\alpha _{2}}{(\phi _{1}+\vartheta _{1}+\frac{\sigma _{3}^{2}}{2})(\delta +\phi _{2}+\vartheta _{2}+\frac{\sigma _{4}^{2}}{2})(\upsilon +\vartheta _{2}+\frac{\sigma _{8}^{2}}{2})}. \end{aligned}$$

We will discover the prerequisites that will cause the ailment to become extirpated in the population. The aforementioned assumption is made for this reason and must be proven.

#### Theorem 3.4

If $\Re _{0}^{p}<1,$ then the disease ${\textbf{Q}}_{{\textbf{h}}{\textbf{a}}},{\textbf{Q}}_{{\textbf{h}}{\textbf{s}}}$ and ${\textbf{Q}}_{{\textbf{r}}}$ will wipe out exponentially with unit probability, that is.,

36 $$\begin{aligned}{} & {} \lim \limits _{{\textbf{t}}\mapsto \infty }\sup \frac{\log {\textbf{Q}}_{{\textbf{h}}{\textbf{a}}}}{{\textbf{t}}}\le \big (\phi _{1}+\vartheta _{1}+\frac{\sigma _{3}^{2}}{2}\big )\big [\Re _{0}^{p}-1\big ]<0~~a.s,\nonumber \\ {}{} & {} \lim \limits _{{\textbf{t}}\mapsto \infty }\sup \frac{\log {\textbf{Q}}_{{\textbf{h}}{\textbf{s}}}}{{\textbf{t}}}\le \big (\delta +\phi _{2}+\vartheta _{1}+\frac{\sigma _{4}^{2}}{2}\big )\big [\Re _{0}^{p}-1\big ]<0~~a.s, \end{aligned}$$

and

37 $$\begin{aligned} \lim \limits _{{\textbf{t}}\mapsto \infty }\sup \frac{\log {\textbf{Q}}_{{\textbf{r}}}}{{\textbf{t}}}\le \big (\upsilon +\vartheta _{2}+\frac{\sigma _{8}^{2}}{2}\big )\big [\Re _{0}^{p}-1\big ]<0~~a.s. \end{aligned}$$

Also,

38 $$\begin{aligned}{} & {} \lim \limits _{{\textbf{t}}\mapsto \infty }{\textbf{X}}_{{\textbf{h}}}({\textbf{t}})=\frac{\Lambda _{1}}{\vartheta _{1}},~~~\lim \limits _{{\textbf{t}}\mapsto \infty }{\textbf{P}}_{{\textbf{h}}}({\textbf{t}})=0,~~~\lim \limits _{{\textbf{t}}\mapsto \infty }{\textbf{Q}}_{{\textbf{h}}{\textbf{a}}}({\textbf{t}})=0,~~~~\lim \limits _{{\textbf{t}}\mapsto \infty }{\textbf{Q}}_{{\textbf{h}}{\textbf{s}}}({\textbf{t}})=0,\nonumber \\ {}{} & {} \lim \limits _{{\textbf{t}}\mapsto \infty }{\textbf{R}}_{{\textbf{h}}}({\textbf{t}})=0,\lim \limits _{{\textbf{t}}\mapsto \infty }{\textbf{X}}_{{\textbf{r}}}({\textbf{t}})=\frac{\Lambda _{2}}{\upsilon +\vartheta _{2}},~~~\lim \limits _{{\textbf{t}}\mapsto \infty }{\textbf{P}}_{{\textbf{r}}}({\textbf{t}})=0,~~\lim \limits _{{\textbf{t}}\mapsto \infty }{\textbf{Q}}_{{\textbf{r}}}({\textbf{t}})=0,\nonumber \\ {}{} & {} \lim \limits _{{\textbf{t}}\mapsto \infty }{\textbf{G}}_{{\textbf{s}}}({\textbf{t}})=0,~~~\lim \limits _{{\textbf{t}}\mapsto \infty }{\textbf{G}}_{{\textbf{a}}}({\textbf{t}})=0 \end{aligned}$$

#### Proof

Performing the integration on both sides of the proposed model (5) yields the following formulas

39 $$\begin{aligned}{} & {} \frac{{\textbf{X}}_{{\textbf{h}}}({\textbf{t}})-{\textbf{X}}_{{\textbf{h}}}(0)}{{\textbf{t}}}+\frac{{\textbf{P}}_{{\textbf{h}}}({\textbf{t}})-{\textbf{P}}_{{\textbf{h}}}(0)}{{\textbf{t}}}+\frac{{\textbf{Q}}_{{\textbf{h}}{\textbf{a}}}({\textbf{t}})-{\textbf{Q}}_{{\textbf{h}}{\textbf{a}}}(0)}{{\textbf{t}}}+\frac{{\textbf{Q}}_{{\textbf{h}}{\textbf{s}}}({\textbf{t}})-{\textbf{Q}}_{{\textbf{h}}{\textbf{s}}}(0)}{{\textbf{t}}}\nonumber \\ {}{} & {} \quad +\frac{{\textbf{R}}_{{\textbf{h}}}({\textbf{t}})-{\textbf{R}}_{{\textbf{h}}}(0)}{{\textbf{t}}}+\frac{{\textbf{G}}_{{\textbf{s}}}({\textbf{t}})-{\textbf{G}}_{{\textbf{a}}}(0)}{{\textbf{t}}}+\frac{{\textbf{G}}_{{\textbf{a}}}({\textbf{t}})-{\textbf{G}}_{{\textbf{a}}}(0)}{{\textbf{t}}}\nonumber \\ {}{} & {} =\Lambda _{1}-\vartheta _{1}\langle {\textbf{X}}_{{\textbf{h}}}({\textbf{t}})\rangle -\vartheta _{1}\langle {\textbf{P}}_{{\textbf{h}}}({\textbf{t}})\rangle -\vartheta _{1}\langle {\textbf{Q}}_{{\textbf{h}}{\textbf{a}}}({\textbf{t}})\rangle -(\delta +\vartheta _{1})\langle {\textbf{Q}}_{{\textbf{h}}{\textbf{s}}}({\textbf{t}})\rangle -\vartheta _{1}\langle {\textbf{R}}_{{\textbf{h}}}({\textbf{t}})\rangle \nonumber \\ {}{} & {} \quad +\beta _{1}\langle {\textbf{Q}}_{{\textbf{h}}{\textbf{a}}}\rangle +\beta _{2}\langle {\textbf{Q}}_{{\textbf{h}}{\textbf{s}}}\rangle +\beta _{3}\langle {\textbf{Q}}_{{\textbf{r}}}\rangle -\xi _{2}\langle {\textbf{G}}_{{\textbf{s}}}\rangle -\xi _{2}\langle {\textbf{G}}_{{\textbf{a}}}\rangle +\frac{\sigma _{1}}{{\textbf{t}}}\int \limits _{0}^{{\textbf{t}}}{\textbf{X}}_{{\textbf{h}}}d{\mathcal {B}}_{1}({\textbf{t}})+\frac{\sigma _{2}}{{\textbf{t}}}\int \limits _{0}^{{\textbf{t}}}{\textbf{P}}_{{\textbf{h}}}d{\mathcal {B}}_{2}({\textbf{t}})\nonumber \\ {}{} & {} \quad +\frac{\sigma _{3}}{{\textbf{t}}}\int \limits _{0}^{{\textbf{t}}}{\textbf{Q}}_{{\textbf{h}}{\textbf{a}}}d{\mathcal {B}}_{3}({\textbf{t}})+\frac{\sigma _{4}}{{\textbf{t}}}\int \limits _{0}^{{\textbf{t}}}{\textbf{Q}}_{{\textbf{h}}{\textbf{s}}}d{\mathcal {B}}_{4}({\textbf{t}})+\frac{\sigma _{5}}{{\textbf{t}}}\int \limits _{0}^{{\textbf{t}}}{\textbf{X}}_{{\textbf{h}}}d{\mathcal {B}}_{1}({\textbf{t}})+\frac{\sigma _{9}}{{\textbf{t}}}\int \limits _{0}^{{\textbf{t}}}{\textbf{G}}_{{\textbf{s}}}d{\mathcal {B}}_{9}({\textbf{t}})+\frac{\sigma _{10}}{{\textbf{t}}}\int \limits _{0}^{{\textbf{t}}}{\textbf{G}}_{{\textbf{a}}}d{\mathcal {B}}_{10}({\textbf{t}}).\nonumber \\ \end{aligned}$$

We utilize the conception $\phi ({\textbf{t}})$ in (39) for simplicity, and with several algebraic estimation, we emerge at the accompanying

$$\begin{aligned} \langle {\textbf{X}}_{{\textbf{h}}}({\textbf{t}})\rangle ={} & {} \frac{\Lambda _{1}}{\vartheta _{1}}-\frac{\delta +\vartheta _{1}}{\vartheta _{1}} \langle {\textbf{Q}}_{{\textbf{h}}{\textbf{s}}}({\textbf{t}})\rangle -\langle {\textbf{P}}_{{\textbf{h}}}({\textbf{t}})\rangle -\langle {\textbf{Q}}_{{\textbf{h}}{\textbf{a}}}({\textbf{t}})\rangle -\langle {\textbf{R}}_{{\textbf{h}}}({\textbf{t}})\rangle \nonumber \\ {}{} & {} \quad -\frac{1}{\vartheta _{1}}\big \{\beta _{1}\langle {\textbf{Q}}_{{\textbf{h}}{\textbf{a}}}\rangle +\beta _{2}\langle {\textbf{Q}}_{{\textbf{h}}{\textbf{s}}}\rangle +\beta _{3}\langle {\textbf{Q}}_{{\textbf{r}}}\rangle -\xi _{2}\langle {\textbf{G}}_{{\textbf{s}}}\rangle -\xi _{2}\langle {\textbf{G}}_{{\textbf{a}}}\rangle \big \}+\phi ({\textbf{t}}), \end{aligned}$$

where the value of $\phi ({\textbf{t}})$ is described as

$$\begin{aligned} \phi ({\textbf{t}}){} & {} =-\frac{1}{\vartheta _{1}}\bigg \{\frac{{\textbf{X}}_{{\textbf{h}}}({\textbf{t}})-{\textbf{X}}_{{\textbf{h}}}(0)}{{\textbf{t}}}+\frac{{\textbf{P}}_{{\textbf{h}}}({\textbf{t}})-{\textbf{P}}_{{\textbf{h}}}(0)}{{\textbf{t}}}+\frac{{\textbf{Q}}_{{\textbf{h}}{\textbf{a}}}({\textbf{t}})-{\textbf{Q}}_{{\textbf{h}}{\textbf{a}}}(0)}{{\textbf{t}}}+\frac{{\textbf{Q}}_{{\textbf{h}}{\textbf{s}}}({\textbf{t}})-{\textbf{Q}}_{{\textbf{h}}{\textbf{s}}}(0)}{{\textbf{t}}}\nonumber \\ {}{} & {} \quad +\frac{{\textbf{R}}_{{\textbf{h}}}({\textbf{t}})-{\textbf{R}}_{{\textbf{h}}}(0)}{{\textbf{t}}}+\frac{{\textbf{G}}_{{\textbf{s}}}({\textbf{t}})-{\textbf{G}}_{{\textbf{s}}}(0)}{{\textbf{t}}}+\frac{{\textbf{G}}_{{\textbf{a}}}({\textbf{t}})-{\textbf{G}}_{{\textbf{a}}}(0)}{{\textbf{t}}}\nonumber \\ {}{} & {} \quad -\bigg (\frac{\sigma _{2}}{{\textbf{t}}}\int \limits _{0}^{{\textbf{t}}}{\textbf{P}}_{{\textbf{h}}}d{\mathcal {B}}_{2}({\textbf{t}})+\frac{\sigma _{3}}{{\textbf{t}}}\int \limits _{0}^{{\textbf{t}}}{\textbf{Q}}_{{\textbf{h}}{\textbf{a}}}d{\mathcal {B}}_{3}({\textbf{t}})+\frac{\sigma _{4}}{{\textbf{t}}}\int \limits _{0}^{{\textbf{t}}}{\textbf{Q}}_{{\textbf{h}}{\textbf{s}}}d{\mathcal {B}}_{4}({\textbf{t}})+\frac{\sigma _{5}}{{\textbf{t}}}\int \limits _{0}^{{\textbf{t}}}{\textbf{X}}_{{\textbf{h}}}d{\mathcal {B}}_{1}({\textbf{t}})\nonumber \\ {}{} & {} \quad +\frac{\sigma _{9}}{{\textbf{t}}}\int \limits _{0}^{{\textbf{t}}}{\textbf{G}}_{{\textbf{s}}}d{\mathcal {B}}_{9}({\textbf{t}})+\frac{\sigma _{10}}{{\textbf{t}}}\int \limits _{0}^{{\textbf{t}}}{\textbf{G}}_{{\textbf{a}}}d{\mathcal {B}}_{10}({\textbf{t}})\bigg )\bigg \}. \end{aligned}$$

Clearly, we have

$$\begin{aligned} \lim \limits _{{\textbf{t}}\mapsto \infty }\phi ({\textbf{t}})=0~~a.s. \end{aligned}$$

Similarly, we integrate both sides of the last three cohorts of the developed framework (5), yielding the formula given

40 $$\begin{aligned}{} & {} \frac{{\textbf{X}}_{{\textbf{r}}}({\textbf{t}})-{\textbf{X}}_{{\textbf{r}}}(0)}{{\textbf{t}}}+\frac{{\textbf{P}}_{{\textbf{r}}}({\textbf{t}})-{\textbf{P}}_{{\textbf{r}}}(0)}{{\textbf{t}}}+\frac{{\textbf{Q}}_{{\textbf{r}}}({\textbf{t}})-{\textbf{Q}}_{{\textbf{r}}}(0)}{{\textbf{t}}}\nonumber \\ {}{} & {} =\Lambda _{2}-(\upsilon +\vartheta _{2})\big \{\langle {\textbf{X}}_{{\textbf{r}}}\rangle +\langle {\textbf{P}}_{{\textbf{r}}}\rangle +\langle {\textbf{Q}}_{{\textbf{r}}}\rangle \big \}+\frac{\sigma _{6}}{{\textbf{t}}}\int \limits _{0}^{{\textbf{t}}}{\textbf{X}}_{{\textbf{r}}}d{\mathcal {B}}_{6}({\textbf{t}})+\frac{\sigma _{7}}{{\textbf{t}}}\int \limits _{0}^{{\textbf{t}}}{\textbf{P}}_{{\textbf{r}}}d{\mathcal {B}}_{7}({\textbf{t}})+\frac{\sigma _{8}}{{\textbf{t}}}\int \limits _{0}^{{\textbf{t}}}{\textbf{Q}}_{{\textbf{r}}}d{\mathcal {B}}_{8}({\textbf{t}}).\nonumber \\ \end{aligned}$$

We are able to determine the following expression by employing certain representations in (40) and performing several algebraic calculations

$$\begin{aligned} \langle {\textbf{X}}_{{\textbf{r}}}\rangle =\frac{\Lambda _{2}}{\upsilon +\vartheta _{2}}-\langle {\textbf{P}}_{{\textbf{r}}}\rangle -\langle {\textbf{Q}}_{{\textbf{r}}}\rangle +\Psi ({\textbf{t}}). \end{aligned}$$

The significance of $\Psi ({\textbf{t}})$ is characterized as

$$\begin{aligned} \Psi ({\textbf{t}}){} & {} =-\frac{1}{\upsilon +\vartheta _{2}}\bigg (\frac{{\textbf{X}}_{{\textbf{r}}}({\textbf{t}})-{\textbf{X}}_{{\textbf{r}}}(0)}{{\textbf{t}}}+\frac{{\textbf{P}}_{{\textbf{r}}}({\textbf{t}})-{\textbf{P}}_{{\textbf{r}}}(0)}{{\textbf{t}}}+\frac{{\textbf{Q}}_{{\textbf{r}}}({\textbf{t}})-{\textbf{Q}}_{{\textbf{r}}}(0)}{{\textbf{t}}}-\frac{\sigma _{6}}{{\textbf{t}}}\int \limits _{0}^{{\textbf{t}}}{\textbf{X}}_{{\textbf{r}}}d{\mathcal {B}}_{6}({\textbf{t}})\nonumber \\ {}{} & {} \quad -\frac{\sigma _{7}}{{\textbf{t}}}\int \limits _{0}^{{\textbf{t}}}{\textbf{P}}_{{\textbf{r}}}d{\mathcal {B}}_{7}({\textbf{t}})-\frac{\sigma _{8}}{{\textbf{t}}}\int \limits _{0}^{{\textbf{t}}}{\textbf{Q}}_{{\textbf{r}}}d{\mathcal {B}}_{8}({\textbf{t}})\bigg ). \end{aligned}$$

Evidently, $$\lim \limits _{{\textbf{t}}\mapsto \infty }\Psi ({\textbf{t}})=0,~a.s.$$

Implementing the Itô strategy to framework (5) third cohort, integrating over $[0,{\textbf{t}}]$, and then dividing by ${\textbf{t}}$ produces

41 $$\begin{aligned} d\log {\textbf{Q}}_{{\textbf{h}}{\textbf{a}}}({\textbf{t}})=\bigg (\mu \alpha _{1}\frac{{\textbf{P}}_{{\textbf{h}}}}{{\textbf{Q}}_{{\textbf{h}}{\textbf{a}}}}-(\phi _{1}+\vartheta _{1})-\frac{\sigma _{3}^{2}}{2}\bigg )d{\textbf{t}}+\frac{\sigma _{3}}{{\textbf{t}}}\int \limits _{0}^{{\textbf{t}}}d{\mathcal {B}}_{3}({\textbf{t}}) \end{aligned}$$

By integrating (41) over $[0,{\textbf{t}}]$ and dividing it by ${\textbf{t}}$ leads to

42 $$\begin{aligned} \log {\textbf{Q}}_{{\textbf{h}}{\textbf{a}}}({\textbf{t}})-\log {\textbf{Q}}_{{\textbf{h}}{\textbf{a}}}(0){} & {} \le \int \limits _{0}^{{\textbf{t}}}\bigg (\mu \alpha _{1}-(\phi _{1}+\vartheta _{1})-\frac{\sigma _{3}^{2}}{2}\bigg )ds_{1}+{\sigma _{3}}{\mathcal {B}}_{3}({\textbf{t}})\nonumber \\ {}{} & {} \le \bigg (\mu \alpha _{1}-(\phi _{1}+\vartheta _{1})-\frac{\sigma _{3}^{2}}{2}\bigg ){\textbf{t}}+{\sigma _{3}}{\mathcal {B}}_{3}({\textbf{t}})\nonumber \\ {}{} & {} =\big (\phi _{1}+\vartheta _{1}+\frac{\sigma _{3}^{2}}{2}\big )\bigg [\frac{\mu _{1}\alpha _{1}}{\phi _{1}+\vartheta _{1}+\frac{\sigma _{3}^{2}}{2}}-1\bigg ]t_{2}+\sigma _{3}{\mathcal {B}}_{3}({\textbf{t}})\nonumber \\ {}{} & {} \le \big (\phi _{1}+\vartheta _{1}+\frac{\sigma _{3}^{2}}{2}\big )\big [\Re _{0}^{p}-1\big ]{\textbf{t}}+\sigma _{3}{\mathcal {B}}_{3}({\textbf{t}}). \end{aligned}$$

Employing the Lemma 3.2 for local martingales, we acquire

$$\begin{aligned} \lim \limits _{{\textbf{t}}\mapsto \infty }\frac{{\mathcal {B}}_{3}({\textbf{t}})}{{\textbf{t}}}=0~~a.s. \end{aligned}$$

In view of limit superior of both sides

$$\begin{aligned} \lim \limits _{{\textbf{t}}\mapsto \infty }\sup \frac{\log {\textbf{Q}}_{{\textbf{h}}{\textbf{a}}}}{{\textbf{t}}}\le \big (\phi _{1}+\vartheta _{1}+\frac{\sigma _{3}^{2}}{2}\big )\big [\Re _{0}^{p}-1\big ]<0~a.s. \end{aligned}$$

Therefore, whenever $\Re _{0}^{s}<1$ occurs, $\lim \limits _{{\textbf{t}}\mapsto \infty }{\textbf{Q}}_{{\textbf{h}}{\textbf{a}}}({\textbf{t}})=0,~a.s$ and $\lim \limits _{{\textbf{t}}\mapsto \infty }\langle {\textbf{Q}}_{{\textbf{h}}{\textbf{a}}}({\textbf{t}})\rangle =0,~a.s.$

Similarly, by employing the Itô technique to the fourth cohort of model (5), employing the limits $[0,{\textbf{t}}]$, and then dividing by ${\textbf{t}}$, we have

43 $$\begin{aligned} d\log {\textbf{Q}}_{{\textbf{h}}{\textbf{s}}}({\textbf{t}})=\bigg ((1-\mu )\alpha _{1}\frac{{\textbf{P}}_{{\textbf{h}}}}{{\textbf{Q}}_{{\textbf{h}}{\textbf{a}}}}-(\delta +\phi _{2}+\vartheta _{1})-\frac{\sigma _{4}^{2}}{2}\bigg )d{\textbf{t}}+\frac{\sigma _{4}}{{\textbf{t}}}\int \limits _{0}^{{\textbf{t}}}d{\mathcal {B}}_{4}({\textbf{t}}) \end{aligned}$$

By integrating (43) over $[0,{\textbf{t}}]$ and dividing it by ${\textbf{t}}$ leads to

44 $$\begin{aligned} \log {\textbf{Q}}_{{\textbf{h}}{\textbf{a}}}({\textbf{t}})-\log {\textbf{Q}}_{{\textbf{h}}{\textbf{a}}}(0){} & {} \le \int \limits _{0}^{{\textbf{t}}}\bigg ((1-\mu )\alpha _{1}-(\delta +\phi _{2}+\vartheta _{1})-\frac{\sigma _{4}^{2}}{2}\bigg )ds_{1}+{\sigma _{4}}{\mathcal {B}}_{4}({\textbf{t}})\nonumber \\ {}{} & {} \le \bigg ((1-\mu )\alpha _{1}-(\delta +\phi _{2}+\vartheta _{1})-\frac{\sigma _{4}^{2}}{2}\bigg ){\textbf{t}}+{\sigma _{4}}{\mathcal {B}}_{4}({\textbf{t}})\nonumber \\ {}{} & {} =\big (\delta +\phi _{2}+\vartheta _{1}+\frac{\sigma _{4}^{2}}{2}\big )\bigg [\frac{(1-\mu _{1})\alpha _{1}}{\delta +\phi _{2}+\vartheta _{1}+\frac{\sigma _{4}^{2}}{2}}-1\bigg ]t_{2}+\sigma _{4}{\mathcal {B}}_{4}({\textbf{t}})\nonumber \\ {}{} & {} \le \big (\delta +\phi _{2}+\vartheta _{1}+\frac{\sigma _{4}^{2}}{2}\big )\big [\Re _{0}^{p}-1\big ]{\textbf{t}}+\sigma _{4}{\mathcal {B}}_{4}({\textbf{t}}). \end{aligned}$$

Again, employing the Lemma 3.2 for local martingales, we acquire

$$\begin{aligned} \lim \limits _{{\textbf{t}}\mapsto \infty }\frac{{\mathcal {B}}_{4}({\textbf{t}})}{{\textbf{t}}}=0~~a.s. \end{aligned}$$

In view of limit superior of both sides

$$\begin{aligned} \lim \limits _{{\textbf{t}}\mapsto \infty }\sup \frac{\log {\textbf{Q}}_{{\textbf{h}}{\textbf{s}}}}{{\textbf{t}}}\le \big (\delta +\phi _{2}+\vartheta _{1}+\frac{\sigma _{4}^{2}}{2}\big )\big [\Re _{0}^{p}-1\big ]<0~a.s. \end{aligned}$$

Therefore, whenever $\Re _{0}^{s}<1$ occurs, $\lim \limits _{{\textbf{t}}\mapsto \infty }{\textbf{Q}}_{{\textbf{h}}{\textbf{s}}}({\textbf{t}})=0,~a.s$ and $\lim \limits _{{\textbf{t}}\mapsto \infty }\langle {\textbf{Q}}_{{\textbf{h}}{\textbf{s}}}({\textbf{t}})\rangle =0,~a.s.$

in a similar manner, we can prove that

$$\begin{aligned} \lim \limits _{{\textbf{t}}\mapsto \infty }\sup \frac{\log {\textbf{Q}}_{{\textbf{r}}}}{{\textbf{t}}}\le \big (\upsilon +\vartheta _{2}+\frac{\sigma _{8}^{2}}{2}\big )\big [\Re _{0}^{p}-1\big ]<0~a.s. \end{aligned}$$

Therefore, whenever $\Re _{0}^{s}<1$ occurs, $\lim \limits _{{\textbf{t}}\mapsto \infty }{\textbf{Q}}_{{\textbf{r}}}({\textbf{t}})=0,~a.s$ and $\lim \limits _{{\textbf{t}}\mapsto \infty }\langle {\textbf{Q}}_{{\textbf{r}}}({\textbf{t}})\rangle =0,~a.s.$

As a result, we noticed that illness extermination is determined by the setting of the parameter $\Re _{0}^{p}$, i.e., for $\Re _{0}^{p}<1$, the illness will eventually disappear. $\square$

Regardless of the omission of an EEP in the random perturbation model (5), we aim to explore the existence of an ergodic stationary distribution, which could prove disease perseverance more clearly. First, we shall discuss some of the results of Has’minskii’s notion. Additional data is available at^59^.

Suppose there is a homogeneous Markov process in $d_{1}$ (the $d_{1}$-dimensional Euclidean space) ${\textbf{Y}}({\textbf{t}})$ that efficiently deals with the stochastic differential equation below

45 $$\begin{aligned} d{\textbf{Y}}({\textbf{t}})=h_{1}({\textbf{y}})d{\textbf{t}}+\sum \limits _{\iota =1}^{n_{1}}g_{\iota }({\textbf{Y}})d{\mathcal {B}}_{\iota }({\textbf{t}}). \end{aligned}$$

The diffusion matrix ${\textbf{a}}({\textbf{y}})=(a_{\iota {\textbf{k}}}({\textbf{y}}))$ and $a_{\iota {\textbf{k}}}({\textbf{y}})=\sum \limits _{\kappa =1}^{n_{1}}g_{\kappa }^{(\iota )}({\textbf{y}})g_{\kappa }^{({\textbf{k}})}({\textbf{y}}).$

#### Lemma 3.5

(^50^) Assume a bounded domain ${\mathcal {U}}\subset \chi _{d_{1}}$ with regular boundary $\Gamma$ such that

($Z_{1}$) Suppose a positive number ${\textbf{M}}$ such that $\sum \limits _{\iota ,{\textbf{k}}=1}^{d_{1}}a_{\iota {\textbf{k}}}({\textbf{y}})\phi _{\iota }\phi _{{\textbf{k}}}\ge {\textbf{M}}\vert \phi \vert ^{2},~{\textbf{y}}\in {\mathcal {U}},~\phi \in \Re ^{d_{1}}.$

($Z_{2}$) $\exists$ a non-negative $\bar{C}^{2}$-mapping ${\mathcal {H}}$ such that ${\mathcal {L}}{\mathcal {H}}$ is negative $\forall$ ${\textbf{y}}\in \chi _{d_{1}}\setminus {\mathcal {U}}$(particularly ${\mathcal {L}}{\mathcal {H}}\le -1$, $\forall$ ${\textbf{y}}\in \chi _{d_{1}}\setminus {\mathcal {U}}$), then the Markov technique ${\textbf{Y}}({\textbf{t}})$ has a unique ergodic stationary distribution $\pi (.),$ and

46 $$\begin{aligned} {\mathbb {P}}\Big \{\lim \limits _{{\tilde{T}}\mapsto \infty }\frac{1}{{\tilde{T}}}\int \limits _{0}^{{\tilde{T}}}\hbar ({\textbf{Y}}({\textbf{t}}))d{\textbf{t}}=\int \limits _{\Upsilon _{d_{1}}}\hbar ({\textbf{y}})\pi (d{\textbf{y}})=1\Big \}, \end{aligned}$$

satisfies $\forall ~{\textbf{y}}\in \chi _{d_{1}},$ where $\hbar (.)$ is an integrable function in relation to the measure $\pi .$

In addition, based on Has’minskii’s theory^59^, we will demonstrate essentials that guarantees the presence of an ergodic stationary distribution.

#### Theorem 3.6

If

47 $$\begin{aligned} \Re _{0}^{s}=\frac{\Lambda _{1}\gamma _{{\textbf{h}}}\mu \alpha _{1}\Upsilon _{{\textbf{h}}}}{(\alpha _{1}+\vartheta _{1}+\frac{\sigma _{2}^{2}}{2})(\vartheta _{1}+\phi _{1}+\frac{\sigma _{3}^{2}}{2})(\vartheta _{1}+\frac{\sigma _{1}^{2}}{2})}+\frac{\Lambda _{1}\gamma _{{\textbf{h}}}(1-\mu )\alpha _{1}\Upsilon _{{\textbf{h}}}}{(\alpha _{1}+\vartheta _{1}+\frac{\sigma _{2}^{2}}{2})(\vartheta _{1}+\phi _{2}+\frac{\sigma _{4}^{2}}{2})(\vartheta _{1}+\frac{\sigma _{1}^{2}}{2})}>1, \end{aligned}$$

then the system (5) has a unique stationary distribution $\pi (.)$ and has the ergodic condition.

#### Proof

The argument is separated into two phases: the initial one is to demonstrate that the uniform elliptic scenario is fulfilled, and the subsequent step is to generate a positive Lyapunov function that meets the criteria $(Z_{2})$ of Lemma 3.5.

**Phase I**: The diffusion matrix of model (5) is presented as

$$\begin{aligned} {\mathcal {A}}=\begin{pmatrix} \varpi _{1}^{2}{\textbf{X}}_{{\textbf{h}}}^{2}&{}0&{}0&{}0&{}0&{}0&{}0&{}0&{}0&{}0\\ 0&{}\varpi _{2}^{2}{\textbf{P}}_{{\textbf{h}}}^{2}&{}0&{}0&{}0&{}0&{}0&{}0&{}0&{}0\\ 0&{}0&{}\varpi _{3}^{2}{\textbf{Q}}_{{\textbf{h}}{\textbf{a}}}^{2}&{}0&{}0&{}0&{}0&{}0&{}0&{}0\\ 0&{}0&{}0&{}\varpi _{4}^{2}{\textbf{Q}}_{{\textbf{h}}{\textbf{s}}}^{2}&{}0&{}0&{}0&{}0&{}0&{}0\\ 0&{}0&{}0&{}0&{}\varpi _{5}^{2}{\textbf{R}}_{{\textbf{h}}}^{2}&{}0&{}0&{}0&{}0&{}0\\ 0&{}0&{}0&{}0&{}0&{}\varpi _{6}^{2}{\textbf{X}}_{{\textbf{r}}}^{2}&{}0&{}0&{}0&{}0\\ 0&{}0&{}0&{}0&{}0&{}0&{}\varpi _{7}^{2}{\textbf{P}}_{{\textbf{r}}}^{2}&{}0&{}0&{}0\\ 0&{}0&{}0&{}0&{}0&{}0&{}0&{}\varpi _{8}^{2}{\textbf{Q}}_{{\textbf{r}}}^{2}&{}0&{}0\\ 0&{}0&{}0&{}0&{}0&{}0&{}0&{}0&{}\varpi _{9}^{2}{\textbf{G}}_{{\textbf{s}}}^{2}&{}0\\ 0&{}0&{}0&{}0&{}0&{}0&{}0&{}0&{}0&{}\varpi _{10}^{2}{\textbf{G}}_{{\textbf{a}}}^{2}\\ \end{pmatrix}. \end{aligned}$$

Selecting ${\mathbb {M}}=\min \limits _{{\tilde{\Theta }}\in {\mathbb {U}}_{{\mathfrak {T}}}\subset \Re _{10}^{+}}\big \{\varpi _{1}^{2}{\textbf{X}}_{{\textbf{h}}}^{2},\varpi _{2}^{2}{\textbf{P}}_{{\textbf{h}}}^{2},\varpi _{3}^{2}{\textbf{Q}}_{{\textbf{h}}{\textbf{a}}}^{2},\varpi _{4}^{2}{\textbf{Q}}_{{\textbf{h}}{\textbf{s}}}^{2},\varpi _{5}^{2}{\textbf{R}}_{{\textbf{h}}}^{2},\varpi _{6}^{2}{\textbf{X}}_{{\textbf{r}}}^{2},\varpi _{7}^{2}{\textbf{P}}_{{\textbf{r}}}^{2},\varpi _{8}^{2}{\textbf{Q}}_{{\textbf{r}}}^{2},\varpi _{9}^{2}{\textbf{G}}_{{\textbf{s}}}^{2},\varpi _{10}^{2}{\textbf{G}}_{{\textbf{a}}}^{2}\big \},$ we have

$$\begin{aligned} \sum \limits _{\iota ,{\textbf{k}}=1}^{10}{\bar{a}}_{\iota ,{\textbf{k}}}({\tilde{\Theta }})\varrho _{\iota ,{\textbf{k}}}{} & {} =\varpi _{1}^{2}{\textbf{X}}_{{\textbf{h}}}^{2}\varrho _{1}^{2}+\varpi _{2}^{2}{\textbf{P}}_{{\textbf{h}}}^{2}\varrho _{2}^{2}+\varpi _{3}^{2}{\textbf{Q}}_{{\textbf{h}}{\textbf{a}}}^{2}\varrho _{3}^{2}+\varpi _{4}^{2}{\textbf{Q}}_{{\textbf{h}}{\textbf{s}}}^{2}\varrho _{4}^{2}+\varpi _{5}^{2}{\textbf{R}}_{{\textbf{h}}}^{2}\varrho _{5}^{2}+\varpi _{6}^{2}{\textbf{X}}_{{\textbf{r}}}^{2}\varrho _{6}^{2}+\varpi _{7}^{2}{\textbf{P}}_{{\textbf{r}}}^{2}\varrho _{7}^{2}\nonumber \\ {}{} & \quad{} +\varpi _{8}^{2}{\textbf{Q}}_{{\textbf{r}}}^{2}\varrho _{8}^{2}+\varpi _{9}^{2}{\textbf{G}}_{{\textbf{s}}}^{2}\varrho _{9}^{2}+\varpi _{10}^{2}{\textbf{G}}_{{\textbf{a}}}^{2}\varrho _{10}^{2}\ge {\mathbb {M}}\Vert \varrho \Vert ^{2}, \end{aligned}$$

$\forall ~{\tilde{\Theta }}^{{\tilde{T}}}\in {\mathbb {U}}_{{\mathfrak {T}}}$ and $\varrho =(\varrho _{\iota })^{{\tilde{T}}}\in \Re _{+}^{10},~\iota =1,...,10,$ where ${\mathbb {U}}_{{\mathfrak {T}}}=\big [1/{\mathfrak {T}},{\mathfrak {T}}\big ]\times \big [1/{\mathfrak {T}},{\mathfrak {T}}\big ]\times \big [1/{\mathfrak {T}},{\mathfrak {T}}\big ]\times \big [1/{\mathfrak {T}},{\mathfrak {T}}\big ]\times \big [1/{\mathfrak {T}},{\mathfrak {T}}\big ]\times \big [1/{\mathfrak {T}},{\mathfrak {T}}\big ]\times \big [1/{\mathfrak {T}},{\mathfrak {T}}\big ]\times \big [1/{\mathfrak {T}},{\mathfrak {T}}\big ]\times \big [1/{\mathfrak {T}},{\mathfrak {T}}\big ]\times \big [1/{\mathfrak {T}},{\mathfrak {T}}\big ].$ Finally, the criteria $(Z_{1})$ in Lemma 3.5 satisfies.

**Phase II**: Assume that

48 $$\begin{aligned}{} & {} \bar{{\textbf{X}}_{{\textbf{h}}}}=\frac{\Lambda _{1}}{\Upsilon _{{\textbf{h}}}+\frac{\sigma _{1}^{2}}{2}},~~\bar{{\textbf{P}}_{{\textbf{h}}}}=1,~~\bar{{\textbf{Q}}_{{\textbf{h}}{\textbf{a}}}}=\frac{\mu \alpha _{1}{\textbf{P}}_{{\textbf{h}}}}{(\vartheta _{1}+\phi _{1}+\frac{\sigma _{3}^{2}}{2})},~~\bar{{\textbf{Q}}_{{\textbf{h}}{\textbf{s}}}}=\frac{(1-\mu )\alpha _{1}{\textbf{P}}_{{\textbf{h}}}}{(\delta +\vartheta _{1}+\phi _{2}+\frac{\sigma _{4}^{2}}{2})},~~\bar{{\textbf{R}}_{{\textbf{h}}}}=\frac{1}{\vartheta _{1}+\frac{\sigma _{5}^{2}}{2}},\nonumber \\ {}{} & {} \bar{{\textbf{X}}_{{\textbf{r}}}}=\frac{\Lambda _{2}}{\Upsilon _{{\textbf{r}}}+\upsilon +\vartheta _{2}+\frac{\sigma _{6}^{2}}{2}},~~\bar{{\textbf{P}}_{{\textbf{r}}}}=\frac{1}{\alpha _{2}+\upsilon +\vartheta _{2}+\frac{\sigma _{7}^{2}}{2}},~~\bar{{\textbf{Q}}_{{\textbf{r}}}}=\frac{\alpha _{2}{\textbf{P}}_{{\textbf{r}}}}{\upsilon +\vartheta _{2}+\frac{\sigma _{8}^{2}}{2}},~~\bar{{\textbf{G}}_{{\textbf{s}}}}=\frac{1}{\xi _{2}+\xi _{3}+\frac{\sigma _{9}^{2}}{2}},\nonumber \\ {}{} & {} \bar{{\textbf{G}}_{{\textbf{a}}}}=\frac{\xi _{3}}{\xi _{2}}+\frac{\sigma _{10}^{2}}{2},~~\tilde{{\textbf{X}}_{{\textbf{h}}}}=\frac{{\textbf{X}}_{{\textbf{h}}}}{\bar{{\textbf{X}}_{{\textbf{h}}}}},~~\tilde{{\textbf{P}}_{{\textbf{h}}}}=\frac{{\textbf{P}}_{{\textbf{h}}}}{\bar{{\textbf{P}}_{{\textbf{h}}}}},~~\tilde{{\textbf{Q}}_{{\textbf{h}}{\textbf{a}}}}=\frac{{\textbf{Q}}_{{\textbf{h}}{\textbf{a}}}}{\bar{{\textbf{Q}}_{{\textbf{h}}{\textbf{a}}}}},~~\tilde{{\textbf{Q}}_{{\textbf{h}}{\textbf{s}}}}=\frac{{\textbf{Q}}_{{\textbf{h}}{\textbf{s}}}}{\bar{{\textbf{Q}}_{{\textbf{h}}{\textbf{s}}}}},~~\tilde{{\textbf{R}}_{{\textbf{h}}}}=\frac{{\textbf{R}}_{{\textbf{h}}}}{\bar{{\textbf{R}}_{{\textbf{h}}}}},\nonumber \\ {}{} & {} \tilde{{\textbf{X}}_{{\textbf{r}}}}=\frac{{\textbf{X}}_{{\textbf{r}}}}{\bar{{\textbf{X}}_{{\textbf{r}}}}},~~\tilde{{\textbf{P}}_{{\textbf{r}}}}=\frac{{\textbf{P}}_{{\textbf{r}}}}{\bar{{\textbf{P}}_{{\textbf{r}}}}},~~\tilde{{\textbf{Q}}_{{\textbf{r}}}}=\frac{{\textbf{Q}}_{{\textbf{r}}}}{\bar{{\textbf{Q}}_{{\textbf{r}}}}},~~\tilde{{\textbf{G}}_{{\textbf{s}}}}=\frac{{\textbf{G}}_{{\textbf{s}}}}{\bar{{\textbf{G}}_{{\textbf{s}}}}},~~\tilde{{\textbf{G}}_{{\textbf{a}}}}=\frac{{\textbf{G}}_{{\textbf{a}}}}{\bar{{\textbf{G}}_{{\textbf{a}}}}}. \end{aligned}$$

Using the fact of model (5), we have

49 $$\begin{aligned} {\mathbb {L}}(-\ln {\textbf{X}}_{{\textbf{h}}}){} & {} =-\frac{\Lambda _{1}}{{\textbf{X}}_{{\textbf{h}}}}+\Upsilon _{{\textbf{h}}}+\vartheta _{1}+\frac{\sigma _{1}^{2}}{2}\nonumber \\ {}{} & {} =-\frac{\Lambda _{1}}{\tilde{{\textbf{X}}_{{\textbf{h}}}}\bar{{\textbf{X}}_{{\textbf{h}}}}}+\Upsilon _{{\textbf{h}}}+\vartheta _{1}+\frac{\sigma _{1}^{2}}{2}\nonumber \\ {}{} & {} \le -\frac{\Lambda _{1}}{\bar{{\textbf{X}}_{{\textbf{h}}}}}\Big (\ln \frac{1}{\tilde{{\textbf{X}}_{{\textbf{h}}}}}+1\Big )+\Upsilon _{{\textbf{h}}}+\vartheta _{1}+\frac{\sigma _{1}^{2}}{2}\nonumber \\ {}{} & {} =\frac{\Lambda _{1}}{\bar{{\textbf{X}}_{{\textbf{h}}}}}\ln \tilde{{\textbf{X}}_{{\textbf{h}}}}+\gamma _{{\textbf{h}}}\Big (\frac{{\textbf{Q}}_{{\textbf{r}}}}{{\textbf{N}}_{{\textbf{r}}}}+\frac{\rho _{1}{\textbf{Q}}_{{\textbf{h}}{\textbf{s}}}}{{\textbf{N}}_{{\textbf{h}}}}+\frac{\rho _{2}{\textbf{Q}}_{{\textbf{h}}{\textbf{a}}}}{{\textbf{N}}_{{\textbf{h}}}}+1+\frac{\rho _{3}{\textbf{G}}_{{\textbf{s}}}}{\Phi _{{\textbf{v}}}}+\frac{\rho _{4}{\textbf{G}}_{{\textbf{a}}}}{\Phi _{{\textbf{v}}}}\Big ), \end{aligned}$$

utilizing the fact of $\ln x_{1}-x_{1}+1\le 0~\forall ~x_{1}>0.$ Analogously, we have we have

50 $$\begin{aligned} {\mathbb {L}}(-\ln {\textbf{P}}_{{\textbf{h}}}){} & {} =-\frac{\Upsilon _{{\textbf{h}}}}{{\textbf{P}}_{{\textbf{h}}}}+(\vartheta _{1}+\alpha _{1})+\frac{\sigma _{2}^{2}}{2}\nonumber \\ {}{} & {} =-\frac{\Upsilon _{{\textbf{h}}}}{\tilde{{\textbf{P}}_{{\textbf{h}}}}\bar{{\textbf{P}}_{{\textbf{h}}}}}+(\vartheta _{1}+\alpha _{1})+\frac{\sigma _{2}^{2}}{2}\nonumber \\ {}{} & {} \le -\frac{\Upsilon _{{\textbf{h}}}}{\bar{{\textbf{P}}_{{\textbf{h}}}}}\Big (\ln \frac{1}{\tilde{{\textbf{P}}_{{\textbf{h}}}}}\Big )+\alpha _{1}+\vartheta _{1}+\frac{\sigma _{2}^{2}}{2}\nonumber \\ {}{} & {} =\gamma _{{\textbf{h}}}\Big (\frac{{\textbf{Q}}_{{\textbf{r}}}}{{\textbf{N}}_{{\textbf{r}}}}+\frac{\rho _{1}{\textbf{Q}}_{{\textbf{h}}{\textbf{s}}}}{{\textbf{N}}_{{\textbf{h}}}}+\frac{\rho _{2}{\textbf{Q}}_{{\textbf{h}}{\textbf{a}}}}{{\textbf{N}}_{{\textbf{h}}}}+\frac{\rho _{3}{\textbf{G}}_{{\textbf{s}}}}{\Phi _{{\textbf{v}}}}+\frac{\rho _{4}{\textbf{G}}_{{\textbf{a}}}}{\Phi _{{\textbf{v}}}}\Big )\frac{1}{\bar{{\textbf{P}}_{{\textbf{h}}}}}\ln \tilde{{\textbf{P}}_{{\textbf{h}}}}, \end{aligned}$$

51 $$\begin{aligned} {\mathbb {L}}(-\ln {\textbf{Q}}_{{\textbf{h}}{\textbf{a}}}){} & {} =-\frac{\mu \alpha _{1}{\textbf{P}}_{{\textbf{h}}}}{{\textbf{Q}}_{{\textbf{h}}{\textbf{a}}}}+(\vartheta _{1}+\phi _{1})+\frac{\sigma _{3}^{2}}{2}\nonumber \\ {}{} & {} =-\frac{\mu \alpha _{1}\tilde{{\textbf{P}}_{{\textbf{h}}}}\bar{{\textbf{P}}_{{\textbf{h}}}}}{\tilde{{\textbf{Q}}_{{\textbf{h}}{\textbf{a}}}}\bar{{\textbf{Q}}_{{\textbf{h}}{\textbf{a}}}}}+(\vartheta _{1}+\phi _{1})+\frac{\sigma _{3}^{2}}{2}\nonumber \\ {}{} & {} \le -\frac{\mu \alpha _{1}\bar{{\textbf{P}}_{{\textbf{h}}}}}{\bar{{\textbf{Q}}_{{\textbf{h}}{\textbf{a}}}}}\Big (\ln \frac{\bar{{\textbf{P}}_{{\textbf{h}}}}\bar{{\textbf{Q}}_{{\textbf{h}}{\textbf{a}}}}}{\tilde{{\textbf{P}}_{{\textbf{h}}}}\tilde{{\textbf{Q}}_{{\textbf{h}}{\textbf{a}}}}}+1\Big )+\phi _{1}+\vartheta _{1}+\frac{\sigma _{3}^{2}}{2}\nonumber \\ {}{} & {} =-\frac{\mu \alpha _{1}}{\bar{{\textbf{P}}_{{\textbf{h}}}}\bar{{\textbf{Q}}_{{\textbf{h}}{\textbf{a}}}}}\Big (\ln \frac{\bar{{\textbf{P}}_{{\textbf{h}}}}\bar{{\textbf{Q}}_{{\textbf{h}}{\textbf{a}}}}}{\tilde{{\textbf{P}}_{{\textbf{h}}}}\tilde{{\textbf{Q}}_{{\textbf{h}}{\textbf{a}}}}}+1\Big ), \end{aligned}$$

52 $$\begin{aligned} {\mathbb {L}}(-\ln {\textbf{Q}}_{{\textbf{h}}{\textbf{s}}}){} & {} =-\frac{(1-\mu )\alpha _{1}{\textbf{P}}_{{\textbf{h}}}}{{\textbf{Q}}_{{\textbf{h}}{\textbf{s}}}}+(\delta +\vartheta _{1}+\phi _{2})+\frac{\sigma _{4}^{2}}{2}\nonumber \\ {}{} & {} =-\frac{(1-\mu )\alpha _{1}\bar{{\textbf{P}}_{{\textbf{h}}}}\tilde{{\textbf{P}}_{{\textbf{h}}}}}{\tilde{{\textbf{P}}_{{\textbf{h}}}}\tilde{{\textbf{Q}}_{{\textbf{h}}{\textbf{s}}}}\bar{{\textbf{Q}}_{{\textbf{h}}{\textbf{s}}}}}+(\vartheta _{1}+\phi _{1})+\frac{\sigma _{4}^{2}}{2}\nonumber \\ {}{} & {} \le -\frac{(1-\mu )\alpha _{1}}{\bar{{\textbf{P}}_{{\textbf{h}}}}\bar{{\textbf{Q}}_{{\textbf{h}}{\textbf{s}}}}}\Big (\ln \frac{\bar{{\textbf{Q}}_{{\textbf{h}}{\textbf{s}}}}\bar{{\textbf{P}}_{{\textbf{h}}}}}{\tilde{{\textbf{Q}}_{{\textbf{h}}{\textbf{s}}}}\tilde{{\textbf{P}}_{{\textbf{h}}}}}+1\Big )+\phi _{2}+\vartheta _{1}+\delta +\frac{\sigma _{4}^{2}}{2}\nonumber \\ {}{} & {} =-\frac{(1-\mu )\alpha _{1}}{\bar{{\textbf{Q}}_{{\textbf{h}}{\textbf{s}}}}\bar{{\textbf{P}}_{{\textbf{h}}}}}\Big (\ln \frac{\bar{{\textbf{Q}}_{{\textbf{h}}{\textbf{s}}}}\bar{{\textbf{P}}_{{\textbf{h}}}}}{\tilde{{\textbf{P}}_{{\textbf{h}}}}\tilde{{\textbf{Q}}_{{\textbf{h}}{\textbf{s}}}}}+1\Big ), \end{aligned}$$

53 $$\begin{aligned} {\mathbb {L}}(-\ln {\textbf{R}}_{{\textbf{h}}}){} & {} =-\frac{\phi _{1}{\textbf{Q}}_{{\textbf{h}}{\textbf{a}}}}{{\textbf{R}}_{{\textbf{h}}}}-\frac{\phi _{2}{\textbf{Q}}_{{\textbf{h}}{\textbf{s}}}}{{\textbf{R}}_{{\textbf{h}}}}+\vartheta _{1}+\frac{\sigma _{5}^{2}}{2}\nonumber \\ {}{} & {} =-\frac{\phi _{1}\bar{{\textbf{Q}}_{{\textbf{h}}{\textbf{a}}}}\tilde{{\textbf{Q}}_{{\textbf{h}}{\textbf{a}}}}}{\tilde{{\textbf{Q}}_{{\textbf{h}}{\textbf{a}}}}\tilde{{\textbf{R}}_{{\textbf{h}}}}\bar{{\textbf{R}}_{{\textbf{h}}}}}-\frac{\phi _{2}\bar{{\textbf{Q}}_{{\textbf{h}}{\textbf{s}}}}\tilde{{\textbf{Q}}_{{\textbf{h}}{\textbf{s}}}}}{\tilde{{\textbf{Q}}_{{\textbf{h}}{\textbf{s}}}}\tilde{{\textbf{R}}_{{\textbf{h}}}}\bar{{\textbf{R}}_{{\textbf{h}}}}}-\vartheta _{1}+\frac{\sigma _{5}^{2}}{2}\nonumber \\ {}{} & {} \le -\frac{\phi _{1}}{\bar{{\textbf{Q}}_{{\textbf{h}}{\textbf{a}}}}\bar{{\textbf{R}}_{{\textbf{h}}}}}\Big (\ln \frac{\bar{{\textbf{Q}}_{{\textbf{h}}{\textbf{a}}}}\bar{{\textbf{R}}_{{\textbf{h}}}}}{\tilde{{\textbf{Q}}_{{\textbf{h}}{\textbf{a}}}}\tilde{{\textbf{P}}_{{\textbf{h}}}}}+1\Big )-\frac{\phi _{2}}{\bar{{\textbf{Q}}_{{\textbf{h}}{\textbf{s}}}}\bar{{\textbf{R}}_{{\textbf{h}}}}}\Big (\ln \frac{\bar{{\textbf{Q}}_{{\textbf{h}}{\textbf{s}}}}\bar{{\textbf{R}}_{{\textbf{h}}}}}{\tilde{{\textbf{Q}}_{{\textbf{h}}{\textbf{s}}}}\tilde{{\textbf{P}}_{{\textbf{h}}}}}+1\Big )+\vartheta _{1}+\frac{\sigma _{5}^{2}}{2}\nonumber \\ {}{} & {} =-\frac{\phi _{1}}{\bar{{\textbf{Q}}_{{\textbf{h}}{\textbf{a}}}}\bar{{\textbf{R}}_{{\textbf{h}}}}}\Big (\ln \frac{\bar{{\textbf{Q}}_{{\textbf{h}}{\textbf{a}}}}\bar{{\textbf{R}}_{{\textbf{h}}}}}{\tilde{{\textbf{Q}}_{{\textbf{h}}{\textbf{a}}}}\tilde{{\textbf{P}}_{{\textbf{h}}}}}+1\Big )-\frac{\phi _{2}}{\bar{{\textbf{Q}}_{{\textbf{h}}{\textbf{s}}}}\bar{{\textbf{R}}_{{\textbf{h}}}}}\Big (\ln \frac{\bar{{\textbf{Q}}_{{\textbf{h}}{\textbf{s}}}}\bar{{\textbf{R}}_{{\textbf{h}}}}}{\tilde{{\textbf{Q}}_{{\textbf{h}}{\textbf{s}}}}\tilde{{\textbf{P}}_{{\textbf{h}}}}}+1\Big ), \end{aligned}$$

54 $$\begin{aligned} {\mathbb {L}}(-\ln {\textbf{X}}_{{\textbf{r}}}){} & {} =-\frac{\Lambda _{2}}{{\textbf{X}}_{{\textbf{r}}}}+{\Upsilon _{{\textbf{r}}}}+\vartheta _{2}+\upsilon +\frac{\sigma _{6}^{2}}{2}\nonumber \\ {}{} & {} =-\frac{\Lambda _{2}\bar{{\textbf{X}}_{{\textbf{r}}}}}{\tilde{{\textbf{X}}_{{\textbf{r}}}}}+\vartheta _{2}+\upsilon +\Upsilon _{{\textbf{r}}}+\frac{\sigma _{6}^{2}}{2}\nonumber \\ {}{} & {} \le -\frac{\Lambda _{2}}{\bar{{\textbf{X}}_{{\textbf{r}}}}}\Big (\ln \frac{\bar{{\textbf{X}}_{{\textbf{r}}}}}{\tilde{{\textbf{X}}_{{\textbf{r}}}}}+1\Big )+\vartheta _{2}+\upsilon +\Upsilon _{{\textbf{r}}}+\frac{\sigma _{6}^{2}}{2}\nonumber \\ {}{} & {} =-\frac{\Lambda _{2}}{\bar{{\textbf{X}}_{{\textbf{r}}}}}\Big (\ln \frac{\bar{{\textbf{X}}_{{\textbf{r}}}}}{\tilde{{\textbf{X}}_{{\textbf{r}}}}}+1\Big )+\gamma _{{\textbf{r}}}\Big (\frac{{\textbf{Q}}_{{\textbf{r}}}}{{\textbf{N}}_{{\textbf{r}}}}+\frac{\phi _{1}{\textbf{G}}_{{\textbf{s}}}}{\Phi _{{\textbf{v}}}}\Big ), \end{aligned}$$

55 $$\begin{aligned} {\mathbb {L}}(-\ln {\textbf{P}}_{{\textbf{r}}}){} & {} =-\frac{\Upsilon _{{\textbf{r}}}{\textbf{X}}_{{\textbf{r}}}}{{\textbf{P}}_{{\textbf{r}}}}+\alpha _{2}+\upsilon +\vartheta _{2}+\frac{\sigma _{7}^{2}}{2}\nonumber \\ {}{} & {} =-\frac{\Upsilon _{{\textbf{r}}}\bar{{\textbf{X}}_{{\textbf{r}}}}\bar{{\textbf{P}}_{{\textbf{r}}}}}{\tilde{{\textbf{X}}_{{\textbf{r}}}}\tilde{{\textbf{P}}_{{\textbf{r}}}}}+\vartheta _{2}+\upsilon +\alpha _{2}+\frac{\sigma _{7}^{2}}{2}\nonumber \\ {}{} & {} \le -\frac{\Upsilon _{{\textbf{r}}}}{\bar{{\textbf{X}}_{{\textbf{r}}}}\bar{{\textbf{P}}_{{\textbf{r}}}}}\Big (\ln \frac{\bar{{\textbf{X}}_{{\textbf{r}}}}\bar{{\textbf{P}}_{{\textbf{r}}}}}{\tilde{{\textbf{X}}_{{\textbf{r}}}}\bar{{\textbf{P}}_{{\textbf{r}}}}}+1\Big )+\vartheta _{2}+\upsilon +\alpha _{2}+\frac{\sigma _{7}^{2}}{2}\nonumber \\ {}{} & {} =-\gamma _{{\textbf{r}}}\Big (\frac{{\textbf{Q}}_{{\textbf{r}}}}{{\textbf{N}}_{{\textbf{r}}}}+\frac{\phi _{1}{\textbf{G}}_{{\textbf{s}}}}{\Phi _{{\textbf{v}}}}\Big )\frac{1}{\bar{{\textbf{X}}_{{\textbf{r}}}}\bar{{\textbf{P}}_{{\textbf{r}}}}}\Big (\ln \frac{\bar{{\textbf{X}}_{{\textbf{r}}}}\bar{{\textbf{P}}_{{\textbf{r}}}}}{\tilde{{\textbf{P}}_{{\textbf{r}}}}\tilde{{\textbf{X}}_{{\textbf{r}}}}}+1\Big ), \end{aligned}$$

56 $$\begin{aligned} {\mathbb {L}}(-\ln {\textbf{Q}}_{{\textbf{r}}}){} & {} =-\frac{\alpha _{2}{\textbf{P}}_{{\textbf{r}}}}{{\textbf{Q}}_{{\textbf{r}}}}+(\upsilon +\vartheta _{2})+\frac{\sigma _{8}^{2}}{2}\nonumber \\ {}{} & {} =-\frac{\alpha _{2}\bar{{\textbf{P}}_{{\textbf{r}}}}\bar{{\textbf{Q}}_{{\textbf{r}}}}}{\tilde{{\textbf{Q}}_{{\textbf{r}}}}\bar{{\textbf{P}}_{{\textbf{r}}}}}+\vartheta _{2}+\upsilon +\alpha _{2}+\frac{\sigma _{7}^{2}}{2}\nonumber \\ {}{} & {} \le -\frac{\alpha _{2}}{\bar{{\textbf{Q}}_{{\textbf{r}}}}\bar{{\textbf{P}}_{{\textbf{r}}}}}\Big (\ln \frac{\bar{{\textbf{Q}}_{{\textbf{r}}}}\bar{{\textbf{P}}_{{\textbf{r}}}}}{\tilde{{\textbf{Q}}_{{\textbf{r}}}}\bar{{\textbf{P}}_{{\textbf{r}}}}}+1\Big )+\vartheta _{2}+\upsilon +\alpha _{2}+\frac{\sigma _{8}^{2}}{2}\nonumber \\ {}{} & {} =-\frac{\alpha _{2}}{\bar{{\textbf{Q}}_{{\textbf{r}}}}\bar{{\textbf{P}}_{{\textbf{r}}}}}\Big (\ln \frac{\bar{{\textbf{Q}}_{{\textbf{r}}}}\bar{{\textbf{P}}_{{\textbf{r}}}}}{\tilde{{\textbf{Q}}_{{\textbf{r}}}}\bar{{\textbf{P}}_{{\textbf{r}}}}}+1\Big ), \end{aligned}$$

57 $$\begin{aligned} {\mathbb {L}}(-\ln {\textbf{G}}_{{\textbf{s}}}){} & {} =-\frac{\beta _{1}{\textbf{Q}}_{{\textbf{h}}{\textbf{a}}}}{{\textbf{G}}_{{\textbf{s}}}}-\frac{\beta _{2}{\textbf{Q}}_{{\textbf{h}}{\textbf{s}}}}{{\textbf{G}}_{{\textbf{s}}}}-\frac{\beta _{3}{\textbf{Q}}_{{\textbf{r}}}}{{\textbf{G}}_{{\textbf{s}}}}+\xi _{2}+\xi _{3}+\frac{\sigma _{9}^{2}}{2}\nonumber \\ {}{} & {} \le -\frac{\beta _{1}}{\bar{{\textbf{Q}}_{{\textbf{h}}{\textbf{a}}}}\bar{{\textbf{G}}_{{\textbf{s}}}}}\Big (\ln \frac{\bar{{\textbf{Q}}_{{\textbf{h}}{\textbf{a}}}}\bar{{\textbf{G}}_{{\textbf{s}}}}}{\tilde{{\textbf{Q}}_{{\textbf{h}}{\textbf{a}}}}\bar{{\textbf{G}}_{{\textbf{s}}}}}+1\Big )-\frac{\beta _{2}}{\bar{{\textbf{Q}}_{{\textbf{h}}{\textbf{s}}}}\bar{{\textbf{G}}_{{\textbf{s}}}}}\Big (\ln \frac{\bar{{\textbf{Q}}_{{\textbf{h}}{\textbf{s}}}}\bar{{\textbf{G}}_{{\textbf{s}}}}}{\tilde{{\textbf{Q}}_{{\textbf{h}}{\textbf{s}}}}\bar{{\textbf{G}}_{{\textbf{s}}}}}+1\Big )\nonumber \\ {}{} & {} \quad -\frac{\beta _{3}}{\bar{{\textbf{Q}}_{{\textbf{r}}}}\bar{{\textbf{G}}_{{\textbf{s}}}}}\Big (\ln \frac{\bar{{\textbf{Q}}_{{\textbf{r}}}}\bar{{\textbf{G}}_{{\textbf{s}}}}}{\tilde{{\textbf{Q}}_{{\textbf{r}}}}\bar{{\textbf{G}}_{{\textbf{s}}}}}+1\Big )+\xi _{2}+\xi _{3}+\frac{\sigma _{9}^{2}}{2}\nonumber \\ {}{} & {} =-\frac{\beta _{1}}{\bar{{\textbf{Q}}_{{\textbf{h}}{\textbf{a}}}}\bar{{\textbf{G}}_{{\textbf{s}}}}}\Big (\ln \frac{\bar{{\textbf{Q}}_{{\textbf{h}}{\textbf{a}}}}\bar{{\textbf{G}}_{{\textbf{s}}}}}{\tilde{{\textbf{Q}}_{{\textbf{h}}{\textbf{a}}}}\bar{{\textbf{G}}_{{\textbf{s}}}}}+1\Big )-\frac{\beta _{2}}{\bar{{\textbf{Q}}_{{\textbf{h}}{\textbf{s}}}}\bar{{\textbf{G}}_{{\textbf{s}}}}}\Big (\ln \frac{\bar{{\textbf{Q}}_{{\textbf{h}}{\textbf{s}}}}\bar{{\textbf{G}}_{{\textbf{s}}}}}{\tilde{{\textbf{Q}}_{{\textbf{h}}{\textbf{s}}}}\bar{{\textbf{G}}_{{\textbf{s}}}}}+1\Big )\nonumber \\ {}{} & {} \quad -\frac{\beta _{3}}{\bar{{\textbf{Q}}_{{\textbf{r}}}}\bar{{\textbf{G}}_{{\textbf{s}}}}}\Big (\ln \frac{\bar{{\textbf{Q}}_{{\textbf{r}}}}\bar{{\textbf{G}}_{{\textbf{s}}}}}{\tilde{{\textbf{Q}}_{{\textbf{r}}}}\bar{{\textbf{G}}_{{\textbf{s}}}}}+1\Big ), \end{aligned}$$

58 $$\begin{aligned} {\mathbb {L}}(-\ln {\textbf{G}}_{{\textbf{a}}}){} & {} =-\frac{\xi _{3}{\textbf{G}}_{{\textbf{s}}}}{{\textbf{G}}_{{\textbf{a}}}}+\xi _{2}+\frac{\sigma _{10}^{2}}{2}\nonumber \\ {}{} & {} \le -\frac{\xi _{3}}{\bar{{\textbf{G}}_{{\textbf{s}}}}\bar{{\textbf{G}}_{{\textbf{a}}}}}\Big (\ln \frac{\bar{{\textbf{G}}_{{\textbf{s}}}}\bar{{\textbf{G}}_{{\textbf{a}}}}}{\tilde{{\textbf{G}}_{{\textbf{v}}}}\bar{{\textbf{G}}_{{\textbf{s}}}}}+1\Big )+\xi _{2}+\frac{\sigma _{10}^{2}}{2}\nonumber \\ {}{} & {} =-\frac{\xi _{3}}{\bar{{\textbf{G}}_{{\textbf{s}}}}\bar{{\textbf{G}}_{{\textbf{a}}}}}\Big (\ln \frac{\bar{{\textbf{G}}_{{\textbf{s}}}}\bar{{\textbf{G}}_{{\textbf{a}}}}}{\tilde{{\textbf{Q}}_{{\textbf{h}}{\textbf{a}}}}\bar{{\textbf{G}}_{{\textbf{s}}}}}+1\Big )-\frac{\beta _{2}}{\bar{{\textbf{Q}}_{{\textbf{h}}{\textbf{s}}}}\bar{{\textbf{G}}_{{\textbf{s}}}}}\Big (\ln \frac{\bar{{\textbf{Q}}_{{\textbf{h}}{\textbf{s}}}}\bar{{\textbf{G}}_{{\textbf{s}}}}}{\tilde{{\textbf{Q}}_{{\textbf{h}}{\textbf{s}}}}\bar{{\textbf{G}}_{{\textbf{s}}}}}+1\Big ). \end{aligned}$$

Introduce

59 $$\begin{aligned} W_{1}({\tilde{\Theta }}){} & {} =-\ln {\textbf{P}}_{{\textbf{h}}}-c_{1}\ln {\textbf{X}}_{{\textbf{h}}}-c_{2}\ln {\textbf{A}}_{{\textbf{h}}{\textbf{a}}}-c_{3}\ln {\textbf{Q}}_{{\textbf{h}}{\textbf{s}}}-c_{4}\ln {\textbf{R}}_{{\textbf{h}}}-c_{5}\ln {\textbf{X}}_{{\textbf{r}}}-c_{6}\ln {\textbf{P}}_{{\textbf{r}}}\nonumber \\ {}{} & {} \quad -c_{7}\ln {\textbf{Q}}_{{\textbf{r}}}-c_{8}\ln {\textbf{G}}_{{\textbf{s}}}-c_{9}\ln {\textbf{G}}_{{\textbf{a}}}-\frac{c_{1}\gamma _{{\textbf{h}}}}{\phi _{1}+\vartheta _{1}}\ln {\textbf{A}}_{{\textbf{h}}{\textbf{a}}}-\frac{c_{2}\gamma _{{\textbf{h}}}}{\delta +\phi _{2}+\vartheta _{1}}\ln {\textbf{A}}_{{\textbf{h}}{\textbf{s}}}-\frac{c_{3}\alpha _{2}}{\upsilon +\vartheta _{2}},\nonumber \\ \end{aligned}$$

where $c_{\iota }~(\iota =1,...,9)$ are non-negative constants estimated afterwards. Then it implies from (49–58) that

60 $$\begin{aligned} {\mathbb {L}}W_{1}{} & {} \le \gamma _{{\textbf{h}}}\Big (\frac{{\textbf{Q}}_{{\textbf{r}}}}{{\textbf{N}}_{{\textbf{r}}}}+\frac{\rho _{1}{\textbf{Q}}_{{\textbf{h}}{\textbf{s}}}}{{\textbf{N}}_{{\textbf{h}}}}+\frac{\rho _{2}{\textbf{Q}}_{{\textbf{h}}{\textbf{a}}}}{{\textbf{N}}_{{\textbf{h}}}}+\frac{\rho _{3}{\textbf{G}}_{{\textbf{s}}}}{\Phi _{{\textbf{v}}}}+\frac{\rho _{4}{\textbf{G}}_{{\textbf{a}}}}{\Phi _{{\textbf{v}}}}\Big )\frac{1}{\bar{{\textbf{P}}_{{\textbf{h}}}}}\ln \tilde{{\textbf{P}}_{{\textbf{h}}}}+c_{1}\Big \{\frac{\Lambda _{1}}{\bar{{\textbf{X}}_{{\textbf{h}}}}}\Big (\ln \frac{\bar{{\textbf{X}}_{{\textbf{h}}}}}{\tilde{{\textbf{X}}_{{\textbf{h}}}}}+1\Big )+\Upsilon _{{\textbf{h}}}+\vartheta _{1}\Big \}\nonumber \\ {}{} & {} \quad +c_{2}\frac{\mu \alpha _{1}}{\bar{{\textbf{P}}_{{\textbf{h}}}}\bar{{\textbf{Q}}_{{\textbf{h}}{\textbf{a}}}}}\Big (\ln \frac{\bar{{\textbf{P}}_{{\textbf{h}}}}\bar{{\textbf{Q}}_{{\textbf{h}}{\textbf{a}}}}}{\tilde{{\textbf{P}}_{{\textbf{h}}}}\tilde{{\textbf{Q}}_{{\textbf{h}}{\textbf{a}}}}}+1\Big )+c_{3}\frac{(1-\mu )\alpha _{1}}{\bar{{\textbf{Q}}_{{\textbf{h}}{\textbf{s}}}}\bar{{\textbf{P}}_{{\textbf{h}}}}}\Big (\ln \frac{\bar{{\textbf{Q}}_{{\textbf{h}}{\textbf{s}}}}\bar{{\textbf{P}}_{{\textbf{h}}}}}{\tilde{{\textbf{P}}_{{\textbf{h}}}}\tilde{{\textbf{Q}}_{{\textbf{h}}{\textbf{s}}}}}+1\Big )\nonumber \\ {}{} & {} \quad +c_{4}\Big \{\frac{\phi _{1}}{\bar{{\textbf{Q}}_{{\textbf{h}}{\textbf{a}}}}\bar{{\textbf{R}}_{{\textbf{h}}}}}\Big (\ln \frac{\bar{{\textbf{Q}}_{{\textbf{h}}{\textbf{a}}}}\bar{{\textbf{R}}_{{\textbf{h}}}}}{\tilde{{\textbf{Q}}_{{\textbf{h}}{\textbf{a}}}}\tilde{{\textbf{P}}_{{\textbf{h}}}}}+1\Big )-\frac{\phi _{2}}{\bar{{\textbf{Q}}_{{\textbf{h}}{\textbf{s}}}}\bar{{\textbf{R}}_{{\textbf{h}}}}}\Big (\ln \frac{\bar{{\textbf{Q}}_{{\textbf{h}}{\textbf{s}}}}\bar{{\textbf{R}}_{{\textbf{h}}}}}{\tilde{{\textbf{Q}}_{{\textbf{h}}{\textbf{s}}}}\tilde{{\textbf{P}}_{{\textbf{h}}}}}+1\Big )\Big \}+c_{5}\Big \{\frac{\Lambda _{2}}{\bar{{\textbf{X}}_{{\textbf{r}}}}}\Big (\ln \frac{\bar{{\textbf{X}}_{{\textbf{r}}}}}{\tilde{{\textbf{X}}_{{\textbf{r}}}}}+1\Big )\nonumber \\ {}{} & {} \quad +\gamma _{{\textbf{r}}}\Big (\frac{{\textbf{Q}}_{{\textbf{r}}}}{{\textbf{N}}_{{\textbf{r}}}}+\frac{\phi _{1}{\textbf{G}}_{{\textbf{s}}}}{\Phi _{{\textbf{v}}}}\Big )\Big \}+c_{6}\gamma _{{\textbf{r}}}\Big (\frac{{\textbf{Q}}_{{\textbf{r}}}}{{\textbf{N}}_{{\textbf{r}}}}+\frac{\phi _{1}{\textbf{G}}_{{\textbf{s}}}}{\Phi _{{\textbf{v}}}}\Big )\frac{1}{\bar{{\textbf{X}}_{{\textbf{r}}}}\bar{{\textbf{P}}_{{\textbf{r}}}}}\Big (\ln \frac{\bar{{\textbf{X}}_{{\textbf{r}}}}\bar{{\textbf{P}}_{{\textbf{r}}}}}{\tilde{{\textbf{P}}_{{\textbf{r}}}}\tilde{{\textbf{X}}_{{\textbf{r}}}}}+1\Big )\nonumber \\ {}{} & {} \quad +c_{7}\frac{\alpha _{2}}{\bar{{\textbf{Q}}_{{\textbf{r}}}}\bar{{\textbf{P}}_{{\textbf{r}}}}}\Big (\ln \frac{\bar{{\textbf{Q}}_{{\textbf{r}}}}\bar{{\textbf{P}}_{{\textbf{r}}}}}{\tilde{{\textbf{Q}}_{{\textbf{r}}}}\bar{{\textbf{P}}_{{\textbf{r}}}}}+1\Big )+c_{8}\Big \{\frac{\beta _{1}}{\bar{{\textbf{Q}}_{{\textbf{h}}{\textbf{a}}}}\bar{{\textbf{G}}_{{\textbf{s}}}}}\Big (\ln \frac{\bar{{\textbf{Q}}_{{\textbf{h}}{\textbf{a}}}}\bar{{\textbf{G}}_{{\textbf{s}}}}}{\tilde{{\textbf{Q}}_{{\textbf{h}}{\textbf{a}}}}\bar{{\textbf{G}}_{{\textbf{s}}}}}+1\Big )-\frac{\beta _{2}}{\bar{{\textbf{Q}}_{{\textbf{h}}{\textbf{s}}}}\bar{{\textbf{G}}_{{\textbf{s}}}}}\Big (\ln \frac{\bar{{\textbf{Q}}_{{\textbf{h}}{\textbf{s}}}}\bar{{\textbf{G}}_{{\textbf{s}}}}}{\tilde{{\textbf{Q}}_{{\textbf{h}}{\textbf{s}}}}\bar{{\textbf{G}}_{{\textbf{s}}}}}+1\Big )\nonumber \\ {}{} & {} \quad -\frac{\beta _{3}}{\bar{{\textbf{Q}}_{{\textbf{r}}}}\bar{{\textbf{G}}_{{\textbf{s}}}}}\Big (\ln \frac{\bar{{\textbf{Q}}_{{\textbf{r}}}}\bar{{\textbf{G}}_{{\textbf{s}}}}}{\tilde{{\textbf{Q}}_{{\textbf{r}}}}\bar{{\textbf{G}}_{{\textbf{s}}}}}+1\Big )\Big \}+c_{9}\Big \{\frac{\xi _{3}}{\bar{{\textbf{G}}_{{\textbf{s}}}}\bar{{\textbf{G}}_{{\textbf{a}}}}}\Big (\ln \frac{\bar{{\textbf{G}}_{{\textbf{s}}}}\bar{{\textbf{G}}_{{\textbf{a}}}}}{\tilde{{\textbf{Q}}_{{\textbf{h}}{\textbf{a}}}}\bar{{\textbf{G}}_{{\textbf{s}}}}}+1\Big )-\frac{\beta _{2}}{\bar{{\textbf{Q}}_{{\textbf{h}}{\textbf{s}}}}\bar{{\textbf{G}}_{{\textbf{s}}}}}\Big (\ln \frac{\bar{{\textbf{Q}}_{{\textbf{h}}{\textbf{s}}}}\bar{{\textbf{G}}_{{\textbf{s}}}}}{\tilde{{\textbf{Q}}_{{\textbf{h}}{\textbf{s}}}}\bar{{\textbf{G}}_{{\textbf{s}}}}}+1\Big )\Big \}\nonumber \\ {}{} & {} \quad +\frac{c_{1}\gamma _{{\textbf{h}}}\mu \alpha _{1}}{\vartheta _{1}+\phi _{1}}{\textbf{P}}_{{\textbf{h}}}-c_{1}\gamma _{{\textbf{h}}}{\textbf{Q}}_{{\textbf{h}}{\textbf{a}}}-\frac{c_{2}\gamma _{{\textbf{h}}}(1-\mu )\alpha _{1}}{\delta +\phi _{2}+\vartheta _{1}}{\textbf{P}}_{{\textbf{h}}}-c_{2}\gamma _{{\textbf{h}}}{\textbf{Q}}_{{\textbf{h}}{\textbf{s}}}+\frac{c_{3}\alpha _{2}}{\upsilon +\vartheta _{2}}{\textbf{P}}_{{\textbf{r}}}-c_{3}\alpha _{3}{\textbf{Q}}_{{\textbf{h}}}. \end{aligned}$$

Simple computation yields

$$\begin{aligned} {\left\{ \begin{array}{ll} \frac{c_{1}\Lambda _{1}}{\bar{{\textbf{X}}_{{\textbf{h}}}}}-\Upsilon _{{\textbf{h}}}=0,\\ \frac{c_{2}\mu \alpha _{1}}{\bar{{\textbf{Q}}_{{\textbf{h}}{\textbf{a}}}}}-\phi _{1}\bar{{\textbf{R}}_{{\textbf{h}}}}=0,\\ \frac{c_{3}(1-\mu )\alpha _{1}}{\bar{{\textbf{Q}}_{{\textbf{h}}{\textbf{s}}}}}-\phi _{2}\bar{{\textbf{Q}}_{{\textbf{h}}{\textbf{s}}}}=0,\\ \frac{c_{4}\phi _{1}}{\bar{{\textbf{R}}_{{\textbf{h}}}}}-(\phi _{2}+\beta _{2})\bar{{\textbf{G}}_{{\textbf{s}}}}\bar{{\textbf{Q}}_{{\textbf{h}}{\textbf{s}}}}=0,\\ \frac{c_{5}\Lambda _{2}}{\bar{{\textbf{X}}_{{\textbf{r}}}}}+\gamma _{{\textbf{r}}}\bar{{\textbf{P}}_{{\textbf{h}}}}\bar{{\textbf{X}}_{{\textbf{r}}}}=0,\\ \frac{c_{6}\gamma _{{\textbf{r}}}}{\bar{{\textbf{X}}_{{\textbf{r}}}}}+\gamma _{{\textbf{r}}}\bar{{\textbf{P}}_{{\textbf{r}}}}\bar{{\textbf{X}}_{{\textbf{r}}}}=0,\\ \frac{c_{7}\alpha _{2}}{\bar{{\textbf{Q}}_{{\textbf{r}}}}}+\beta _{3}\bar{{\textbf{G}}_{{\textbf{s}}}}\bar{{\textbf{Q}}_{{\textbf{r}}}}=0,\\ \frac{c_{8}\beta _{1}}{\bar{{\textbf{G}}_{{\textbf{s}}}}}+\beta _{3}\bar{{\textbf{Q}}}_{{\textbf{h}}{\textbf{a}}}\bar{{\textbf{G}}_{{\textbf{s}}}}=0,\\ \frac{c_{9}\xi _{3}}{\bar{{\textbf{G}}_{{\textbf{a}}}}}+\beta _{3}\bar{{\textbf{Q}}}_{{\textbf{h}}{\textbf{s}}}\bar{{\textbf{G}}_{{\textbf{a}}}}=0, \end{array}\right. }\implies {\left\{ \begin{array}{ll} c_{1}=\frac{\Upsilon _{{\textbf{h}}}\bar{{\textbf{X}}_{{\textbf{h}}}}}{\Lambda _{1}},\\ c_{2}=\frac{\phi _{1}\bar{{\textbf{R}}_{{\textbf{h}}}}\bar{{\textbf{Q}}_{{\textbf{h}}{\textbf{a}}}}}{\mu \alpha _{1}},\\ c_{3}=\frac{\phi _{2}\bar{{\textbf{Q}}_{{\textbf{h}}{\textbf{s}}}}}{(1-\mu )\alpha _{1}},\\ c_{4}=\frac{(\phi _{2}+\beta _{2})\bar{{\textbf{G}}_{{\textbf{s}}}}\bar{{\textbf{Q}}_{{\textbf{h}}{\textbf{s}}}}\bar{{\textbf{R}}_{{\textbf{h}}}}}{\phi _{1}},\\ c_{5}=\frac{\gamma _{{\textbf{r}}}\bar{{{\textbf{P}}_{{\textbf{h}}}}}\bar{{\textbf{X}}_{{\textbf{r}}}^{2}}}{\Lambda _{2}},\\ c_{6}=\frac{\Lambda _{2}\bar{{{\textbf{P}}_{{\textbf{r}}}}}\bar{{\textbf{X}}_{{\textbf{r}}}^{2}}}{\Lambda _{2}},\\ c_{7}=\frac{\beta _{3}\bar{{{\textbf{G}}_{{\textbf{s}}}}^{2}}\bar{{\textbf{Q}}_{{\textbf{r}}}}}{\alpha _{2}},\\ c_{8}=\frac{\beta _{3}\bar{{{\textbf{G}}_{{\textbf{s}}}}}\bar{{\textbf{Q}}_{{\textbf{h}}{\textbf{a}}}}}{\beta _{1}},\\ c_{9}=\frac{\beta _{3}\bar{{{\textbf{G}}_{{\textbf{a}}}}^{2}}\bar{{\textbf{Q}}_{{\textbf{h}}{\textbf{s}}}}}{\xi _{2}}. \end{array}\right. } \end{aligned}$$

Thus,

61 $$\begin{aligned} {\mathbb {L}}W_{1}{} & {} \le -{\Upsilon 
_{{\textbf{h}}}\bar{{\textbf{X}}_{{\textbf{h}}}}}+\alpha _{1}+\vartheta _{1}+\frac{\sigma _{2}^{2}}{2}+\Big (\frac{c_{1}\gamma _{{\textbf{h}}}\mu \alpha _{1}}{\vartheta _{1}+\phi _{1}}+\frac{c_{2}\gamma _{{\textbf{h}}}(1-\mu )\alpha _{1}}{\vartheta _{1}+\phi _{2}+\delta }\Big ){\textbf{P}}_{{\textbf{h}}}\nonumber \\ {}{} & {} =-\Big (\alpha _{1}+\vartheta _{1}+\frac{\sigma _{2}^{2}}{2}\Big )(\Re _{0}^{s}-1)+\Big (\frac{c_{1}\gamma _{{\textbf{h}}}\mu \alpha _{1}}{\vartheta _{1}+\phi _{1}}+\frac{c_{2}\gamma _{{\textbf{h}}}(1-\mu )\alpha _{1}}{\vartheta _{1}+\phi _{2}+\delta }\Big ){\textbf{P}}_{{\textbf{h}}}, \end{aligned}$$

where

62 $$\begin{aligned} \Re _{0}^{s}{} & {} :=\frac{\Upsilon _{{\textbf{h}}}\bar{{\textbf{X}}_{{\textbf{h}}}}}{\alpha _{1}+\vartheta _{1}+\frac{\sigma _{2}^{2}}{2}}\nonumber \\ {}{} & {} =\frac{\Lambda _{1}\gamma _{{\textbf{h}}}\mu \alpha _{1}\Upsilon _{{\textbf{h}}}}{(\alpha _{1}+\vartheta _{1}+\frac{\sigma _{2}^{2}}{2})(\vartheta _{1}+\phi _{1}+\frac{\sigma _{3}^{2}}{2})(\vartheta _{1}+\frac{\sigma _{1}^{2}}{2})}+\frac{\Lambda _{1}\gamma _{{\textbf{h}}}(1-\mu )\alpha _{1}\Upsilon _{{\textbf{h}}}}{(\alpha _{1}+\vartheta _{1}+\frac{\sigma _{2}^{2}}{2})(\vartheta _{1}+\phi _{2}+\frac{\sigma _{4}^{2}}{2})(\vartheta _{1}+\frac{\sigma _{1}^{2}}{2})}. \end{aligned}$$

Furthermore, we introduce the functions

63 $$\begin{aligned}{} & {} W_{2}({\textbf{X}}_{{\textbf{h}}})=-\ln {\textbf{X}}_{{\textbf{h}}},~~W_{3}({\textbf{Q}}_{{\textbf{h}}{\textbf{a}}})=-\ln {\textbf{Q}}_{{\textbf{h}}{\textbf{a}}},~~W_{3}({\textbf{Q}}_{{\textbf{h}}{\textbf{s}}})=-\ln {\textbf{Q}}_{{\textbf{h}}{\textbf{s}}},~~W_{4}({\textbf{R}}_{{\textbf{h}}})=-\ln {\textbf{R}}_{{\textbf{h}}},\nonumber \\ {}{} & {} W_{5}({\textbf{X}}_{{\textbf{r}}})=-\ln {\textbf{X}}_{{\textbf{r}}},~~W_{6}({\textbf{P}}_{{\textbf{r}}})=-\ln {\textbf{P}}_{{\textbf{r}}},~~W_{7}({\textbf{Q}}_{{\textbf{r}}})=-\ln {\textbf{Q}}_{{\textbf{r}}},~~ W_{8}({\textbf{G}}_{{\textbf{s}}})=-\ln {\textbf{G}}_{{\textbf{s}}},\nonumber \\ {}{} & {} W_{9}({\textbf{G}}_{{\textbf{a}}})=-\ln {\textbf{G}}_{{\textbf{a}}},~~W_{10}({\tilde{\Theta }})=\frac{1}{\lambda +1}\sum \limits _{\iota =1}^{10}{\tilde{\Theta }}_{\iota }^{\lambda +1},~~\end{aligned}$$

where $\lambda \in \big (0,2\vartheta _{1}/(\sigma _{1}^{2}\vee \sigma _{2}^{2}\vee \sigma _{3}^{2}\vee \sigma _{4}^{2}\vee \sigma _{5}^{2}\vee \sigma _{6}^{2}\vee \sigma _{7}^{2}\vee \sigma _{8}^{2}\vee \sigma _{9}^{2}\vee \sigma _{10}^{2})\big )$ is sufficiently small constant. Utilizing the Itô technique to $W_{2},W_{3},W_{4},W_{5},W_{6},W_{7},W_{8},W_{9}$ and $W_{10},$ respectively, we have

64 $$\begin{aligned}{} & {} {\mathbb {L}}W_{2}=-\frac{\Lambda _{1}}{{\textbf{X}}_{{\textbf{h}}}}+\Upsilon _{{\textbf{h}}}+\vartheta _{1}+\frac{\sigma _{1}^{2}}{2},~~~~{\mathbb {L}}W_{3}=-\frac{\mu \alpha _{1}{\textbf{P}}}{{\textbf{Q}}_{{\textbf{h}}{\textbf{a}}}}+(\vartheta _{1}+\phi _{1})+\frac{\sigma _{3}^{2}}{2},\nonumber \\ {}{} & {} {\mathbb {L}}W_{3}=-\frac{(1-\mu )\alpha _{1}{\textbf{P}}_{{\textbf{h}}}}{{\textbf{Q}}_{{\textbf{h}}{\textbf{s}}}}+(\vartheta _{1}+\phi _{2}+\delta )+\frac{\sigma _{4}^{2}}{2},~~~~{\mathbb {L}}W_{4}=-\frac{\phi _{1}{\textbf{Q}}_{{\textbf{h}}{\textbf{a}}}}{{\textbf{R}}_{{\textbf{h}}}}-\frac{\phi _{2}{\textbf{Q}}_{{\textbf{h}}{\textbf{s}}}}{{\textbf{R}}_{{\textbf{h}}}}+\vartheta _{1}+\frac{\sigma _{5}^{2}}{2}, \nonumber \\ {}{} & {} {\mathbb {L}}W_{5}=-\frac{\Lambda _{2}}{{\textbf{X}}_{{\textbf{r}}}}+\Upsilon _{{\textbf{r}}}+(\vartheta _{2}+\upsilon )+\frac{\sigma _{5}^{2}}{2},~~~~{\mathbb {L}}W_{6}=-\frac{\Upsilon _{{\textbf{r}}}{\textbf{X}}_{{\textbf{r}}}}{{\textbf{P}}_{{\textbf{r}}}}+(\alpha _{2}+\upsilon +\vartheta _{2})+\frac{\sigma _{6}^{2}}{2}, \nonumber \\ {}{} & {} {\mathbb {L}}W_{7}=-\frac{\alpha _{2}{\textbf{P}}_{{\textbf{r}}}}{{\textbf{Q}}_{{\textbf{r}}}}+(\vartheta _{2}+\upsilon )+\frac{\sigma _{7}^{2}}{2},~~~~{\mathbb {L}}W_{8}=-\frac{\beta _{1}{\textbf{Q}}_{{\textbf{h}}{\textbf{a}}}}{{\textbf{G}}_{{\textbf{s}}}}-\frac{\beta _{2}{\textbf{Q}}_{{\textbf{h}}{\textbf{s}}}}{{\textbf{G}}_{{\textbf{s}}}}-\frac{\beta _{3}{\textbf{Q}}_{{\textbf{r}}}}{{\textbf{G}}_{{\textbf{s}}}}+(\xi _{2}+\xi _{3})+\frac{\sigma _{8}^{2}}{2},\nonumber \\ {}{} & {} {\mathbb {L}}W_{9}=-\frac{\xi _{3}{\textbf{G}}_{{\textbf{s}}}}{{\textbf{G}}_{{\textbf{a}}}}+\xi _{2}+\frac{\sigma _{9}^{2}}{2} \end{aligned}$$

and

65 $$\begin{aligned} {\mathbb {L}}W_{10}{} & {} =\big ({\textbf{X}}_{{\textbf{h}}}+{\textbf{P}}_{{\textbf{h}}}+{\textbf{Q}}_{{\textbf{h}}{\textbf{a}}}+{\textbf{Q}}_{{\textbf{h}}{\textbf{s}}}+{\textbf{R}}_{{\textbf{h}}}+{\textbf{X}}_{{\textbf{r}}}+{\textbf{P}}_{{\textbf{r}}}+{\textbf{Q}}_{{\textbf{r}}}+{\textbf{G}}_{{\textbf{s}}}+{\textbf{G}}_{{\textbf{a}}}\big )^{\lambda }\nonumber \\ {}{} & {} \quad \times \big [\Lambda _{1}-\mu ({\textbf{X}}_{{\textbf{h}}}+{\textbf{P}}_{{\textbf{h}}}+{\textbf{Q}}_{{\textbf{h}}{\textbf{a}}}+{\textbf{Q}}_{{\textbf{h}}{\textbf{s}}}+{\textbf{R}}_{{\textbf{h}}}+{\textbf{X}}_{{\textbf{r}}}+{\textbf{P}}_{{\textbf{r}}}+{\textbf{Q}}_{{\textbf{r}}}+{\textbf{G}}_{{\textbf{s}}}+{\textbf{G}}_{{\textbf{a}}})-\phi _{1}{\textbf{Q}}_{{\textbf{h}}{\textbf{a}}}\nonumber \\ {}{} & {} \quad -(\delta +\phi _{1}){\textbf{Q}}_{{\textbf{h}}{\textbf{s}}}-\upsilon {\textbf{Q}}_{{\textbf{r}}}\big ]+\frac{\lambda }{2}({\textbf{X}}_{{\textbf{h}}}+{\textbf{P}}_{{\textbf{h}}}+{\textbf{Q}}_{{\textbf{h}}{\textbf{a}}}+{\textbf{Q}}_{{\textbf{h}}{\textbf{s}}}+{\textbf{R}}_{{\textbf{h}}}+{\textbf{X}}_{{\textbf{r}}}+{\textbf{P}}_{{\textbf{r}}}+{\textbf{Q}}_{{\textbf{r}}}+{\textbf{G}}_{{\textbf{s}}}\nonumber \\ {}{} & {} \quad +{\textbf{G}}_{{\textbf{a}}})^{\lambda -1}\times (\sigma _{1}^{2}{\textbf{X}}_{{\textbf{h}}}+\sigma _{2}^{2}{\textbf{P}}_{{\textbf{h}}}+\sigma _{3}^{2}{\textbf{Q}}_{{\textbf{h}}{\textbf{a}}}+\sigma _{4}^{2}{\textbf{Q}}_{{\textbf{h}}{\textbf{s}}}+\sigma _{5}^{2}{\textbf{R}}_{{\textbf{h}}}+\sigma _{6}^{2}{\textbf{X}}_{{\textbf{r}}}+\sigma _{7}^{2}{\textbf{P}}_{{\textbf{r}}}+\sigma _{8}^{2}{\textbf{Q}}_{{\textbf{r}}}\nonumber \\ {}{} & {} \quad +\sigma _{9}^{2}{\textbf{G}}_{{\textbf{s}}}+\sigma _{10}^{2}{\textbf{G}}_{{\textbf{a}}})\nonumber \\ {}{} & {} \le \Lambda _{1}\big ({\textbf{X}}_{{\textbf{h}}}+{\textbf{P}}_{{\textbf{h}}}+{\textbf{Q}}_{{\textbf{h}}{\textbf{a}}}+{\textbf{Q}}_{{\textbf{h}}{\textbf{s}}}+{\textbf{R}}_{{\textbf{h}}}+{\textbf{X}}_{{\textbf{r}}}+{\textbf{P}}_{{\textbf{r}}}+{\textbf{Q}}_{{\textbf{r}}}+{\textbf{G}}_{{\textbf{s}}}+{\textbf{G}}_{{\textbf{a}}}\big )^{\lambda }\nonumber \\ {}{} & {} \quad -\vartheta _{1}\big ({\textbf{X}}_{{\textbf{h}}}+{\textbf{P}}_{{\textbf{h}}}+{\textbf{Q}}_{{\textbf{h}}{\textbf{a}}}+{\textbf{Q}}_{{\textbf{h}}{\textbf{s}}}+{\textbf{R}}_{{\textbf{h}}}+{\textbf{X}}_{{\textbf{r}}}+{\textbf{P}}_{{\textbf{r}}}+{\textbf{Q}}_{{\textbf{r}}}+{\textbf{G}}_{{\textbf{s}}}+{\textbf{G}}_{{\textbf{a}}}\big )^{\lambda +1}\nonumber \\ {}{} & {} \quad +\frac{\lambda }{2}\big ({\textbf{X}}_{{\textbf{h}}}+{\textbf{P}}_{{\textbf{h}}}+{\textbf{Q}}_{{\textbf{h}}{\textbf{a}}}+{\textbf{Q}}_{{\textbf{h}}{\textbf{s}}}+{\textbf{R}}_{{\textbf{h}}}+{\textbf{X}}_{{\textbf{r}}}+{\textbf{P}}_{{\textbf{r}}}+{\textbf{Q}}_{{\textbf{r}}}+{\textbf{G}}_{{\textbf{s}}}+{\textbf{G}}_{{\textbf{a}}}\big )^{\lambda +1}\nonumber \\ {}{} & {} \quad \times (\sigma _{1}^{2}\vee \sigma _{2}^{2}\vee \sigma _{3}^{2}\vee \sigma _{4}^{2}\vee \sigma _{5}^{2}\vee \sigma _{6}^{2}\vee \sigma _{7}^{2}\vee \sigma _{8}^{2}\vee \sigma _{9}^{2}\vee \sigma _{10}^{2})\nonumber \\ {}{} & {} ={\mathcal {X}}_{1}-\frac{1}{2}\Big (\vartheta _{1}-\frac{\lambda }{2}(\sigma _{1}^{2}\vee \sigma _{2}^{2}\vee \sigma _{3}^{2}\vee \sigma _{4}^{2}\vee \sigma _{5}^{2}\vee \sigma _{6}^{2}\vee \sigma _{7}^{2}\vee \sigma _{8}^{2}\vee \sigma _{9}^{2}\vee \sigma _{10}^{2}\Big )\nonumber \\ {}{} & {} \quad \times \big ({\textbf{X}}_{{\textbf{h}}}+{\textbf{P}}_{{\textbf{h}}}+{\textbf{Q}}_{{\textbf{h}}{\textbf{a}}}+{\textbf{Q}}_{{\textbf{h}}{\textbf{s}}}+{\textbf{R}}_{{\textbf{h}}}+{\textbf{X}}_{{\textbf{r}}}+{\textbf{P}}_{{\textbf{r}}}+{\textbf{Q}}_{{\textbf{r}}}+{\textbf{G}}_{{\textbf{s}}}+{\textbf{G}}_{{\textbf{a}}}\big )^{\lambda +1}\nonumber \\ {}{} & {} \le {\mathcal {X}}_{1}-\frac{1}{2}\Big (\vartheta _{1}-\frac{\lambda }{2}(\sigma _{1}^{2}\vee \sigma _{2}^{2}\vee \sigma _{3}^{2}\vee \sigma _{4}^{2}\vee \sigma _{5}^{2}\vee \sigma _{6}^{2}\vee \sigma _{7}^{2}\vee \sigma _{8}^{2}\vee \sigma _{9}^{2}\vee \sigma _{10}^{2}\Big )\nonumber \\ {}{} & {} \quad \times \big ({\textbf{X}}_{{\textbf{h}}}^{\lambda +1}+{\textbf{P}}_{{\textbf{h}}}^{\lambda +1}+{\textbf{Q}}_{{\textbf{h}}{\textbf{a}}}^{\lambda +1}+{\textbf{Q}}_{{\textbf{h}}{\textbf{s}}}^{\lambda +1}+{\textbf{R}}_{{\textbf{h}}}^{\lambda +1}+{\textbf{X}}_{{\textbf{r}}}^{\lambda +1}+{\textbf{P}}_{{\textbf{r}}}^{\lambda +1}+{\textbf{Q}}_{{\textbf{r}}}^{\lambda +1}+{\textbf{G}}_{{\textbf{s}}}^{\lambda +1}+{\textbf{G}}_{{\textbf{a}}}^{\lambda +1}\big )\nonumber \\ {}{} & {} ={\mathcal {X}}_{1}-\frac{{\mathcal {X}}_{2}}{2}\Big ({\textbf{X}}_{{\textbf{h}}}^{\lambda +1}+{\textbf{P}}_{{\textbf{h}}}^{\lambda +1}+{\textbf{Q}}_{{\textbf{h}}{\textbf{a}}}^{\lambda +1}+{\textbf{Q}}_{{\textbf{h}}{\textbf{s}}}^{\lambda +1}+{\textbf{R}}_{{\textbf{h}}}^{\lambda +1}+{\textbf{X}}_{{\textbf{r}}}^{\lambda +1}+{\textbf{P}}_{{\textbf{r}}}^{\lambda +1}+{\textbf{Q}}_{{\textbf{r}}}^{\lambda +1}+{\textbf{G}}_{{\textbf{s}}}^{\lambda 
+1}+{\textbf{G}}_{{\textbf{a}}}^{\lambda +1}\Big ), \end{aligned}$$

where $${\mathcal {X}}_{1}:=\sup \limits _{{\tilde{\Theta }}^{{\tilde{T}}}\in \Re _{+}^{10}}\Big \{\Lambda _{1}({\textbf{X}}_{{\textbf{h}}}+{\textbf{P}}_{{\textbf{h}}}+{\textbf{Q}}_{{\textbf{h}}{\textbf{a}}}+{\textbf{Q}}_{{\textbf{h}}{\textbf{s}}}+{\textbf{R}}_{{\textbf{h}}}+{\textbf{X}}_{{\textbf{r}}}+{\textbf{P}}_{{\textbf{r}}}+{\textbf{Q}}_{{\textbf{r}}}+{\textbf{G}}_{{\textbf{s}}}+{\textbf{G}}_{{\textbf{a}}})^{\lambda }-\frac{{\mathcal {X}}_{2}}{2}({\textbf{X}}_{{\textbf{h}}}+{\textbf{P}}_{{\textbf{h}}}+{\textbf{Q}}_{{\textbf{h}}{\textbf{a}}}+{\textbf{Q}}_{{\textbf{h}}{\textbf{s}}}+{\textbf{R}}_{{\textbf{h}}}+{\textbf{X}}_{{\textbf{r}}}+{\textbf{P}}_{{\textbf{r}}}+{\textbf{Q}}_{{\textbf{r}}}+{\textbf{G}}_{{\textbf{s}}}+{\textbf{G}}_{{\textbf{a}}})^{\lambda +1}\Big \}<\infty$$ and $${\mathcal {X}}_{2}:=\vartheta _{1}-\frac{\lambda }{2}\Big (\vartheta _{1}-\frac{\lambda }{2}(\sigma _{1}^{2}\vee \sigma _{2}^{2}\vee \sigma _{3}^{2}\vee \sigma _{4}^{2}\vee \sigma _{5}^{2}\vee \sigma _{6}^{2}\vee \sigma _{7}^{2}\vee \sigma _{8}^{2}\vee \sigma _{9}^{2}\vee \sigma _{10}^{2}\Big ).$$

Introducing a $\bar{C}^{2}$-function $\bar{W}:\Re _{+}^{10}\mapsto \Re$ in the subsequent form

66 $$\begin{aligned} W_{1}({\tilde{\Theta }}){} & {} ={\mathbb {Q}}W_{1}({\tilde{\Theta }})+W_{2}({\textbf{X}}_{{\textbf{h}}})+W_{3}({\textbf{Q}}_{{\textbf{h}}{\textbf{a}}})+W_{4}({\textbf{Q}}_{{\textbf{h}}{\textbf{s}}})+W_{5}({\textbf{R}}_{{\textbf{h}}})+W_{6}({\textbf{X}}_{{\textbf{r}}})+W_{7}({\textbf{P}}_{{\textbf{r}}})+W_{8}({\textbf{Q}}_{{\textbf{r}}})\nonumber \\ {}{} & {} \quad +W_{9}({\textbf{G}}_{{\textbf{s}}})+W_{10}({\textbf{G}}_{{\textbf{a}}}), \end{aligned}$$

where ${\mathbb {Q}}$ is a sufficiently large non-negative quantity fulfilling the criteria

67 $$\begin{aligned} {\mathbb {Q}}\Big (\vartheta _{1}+\alpha _{1}+\frac{\sigma _{1}^{2}}{2}\Big )(\Re _{0}^{s}-1)+{\mathcal {X}}_{3}\le -2 \end{aligned}$$

and

68 $$\begin{aligned} {\mathcal {X}}_{3}{} & {} :=\sup \limits _{{\tilde{\Theta }}^{{\tilde{T}}}\in \Re _{+}^{10}}\bigg \{-\frac{{\mathcal {X}}_{2}}{4}\big ({\textbf{X}}_{{\textbf{h}}}^{\lambda +1}+{\textbf{P}}_{{\textbf{h}}}^{\lambda +1}+{\textbf{Q}}_{{\textbf{h}}{\textbf{a}}}^{\lambda +1}+{\textbf{Q}}_{{\textbf{h}}{\textbf{s}}}^{\lambda +1}+{\textbf{R}}_{{\textbf{h}}}^{\lambda +1}+{\textbf{X}}_{{\textbf{r}}}^{\lambda +1}+{\textbf{P}}_{{\textbf{r}}}^{\lambda +1}+{\textbf{Q}}_{{\textbf{r}}}^{\lambda +1}+{\textbf{G}}_{{\textbf{s}}}^{\lambda +1}+{\textbf{G}}_{{\textbf{a}}}^{\lambda +1}\big )\nonumber \\ {}{} & {} \quad +{\mathcal {X}}_{1}+\Lambda _{1}+5\vartheta _{1}+3\vartheta _{2}+\Upsilon _{{\textbf{h}}}{\textbf{X}}_{{\textbf{h}}}+\Upsilon _{{\textbf{h}}}{\textbf{P}}_{{\textbf{h}}}+\phi _{1}+\phi _{2}+\delta +\upsilon +\alpha _{2}+\xi _{2}+\xi _{3}+\frac{1}{2}(\sigma _{1}^{2}\vee \sigma _{3}^{2}\vee \sigma _{4}^{2}\nonumber \\ {}{} & {} \quad \vee \sigma _{5}^{2}\vee \sigma _{6}^{2}\vee \sigma _{7}^{2}\vee \sigma _{8}^{2}\vee \sigma _{9}^{2}\vee \sigma _{10}^{2})\bigg \}<\infty . \end{aligned}$$

Furthermore, $\bar{W}({\tilde{\Theta }})$ is not only continuous, nevertheless it tends to as $({\tilde{\Theta }})^{{\tilde{T}}}$ arrives at the boundary of $\Re _{+}^{10}$. As a result, it must possess a lower bound that reaches it at a point $({\tilde{\Theta }}^{0})$ in the interior of $\Re _{+}^{10}$. Then we establish a $\bar{C}^{2}$-function $\bar{W}:\Re _{+}^{10}\mapsto \Re _{+}$ as follows

69 $$\begin{aligned} {\tilde{W}}({\tilde{\Theta }}){} & {} =\bar{W}({\tilde{\Theta }})-\bar{W}({\tilde{\Theta }}^{0})\nonumber \\ {}{} & {} ={\mathbb {Q}}W_{1}({\tilde{\Theta }})+W_{2}({\textbf{X}}_{{\textbf{h}}})+W_{3}({\textbf{Q}}_{{\textbf{h}}{\textbf{a}}})+W_{3}({\textbf{Q}}_{{\textbf{h}}{\textbf{s}}})+W_{4}({\textbf{R}}_{{\textbf{h}}})+W_{5}({\textbf{X}}_{{\textbf{r}}})+W_{6}({\textbf{P}}_{{\textbf{r}}})+W_{7}({\textbf{Q}}_{{\textbf{r}}})\nonumber \\ {}{} & {} \quad +W_{8}({\textbf{G}}_{{\textbf{s}}})+W_{9}({\textbf{G}}_{{\textbf{a}}})-\bar{W}({\tilde{\Theta }}^{0}) \end{aligned}$$

In view of (61–65), we have

$$\begin{aligned} {\mathbb {L}}{\tilde{W}}{} & {} \le {\mathbb {Q}}\Big (\vartheta _{1}+\phi _{1}+\frac{\sigma _{1}^{2}}{2}\Big )(\Re _{0}^{s}-1)+{\mathbb {Q}}\bigg (\frac{c_{1}\gamma _{{\textbf{h}}}\mu \alpha _{1}}{\vartheta _{1}+\phi _{1}}+\frac{c_{2}\gamma _{{\textbf{h}}}(1-\mu )\alpha _{1}}{\vartheta _{1}+\phi _{2}+\delta }\bigg ){\textbf{P}}_{{\textbf{h}}}-\frac{\Lambda _{1}}{{\textbf{X}}_{{\textbf{h}}}}-\frac{\mu \alpha _{1}}{{\textbf{Q}}_{{\textbf{h}}{\textbf{a}}}}-\frac{(1-\mu )\alpha _{1}}{{\textbf{Q}}_{{\textbf{h}}{\textbf{s}}}}\nonumber \\ {}{} & {} \quad -\frac{{\mathcal {X}}_{2}}{2}\big ({\textbf{X}}_{{\textbf{h}}}^{\lambda +1}+{\textbf{P}}_{{\textbf{h}}}^{\lambda +1}+{\textbf{Q}}_{{\textbf{h}}{\textbf{a}}}^{\lambda +1}+{\textbf{Q}}_{{\textbf{h}}{\textbf{s}}}^{\lambda +1}+{\textbf{R}}_{{\textbf{h}}}^{\lambda +1}+{\textbf{X}}_{{\textbf{r}}}^{\lambda +1}+{\textbf{P}}_{{\textbf{r}}}^{\lambda +1}+{\textbf{Q}}_{{\textbf{r}}}^{\lambda +1}+{\textbf{G}}_{{\textbf{s}}}^{\lambda +1}+{\textbf{G}}_{{\textbf{a}}}^{\lambda +1}\big )\nonumber \\ {}{} & {} \quad +{\mathcal {X}}_{1}+\Lambda _{1}+5\vartheta _{1}+3\vartheta _{2}+\Upsilon _{{\textbf{h}}}{\textbf{X}}_{{\textbf{h}}}+\Upsilon _{{\textbf{h}}}{\textbf{P}}_{{\textbf{h}}}+\phi _{1}+\phi _{2}+\delta +\upsilon +\alpha _{2}+\xi _{2}+\xi _{3}+\frac{1}{2}(\sigma _{1}^{2}\vee \sigma _{3}^{2}\vee \sigma _{4}^{2}\nonumber \\ {}{} & {} \quad \vee \sigma _{5}^{2}\vee \sigma _{6}^{2}\vee \sigma _{7}^{2}\vee \sigma _{8}^{2}\vee \sigma _{9}^{2}\vee \sigma _{10}^{2}). \end{aligned}$$

We can now develop a restricted closed domain ${\mathcal {V}}_{\epsilon }$ as shown below

$$\begin{aligned} {\mathcal {V}}_{\epsilon }{} & {} =\Big \{({\tilde{\Theta }})^{{\tilde{T}}}\in \Re _{+}^{10}:{\textbf{X}}_{{\textbf{h}}}\in \big [\epsilon ,1/\epsilon \big ],{\textbf{P}}_{{\textbf{h}}}\in \big [\epsilon ,1/\epsilon \big ],{\textbf{Q}}_{{\textbf{h}}{\textbf{a}}}\in \big [\epsilon ^{2},1/\epsilon ^{2}\big ],{\textbf{Q}}_{{\textbf{h}}{\textbf{s}}}\in \big [\epsilon ^{2},1/\epsilon ^{2}\big ],{\textbf{R}}_{{\textbf{h}}}\in \big [\epsilon ^{3},1/\epsilon ^{3}\big ],\nonumber \\ {}{} & {} \quad {\textbf{X}}_{{\textbf{r}}}\in \big [\epsilon ^{3},1/\epsilon ^{3}\big ],{\textbf{P}}_{{\textbf{r}}}\in \big [\epsilon ^{4},1/\epsilon ^{4}\big ],{\textbf{Q}}_{{\textbf{r}}}\in \big [\epsilon ^{4},1/\epsilon ^{4}\big ],{\textbf{G}}_{{\textbf{s}}}\in \big [\epsilon ^{5},1/\epsilon ^{5}\big ],{\textbf{G}}_{{\textbf{a}}}\in \big [\epsilon ^{5},1/\epsilon ^{5}\big ]\Big \}, \end{aligned}$$

where $\epsilon \in (0,1)$ denotes a sufficiently small a fixed value. We can select a small sufficient size in the group $\Re _{+}^{10}\setminus {\mathcal {V}}_{\epsilon }$ to satisfy the subsequent requirements

$$\begin{aligned}{} & {} {\mathcal {X}}_{4}-\frac{\Lambda _{1}}{\epsilon }\le -1,~~~~\epsilon \le \frac{1}{{\mathbb {Q}}\big (\frac{c_{1}\gamma _{{\textbf{h}}}\mu \alpha _{1}}{\vartheta _{1}+\phi _{1}}+\frac{c_{2}\gamma _{{\textbf{h}}}(1-\mu )\alpha _{1}}{\vartheta _{1}+\phi _{2}}+\delta \big )}\nonumber \\{} & {} {\mathcal {X}}_{4}-\frac{\alpha _{1}\mu }{\epsilon ^{2}}\le -1,~~~~{\mathcal {X}}_{4}-\frac{\alpha _{1}(1-\mu )}{\epsilon ^{2}}\le -1,~~~~-\frac{{\mathcal {X}}_{2}}{4\epsilon ^{\lambda +1}}+{\mathcal {X}}_{4}\le -1,\nonumber \\{} & {} -\frac{{\mathcal {X}}_{2}}{4\epsilon ^{3(\lambda +1)}}+{\mathcal {X}}_{4}\le -1,~~~~-\frac{{\mathcal {X}}_{2}}{4\epsilon ^{4(\lambda +1)}}+{\mathcal {X}}_{4}\le -1,~~~~-\frac{{\mathcal {X}}_{2}}{5\epsilon ^{5(\lambda +1)}}+{\mathcal {X}}_{4}\le -1, \end{aligned}$$

where

70 $$\begin{aligned} {\mathcal {X}}_{4}{} & {} :=\sup \limits _{({\tilde{\Theta }})^{{\tilde{T}}}\in \Re _{+}^{10}}\bigg \{-\frac{{\mathcal {X}}_{2}}{4}(\sum \limits _{\iota =1}^{10}{\tilde{\Theta }}_{\iota }^{\lambda +1})+{\mathbb {Q}}\bigg (\frac{c_{1}\gamma _{{\textbf{h}}}\mu \alpha _{1}}{\vartheta _{1}+\phi _{1}}+\frac{c_{2}\gamma _{{\textbf{h}}}(1-\mu )\alpha _{1}}{\vartheta _{1}+\phi _{2}+\delta }\bigg ){\textbf{P}}_{{\textbf{h}}}+{\mathcal {X}}_{1}+\Lambda _{1}+5\vartheta _{1}+3\vartheta _{2}\nonumber \\ {}{} & {} \quad +\Upsilon _{{\textbf{h}}}{\textbf{X}}_{{\textbf{h}}}+\Upsilon _{{\textbf{h}}}{\textbf{P}}_{{\textbf{h}}}+\phi _{1}+\phi _{2}+\delta +\upsilon +\alpha _{2}+\xi _{2}+\xi _{3}+\frac{1}{2}(\sigma _{1}^{2}\vee \sigma _{3}^{2}\vee \sigma _{4}^{2}\vee \sigma _{5}^{2}\vee \sigma _{6}^{2}\vee \sigma _{7}^{2}\nonumber \\ {}{} & {} \quad \vee \sigma _{8}^{2}\vee \sigma _{9}^{2}\vee \sigma _{10}^{2})\bigg \}.\end{aligned}$$

For simplicity, we are able to split $\Re _{+}^{10}\setminus {\mathcal {V}}_{\epsilon }$ into sixteen separate regions as

71 $$\begin{aligned}{} & {} {\mathcal {V}}_{\epsilon }^{1}=\big \{{\tilde{\Theta }}^{{\tilde{T}}}\in \Re 
_{+}^{10}:{\textbf{X}}_{{\textbf{h}}}<\epsilon \big \},~~{\mathcal {V}}_{\epsilon }^{2}=\big \{{\tilde{\Theta }}^{{\tilde{T}}}\in \Re _{+}^{10}:{\textbf{P}}_{{\textbf{h}}}<\epsilon \big \},~~{\mathcal {V}}_{\epsilon }^{3}=\big \{{\tilde{\Theta }}^{{\tilde{T}}}\in \Re _{+}^{10}:{\textbf{Q}}_{{\textbf{h}}{\textbf{a}}}<\epsilon ^{2},{\textbf{P}}_{{\textbf{h}}}\ge \epsilon \big \},\nonumber \\ {}{} & {} {\mathcal {V}}_{\epsilon }^{4}=\big \{{\tilde{\Theta }}^{{\tilde{T}}}\in \Re _{+}^{10}:{\textbf{Q}}_{{\textbf{h}}{\textbf{s}}}<\epsilon ^{2},{\textbf{P}}_{{\textbf{h}}}\ge \epsilon \big \},~~{\mathcal {V}}_{\epsilon }^{5}=\big \{{\tilde{\Theta }}^{{\tilde{T}}}\in \Re _{+}^{10}:{\textbf{X}}_{{\textbf{h}}}>1/\epsilon \big \},~~{\mathcal {V}}_{\epsilon }^{6}=\big \{{\tilde{\Theta }}^{{\tilde{T}}}\in \Re _{+}^{10}:{\textbf{P}}_{{\textbf{h}}}>1/\epsilon \big \},\nonumber \\ {}{} & {} {\mathcal {V}}_{\epsilon }^{7}=\big \{{\tilde{\Theta }}^{{\tilde{T}}}\in \Re _{+}^{10}:{\textbf{Q}}_{{\textbf{h}}{\textbf{a}}}>1/\epsilon ^{2}\big \},~~{\mathcal {V}}_{\epsilon }^{8}=\big \{{\tilde{\Theta }}^{{\tilde{T}}}\in \Re _{+}^{10}:{\textbf{Q}}_{{\textbf{h}}{\textbf{s}}}>1/\epsilon ^{2}\big \},~~{\mathcal {V}}_{\epsilon }^{9}=\big \{{\tilde{\Theta }}^{{\tilde{T}}}\in \Re _{+}^{10}:{\textbf{X}}_{{\textbf{r}}}>1/\epsilon ^{3}\big \},\nonumber \\ {}{} & {} {\mathcal {V}}_{\epsilon }^{10}=\big \{{\tilde{\Theta }}^{{\tilde{T}}}\in \Re _{+}^{10}:{\textbf{P}}_{{\textbf{r}}}>1/\epsilon ^{4}\big \},~~{\mathcal {V}}_{\epsilon }^{11}=\big \{{\tilde{\Theta }}^{{\tilde{T}}}\in \Re _{+}^{10}:{\textbf{Q}}_{{\textbf{r}}}>1/\epsilon ^{4}\big \},~~{\mathcal {V}}_{\epsilon }^{12}=\big \{{\tilde{\Theta }}^{{\tilde{T}}}\in \Re _{+}^{10}:{\textbf{G}}_{{\textbf{s}}}>1/\epsilon ^{5}\big \},\nonumber \\ {}{} & {} {\mathcal {V}}_{\epsilon }^{13}=\big \{{\tilde{\Theta }}^{{\tilde{T}}}\in \Re _{+}^{10}:{\textbf{G}}_{{\textbf{a}}}>1/\epsilon ^{5}\big \},~~{\mathcal {V}}_{\epsilon }^{14}=\big \{{\tilde{\Theta }}^{{\tilde{T}}}\in \Re _{+}^{10}:{\textbf{X}}_{{\textbf{r}}}<\epsilon ^{3},{\textbf{P}}_{{\textbf{r}}}\ge \epsilon ^{4}\big \},\nonumber \\ {}{} & {} {\mathcal {V}}_{\epsilon }^{15}=\big \{{\tilde{\Theta }}^{{\tilde{T}}}\in \Re _{+}^{10}:{\textbf{X}}_{{\textbf{r}}}<\epsilon ^{3},{\textbf{Q}}_{{\textbf{r}}}\ge \epsilon ^{4}\big \},~~~~ {\mathcal {V}}_{\epsilon }^{16}=\big \{{\tilde{\Theta }}^{{\tilde{T}}}\in \Re _{+}^{10}:{\textbf{Q}}_{{\textbf{r}}}<\epsilon ^{4},{\textbf{G}}_{{\textbf{s}}}\ge \epsilon ^{5}\big \}. \end{aligned}$$

Evidently, $\Re _{+}^{10}\setminus {\mathcal {V}}_{\epsilon }={\mathcal {V}}_{1}\cup ...\cup {\mathcal {V}}_{10}.$ Thus, it is not challenging to demonstrate this ${\mathbb {L}}{\tilde{W}}\le -1~\forall ~{\tilde{\Theta }}^{{\tilde{T}}}\in {\mathcal {V}}_{\epsilon }^{c}.$ For reference, (see;^42,43^).

As a result, the requirement $(Z_{2})$ in Lemma 3.5 as well possesses. According to Lemma 3.5, mechanism (5) has a unique stationary distribution $\pi (.)$ and the ergodicity applies. This completes the proof.

$$\square$$

## Numerical simulations

This section devotes itself to implementing the piecewise derivatives whenever the interrelated derivatives are the deterministic and fractional differential operators, taking into account local/nonlocal and singular/non-singular kernels. Thus, the order of the derivative $\chi$ lies in (0,1].

### Fractional derivative with power kernel

In this segment, we shall examine the dynamical analysis of Lassa fever models (3) and (5)to ascertain how distinctive pathogen advancement mechanisms, which include those that are typically disregarded, like particulate and environmental interfacial pathways, affect both individuals and rodents using classical, index-law, and subsequently stochastic methods. The computational mechanism will be established in the initial process utilizing the classical derivative enactment, followed by the power law kernel in the second level, and eventually the stochastic surroundings in the final stages, if we describe ${\mathbb {T}}$ as the ultimate propagation duration, such that the final attempt. The explanation for this hypothesis is then given using the corresponding formulaic framework:

72 $$\begin{aligned} {\left\{ \begin{array}{ll} \dot{{\textbf{X}}_{{\textbf{h}}}}=\Lambda _{1}-\Upsilon _{{\textbf{h}}}{\textbf{X}}_{{\textbf{h}}}-\vartheta _{1}{\textbf{X}}_{{\textbf{h}}},\\ \dot{{\textbf{P}}_{{\textbf{h}}}}=\Upsilon _{{\textbf{h}}}{\textbf{X}}_{{\textbf{h}}}-(\alpha _{1}+\vartheta _{1}){\textbf{P}}_{{\textbf{h}}},\\ \dot{{\textbf{Q}}_{{\textbf{h}}{\textbf{a}}}}=\mu \alpha _{1}{\textbf{P}}_{{\textbf{h}}}-(\phi _{1}+\vartheta _{1}){\textbf{Q}}_{{\textbf{h}}{\textbf{a}}},\\ \dot{{\textbf{Q}}_{{\textbf{h}}{\textbf{s}}}}=(1-\mu )\alpha _{1}{\textbf{P}}_{{\textbf{h}}}-(\delta +\phi _{2}+\vartheta _{1}){\textbf{Q}}_{{\textbf{h}}{\textbf{s}}},\\ \dot{{\textbf{R}}_{{\textbf{h}}}}=\phi _{1}{\textbf{P}}_{{\textbf{h}}}+\phi _{2}{\textbf{Q}}_{{\textbf{h}}{\textbf{s}}}-\vartheta _{1}{\textbf{R}}_{{\textbf{h}}},\\ \dot{{\textbf{X}}_{{\textbf{r}}}}=\Lambda _{2}-\Upsilon _{{\textbf{r}}}{\textbf{X}}_{{\textbf{r}}}-(\vartheta _{2}+\upsilon ){\textbf{R}}_{{\textbf{h}}},~~~~if~~{\textbf{t}}\in [0,{\mathbb {T}}_{1}],\\ \dot{{\textbf{P}}_{{\textbf{r}}}}=\Upsilon _{{\textbf{r}}}{\textbf{X}}_{{\textbf{r}}}-(\alpha _{2}+\vartheta _{2}+\upsilon ){\textbf{P}}_{{\textbf{h}}},\\ \dot{{\textbf{Q}}_{{\textbf{r}}}}=\alpha _{2}{\textbf{P}}_{{\textbf{r}}}-(\upsilon +\vartheta _{2}){\textbf{Q}}_{{\textbf{r}}},\\ \dot{{\textbf{G}}_{{\textbf{s}}}}=\beta _{1}{\textbf{Q}}_{{\textbf{h}}{\textbf{a}}}+\beta _{2}{\textbf{Q}}_{{\textbf{h}}{\textbf{s}}}+\beta _{3}{\textbf{Q}}_{{\textbf{r}}}-(\xi _{2}+\xi _{3}){\textbf{G}}_{{\textbf{s}}},\\ \dot{{\textbf{G}}_{{\textbf{a}}}}=\xi _{3}{\textbf{G}}_{{\textbf{s}}}-\xi _{2}{\textbf{G}}_{{\textbf{a}}}, \\ \end{array}\right. } \end{aligned}$$

73 $$\begin{aligned} {\left\{ \begin{array}{ll} \,_{0}^{c}{\textbf{D}}_{{\textbf{t}}}^{\chi }{{\textbf{X}}_{{\textbf{h}}}}=\Lambda _{1}-\Upsilon _{{\textbf{h}}}{\textbf{X}}_{{\textbf{h}}}-\vartheta _{1}{\textbf{X}}_{{\textbf{h}}},\\ \,_{0}^{c}{\textbf{D}}_{{\textbf{t}}}^{\chi }{{\textbf{P}}_{{\textbf{h}}}}=\Upsilon _{{\textbf{h}}}{\textbf{X}}_{{\textbf{h}}}-(\alpha _{1}+\vartheta _{1}){\textbf{P}}_{{\textbf{h}}},\\ \,_{0}^{c}{\textbf{D}}_{{\textbf{t}}}^{\chi }{{\textbf{Q}}_{{\textbf{h}}{\textbf{a}}}}=\mu \alpha _{1}{\textbf{P}}_{{\textbf{h}}}-(\phi _{1}+\vartheta _{1}){\textbf{Q}}_{{\textbf{h}}{\textbf{a}}},\\ \,_{0}^{c}{\textbf{D}}_{{\textbf{t}}}^{\chi }{{\textbf{Q}}_{{\textbf{h}}{\textbf{s}}}}=(1-\mu )\alpha _{1}{\textbf{P}}_{{\textbf{h}}}-(\delta +\phi _{2}+\vartheta _{1}){\textbf{Q}}_{{\textbf{h}}{\textbf{s}}},\\ \,_{0}^{c}{\textbf{D}}_{{\textbf{t}}}^{\chi }{{\textbf{R}}_{{\textbf{h}}}}=\phi _{1}{\textbf{P}}_{{\textbf{h}}}+\phi _{2}{\textbf{Q}}_{{\textbf{h}}{\textbf{s}}}-\vartheta _{1}{\textbf{R}}_{{\textbf{h}}},\\ \,_{0}^{c}{\textbf{D}}_{{\textbf{t}}}^{\chi }{{\textbf{X}}_{{\textbf{r}}}}=\Lambda _{2}-\Upsilon _{{\textbf{r}}}{\textbf{X}}_{{\textbf{r}}}-(\vartheta _{2}+\upsilon ){\textbf{R}}_{{\textbf{h}}},~~~~if~~{\textbf{t}}\in [{\mathbb {T}}_{1},{\mathbb {T}}_{2}],\\ \,_{0}^{c}{\textbf{D}}_{{\textbf{t}}}^{\chi }{{\textbf{P}}_{{\textbf{r}}}}=\Upsilon _{{\textbf{r}}}{\textbf{X}}_{{\textbf{r}}}-(\alpha _{2}+\vartheta _{2}+\upsilon ){\textbf{P}}_{{\textbf{h}}},\\ \,_{0}^{c}{\textbf{D}}_{{\textbf{t}}}^{\chi }{{\textbf{Q}}_{{\textbf{r}}}}=\alpha _{2}{\textbf{P}}_{{\textbf{r}}}-(\upsilon +\vartheta _{2}){\textbf{Q}}_{{\textbf{r}}},\\ \,_{0}^{c}{\textbf{D}}_{{\textbf{t}}}^{\chi }{{\textbf{G}}_{{\textbf{s}}}}=\beta _{1}{\textbf{Q}}_{{\textbf{h}}{\textbf{a}}}+\beta _{2}{\textbf{Q}}_{{\textbf{h}}{\textbf{s}}}+\beta _{3}{\textbf{Q}}_{{\textbf{r}}}-(\xi _{2}+\xi _{3}){\textbf{G}}_{{\textbf{s}}},\\ \,_{0}^{c}{\textbf{D}}_{{\textbf{t}}}^{\chi }{{\textbf{G}}_{{\textbf{a}}}}=\xi _{3}{\textbf{G}}_{{\textbf{s}}}-\xi _{2}{\textbf{G}}_{{\textbf{a}}}, \\ \end{array}\right. } \end{aligned}$$

74 $$\begin{aligned} {\left\{ \begin{array}{ll} d{\textbf{X}}_{{\textbf{h}}}=\big [\Lambda _{1}-\Upsilon _{{\textbf{h}}}{\textbf{X}}_{{\textbf{h}}}-\vartheta _{1}{\textbf{X}}_{{\textbf{h}}}\big ]d{\textbf{t}}+\sigma _{1}{\textbf{X}}_{{\textbf{h}}}d{\mathcal {B}}_{1}({\textbf{t}}),\\ d{\textbf{P}}_{{\textbf{h}}}=\big [\Upsilon _{{\textbf{h}}}{\textbf{X}}_{{\textbf{h}}}-(\alpha _{1}+\vartheta _{1}){\textbf{P}}_{{\textbf{h}}}\big ]d{\textbf{t}}+\sigma _{2}{\textbf{P}}_{{\textbf{h}}}d{\mathcal {B}}_{2}({\textbf{t}}),\\ d{\textbf{Q}}_{{\textbf{h}}{\textbf{a}}}=\big [\mu \alpha _{1}{\textbf{P}}_{{\textbf{h}}}-(\phi _{1}+\vartheta _{1}){\textbf{Q}}_{{\textbf{h}}{\textbf{a}}}\big ]d{\textbf{t}}+\sigma _{3}{\textbf{Q}}_{{\textbf{h}}{\textbf{a}}}d{\mathcal {B}}_{3}({\textbf{t}}),\\ d{\textbf{Q}}_{{\textbf{h}}{\textbf{s}}}=\big [(1-\mu )\alpha _{1}{\textbf{P}}_{{\textbf{h}}}-(\delta +\phi _{2}+\vartheta _{1}){\textbf{Q}}_{{\textbf{h}}{\textbf{s}}}\big ]d{\textbf{t}}+\sigma _{4}{\textbf{Q}}_{{\textbf{h}}{\textbf{s}}}d{\mathcal {B}}_{4}({\textbf{t}}),\\ d{\textbf{R}}_{{\textbf{h}}}=\big [\phi _{1}{\textbf{P}}_{{\textbf{h}}}+\phi _{2}{\textbf{Q}}_{{\textbf{h}}{\textbf{s}}}-\vartheta _{1}{\textbf{R}}_{{\textbf{h}}}\big ]d{\textbf{t}}+\sigma _{5}{\textbf{R}}_{{\textbf{h}}}d{\mathcal {B}}_{5}({\textbf{t}}),~~~~~~{\textbf{t}}\in [{\mathbb {T}}_{2},{\mathbb {T}}],\\ d{\textbf{X}}_{{\textbf{r}}}=\big [\Lambda _{2}-\Upsilon _{{\textbf{r}}}{\textbf{X}}_{{\textbf{r}}}-(\vartheta _{2}+\upsilon ){\textbf{R}}_{{\textbf{h}}}\big ]d{\textbf{t}}+\sigma _{6}{\textbf{X}}_{{\textbf{r}}}d{\mathcal {B}}_{6}({\textbf{t}}),\\ d{\textbf{P}}_{{\textbf{r}}}=\big [\Upsilon _{{\textbf{r}}}{\textbf{X}}_{{\textbf{r}}}-(\alpha _{2}+\vartheta _{2}+\upsilon ){\textbf{P}}_{{\textbf{h}}}\big ]d{\textbf{t}}+\sigma _{7}{\textbf{P}}_{{\textbf{r}}}d{\mathcal {B}}_{7}({\textbf{t}}),\\ d{\textbf{Q}}_{{\textbf{r}}}=\big [\alpha _{2}{\textbf{P}}_{{\textbf{r}}}-(\upsilon +\vartheta _{2}){\textbf{Q}}_{{\textbf{r}}}\big ]d{\textbf{t}}+\sigma _{8}{\textbf{Q}}_{{\textbf{r}}}d{\mathcal {B}}_{8}({\textbf{t}}),\\ d{\textbf{G}}_{{\textbf{s}}}=\big [\beta _{1}{\textbf{Q}}_{{\textbf{h}}{\textbf{a}}}+\beta _{2}{\textbf{Q}}_{{\textbf{h}}{\textbf{s}}}+\beta _{3}{\textbf{Q}}_{{\textbf{r}}}-(\xi _{2}+\xi _{3}){\textbf{G}}_{{\textbf{s}}}\big ]d{\textbf{t}}+\sigma _{9}{\textbf{G}}_{{\textbf{s}}}d{\mathcal {B}}_{9}({\textbf{t}}),\\ d{\textbf{G}}_{{\textbf{a}}}=\big [\xi _{3}{\textbf{G}}_{{\textbf{s}}}-\xi _{2}{\textbf{G}}_{{\textbf{a}}}\big ]d{\textbf{t}}+\sigma _{10}{\textbf{G}}_{{\textbf{a}}}d{\mathcal {B}}_{10}({\textbf{t}}),\end{array}\right. } \end{aligned}$$

Now, we use the method outlined in^37^ to analyze the piecewise configuration (72–74) in the context of Caputo’s derivative. We immediately begin the methodology by doing the following:

$$\begin{aligned} {\left\{ \begin{array}{ll} \frac{d\aleph _{{\textbf{k}}}({\textbf{t}})}{d{\textbf{t}}}=\Omega ({\textbf{t}},\aleph _{{\textbf{k}}}).~\aleph _{{\textbf{k}}}(0)=\aleph _{{\textbf{k}},0},~{\textbf{k}}=1,2,...,{\mathfrak {n}}~if~{\textbf{t}}\in [0,{\mathbb {T}}_{1}],\\ \,_{{\mathbb {T}}_{1}}^{c}{\textbf{D}}_{{\textbf{t}}}^{\chi }\aleph _{{\textbf{k}}}({\textbf{t}})=\Omega ({\textbf{t}},\aleph _{{\textbf{k}}}),~\aleph _{{\textbf{k}}}({\mathbb {T}}_{1})=\aleph _{{\textbf{k}},1},~if~{\textbf{t}}\in [{\mathbb {T}}_{1},{\mathbb {T}}_{2}],\\ d\aleph _{{\textbf{k}}}({\textbf{t}})=\Omega ({\textbf{t}},\aleph _{{\textbf{k}}})d{\textbf{t}}+\wp _{{\textbf{k}}}\aleph _{{\textbf{k}}}d{\mathcal {B}}_{{\textbf{k}}}({\textbf{t}}),~\aleph _{{\textbf{k}}}({\mathbb {T}}_{2})=\aleph _{{\textbf{k}},2},~if~{\textbf{t}}\in [{\mathbb {T}}_{2},{\mathbb {T}}]. \end{array}\right. } \end{aligned}$$

Consequently, we have

$$\begin{aligned} \aleph _{{\textbf{k}}}^{{\textbf{v}}}={\left\{ \begin{array}{ll}\aleph _{{\textbf{k}}}(0)+\sum \limits _{{\textbf{m}}=2}^{{\textbf{v}}}\Big \{\frac{23}{12}\Omega ({{\textbf{t}}}_{{\textbf{m}}},\aleph ^{{\textbf{m}}})\Delta {\textbf{t}}-\frac{4}{3}\Omega ({{\textbf{t}}}_{{\textbf{m}}-1},\aleph ^{{\textbf{m}}-1})\Delta {\textbf{t}}+\frac{5}{12}\Omega ({{\textbf{t}}}_{{\textbf{m}}-2},\aleph ^{{\textbf{m}}-2})\Delta {\textbf{t}}\Big \},~~{\textbf{t}}\in [0,\mathbb {T_{1}}].\\ \aleph _{{\textbf{k}}}({\mathbb {T}}_{1})+\frac{(\Delta {\textbf{t}})^{\chi -1}}{\Gamma (\chi +1)}\sum \limits _{{\textbf{m}}=2}^{{\textbf{v}}}\Omega ({{\textbf{t}}}_{{\textbf{m}}-2},\aleph ^{{\textbf{m}}-2})\Psi _{1}\\ \quad + \frac{(\Delta {\textbf{t}})^{\chi -1}}{\Gamma (\chi +2)}\sum \limits _{{\textbf{m}}=2}^{{\textbf{v}}}\Big \{\Omega ({{\textbf{t}}}_{{\textbf{m}}-1},\aleph ^{{\textbf{m}}-1})-\Omega ({{\textbf{t}}}_{{\textbf{m}}-2},\aleph ^{{\textbf{m}}-2})\Big \}\Psi _{2}\\ \quad +\frac{\chi (\Delta {\textbf{t}})^{\chi -1}}{2\Gamma (\chi +3)}\sum \limits _{{\textbf{m}}=2}^{{\textbf{v}}}\Big \{\Omega ({{\textbf{t}}}_{{\textbf{m}}},\aleph ^{{\textbf{m}}})-2\Omega ({{\textbf{t}}}_{{\textbf{m}}-1},\aleph ^{{\textbf{m}}-1})+\Omega ({{\textbf{t}}}_{{\textbf{m}}-2},\aleph ^{{\textbf{m}}-2})\Big \}\Psi _{3},~~{\textbf{t}}\in [{\mathbb {T}}_{1},{\mathbb {T}}_{2}],\\ \aleph _{{\textbf{k}}}({\mathbb {T}}_{2})+\sum \limits _{{\textbf{m}}={\textbf{v}}+3}^{{\mathfrak {n}}}\Big \{\frac{5}{12}\Omega ({{\textbf{t}}}_{{\textbf{m}}-2},\aleph ^{{\textbf{m}}-2})\Delta {\textbf{t}}-\frac{4}{3}\Omega ({{\textbf{t}}}_{{\textbf{m}}-1},\aleph ^{{\textbf{m}}-1})\Delta {\textbf{t}}+\frac{23}{12}\Omega ({{\textbf{t}}}_{{\textbf{m}}},\aleph ^{{\textbf{m}}})\Delta {\textbf{t}}\Big \} \\ \quad +\sum \limits _{{\textbf{m}}={\textbf{v}}+3}^{{\mathfrak {n}}}\Big \{\frac{5}{12}\big ({\mathcal {B}}({{\textbf{t}}}_{{\textbf{m}}-1})-{\mathcal {B}}({{\textbf{t}}}_{{\textbf{m}}-2})\big )\wp \aleph ^{{\textbf{m}}-2}- \frac{4}{3}\big ({\mathcal {B}}({{\textbf{t}}}_{{\textbf{m}}})-{\mathcal {B}}({{\textbf{t}}}_{{\textbf{m}}-1})\big )\wp \aleph ^{{\textbf{m}}-1}\\ \quad +\frac{23}{12}\big ({\mathcal {B}}({{\textbf{t}}}_{{\textbf{m}}+1})-{\mathcal {B}}({{\textbf{t}}}_{{\textbf{m}}})\big )\wp \aleph ^{{\textbf{m}}}\Big \},~~{\textbf{t}}\in [{\mathbb {T}}_{2},{\mathbb {T}}],\end{array}\right. } \end{aligned}$$

where

75 $$\begin{aligned} \Psi _{1}:=({\textbf{v}}-{\textbf{m}}-1)^{\chi }-({\textbf{v}}-{\textbf{m}})^{\chi }, \end{aligned}$$

76 $$\begin{aligned} \Psi _{2}:=({\textbf{v}}-{\textbf{m}}+1)^{\chi }({\textbf{v}}-{\textbf{m}}+2\chi +3)-({\textbf{v}}-{\textbf{m}})^{\chi }({\textbf{v}}-{\textbf{m}}+3\chi +3) \end{aligned}$$

and

77 $$\begin{aligned} \Psi _{3}:={\left\{ \begin{array}{ll}({\textbf{v}}-{\textbf{m}}+1)^{\chi }\Big (2({\textbf{v}}-{\textbf{m}})^{2}+ (3\chi +10)({\textbf{v}}-{\textbf{m}})+2\chi ^{2}+9\chi +12\Big )\\ \quad + ({\textbf{v}}-{\textbf{m}})^{\chi }\Big (2({\textbf{v}}-{\textbf{m}})^{2}+(5\chi +10)({\textbf{v}}-{\textbf{m}})+6\chi ^{2}+18\chi +12\Big ). \end{array}\right. } \end{aligned}$$

### Fractional derivative with exponential decay kernel

Now, we shall demonstrate the dynamical analysis of Lassa fever models (3) and (5)to ascertain how distinctive pathogen advancement mechanisms, which include those that are typically disregarded, like particulate and environmental interfacial pathways, affect both individuals and rodents using classical, exponential decay law and subsequently stochastic methods. The computational mechanism will be established in the initial process utilizing the classical derivative enactment, followed by the exponential decay kernel in the other level, and thus the stochastic surroundings in the final stages, if we describe ${\mathbb {T}}$ as the ultimate propagation duration, such that the final attempt. The explanation for this hypothesis is then given using the corresponding formulaic framework:

78 $$\begin{aligned} {\left\{ \begin{array}{ll} \dot{{\textbf{X}}_{{\textbf{h}}}}=\Lambda _{1}-\Upsilon _{{\textbf{h}}}{\textbf{X}}_{{\textbf{h}}}-\vartheta _{1}{\textbf{X}}_{{\textbf{h}}},\\ \dot{{\textbf{P}}_{{\textbf{h}}}}=\Upsilon _{{\textbf{h}}}{\textbf{X}}_{{\textbf{h}}}-(\alpha _{1}+\vartheta _{1}){\textbf{P}}_{{\textbf{h}}},\\ \dot{{\textbf{Q}}_{{\textbf{h}}{\textbf{a}}}}=\mu \alpha _{1}{\textbf{P}}_{{\textbf{h}}}-(\phi _{1}+\vartheta _{1}){\textbf{Q}}_{{\textbf{h}}{\textbf{a}}},\\ \dot{{\textbf{Q}}_{{\textbf{h}}{\textbf{s}}}}=(1-\mu )\alpha _{1}{\textbf{P}}_{{\textbf{h}}}-(\delta +\phi _{2}+\vartheta _{1}){\textbf{Q}}_{{\textbf{h}}{\textbf{s}}},\\ \dot{{\textbf{R}}_{{\textbf{h}}}}=\phi _{1}{\textbf{P}}_{{\textbf{h}}}+\phi _{2}{\textbf{Q}}_{{\textbf{h}}{\textbf{s}}}-\vartheta _{1}{\textbf{R}}_{{\textbf{h}}},\\ \dot{{\textbf{X}}_{{\textbf{r}}}}=\Lambda _{2}-\Upsilon _{{\textbf{r}}}{\textbf{X}}_{{\textbf{r}}}-(\vartheta _{2}+\upsilon ){\textbf{R}}_{{\textbf{h}}},~~~~if~~{\textbf{t}}\in [0,{\mathbb {T}}_{1}],\\ \dot{{\textbf{P}}_{{\textbf{r}}}}=\Upsilon _{{\textbf{r}}}{\textbf{X}}_{{\textbf{r}}}-(\alpha _{2}+\vartheta _{2}+\upsilon ){\textbf{P}}_{{\textbf{h}}},\\ \dot{{\textbf{Q}}_{{\textbf{r}}}}=\alpha _{2}{\textbf{P}}_{{\textbf{r}}}-(\upsilon +\vartheta _{2}){\textbf{Q}}_{{\textbf{r}}},\\ \dot{{\textbf{G}}_{{\textbf{s}}}}=\beta _{1}{\textbf{Q}}_{{\textbf{h}}{\textbf{a}}}+\beta _{2}{\textbf{Q}}_{{\textbf{h}}{\textbf{s}}}+\beta _{3}{\textbf{Q}}_{{\textbf{r}}}-(\xi _{2}+\xi _{3}){\textbf{G}}_{{\textbf{s}}},\\ \dot{{\textbf{G}}_{{\textbf{a}}}}=\xi _{3}{\textbf{G}}_{{\textbf{s}}}-\xi _{2}{\textbf{G}}_{{\textbf{a}}}, \\ \end{array}\right. } \end{aligned}$$

79 $$\begin{aligned} {\left\{ \begin{array}{ll} \,_{0}^{CF}{\textbf{D}}_{{\textbf{t}}}^{\chi }{{\textbf{X}}_{{\textbf{h}}}}=\Lambda _{1}-\Upsilon _{{\textbf{h}}}{\textbf{X}}_{{\textbf{h}}}-\vartheta _{1}{\textbf{X}}_{{\textbf{h}}},\\ \,_{0}^{CF}{\textbf{D}}_{{\textbf{t}}}^{\chi }{{\textbf{P}}_{{\textbf{h}}}}=\Upsilon _{{\textbf{h}}}{\textbf{X}}_{{\textbf{h}}}-(\alpha _{1}+\vartheta _{1}){\textbf{P}}_{{\textbf{h}}},\\ \,_{0}^{CF}{\textbf{D}}_{{\textbf{t}}}^{\chi }{{\textbf{Q}}_{{\textbf{h}}{\textbf{a}}}}=\mu \alpha _{1}{\textbf{P}}_{{\textbf{h}}}-(\phi _{1}+\vartheta _{1}){\textbf{Q}}_{{\textbf{h}}{\textbf{a}}},\\ \,_{0}^{CF}{\textbf{D}}_{{\textbf{t}}}^{\chi }{{\textbf{Q}}_{{\textbf{h}}{\textbf{s}}}}=(1-\mu )\alpha _{1}{\textbf{P}}_{{\textbf{h}}}-(\delta +\phi _{2}+\vartheta _{1}){\textbf{Q}}_{{\textbf{h}}{\textbf{s}}},\\ \,_{0}^{CF}{\textbf{D}}_{{\textbf{t}}}^{\chi }{{\textbf{R}}_{{\textbf{h}}}}=\phi _{1}{\textbf{P}}_{{\textbf{h}}}+\phi _{2}{\textbf{Q}}_{{\textbf{h}}{\textbf{s}}}-\vartheta _{1}{\textbf{R}}_{{\textbf{h}}},\\ \,_{0}^{CF}{\textbf{D}}_{{\textbf{t}}}^{\chi }{{\textbf{X}}_{{\textbf{r}}}}=\Lambda _{2}-\Upsilon _{{\textbf{r}}}{\textbf{X}}_{{\textbf{r}}}-(\vartheta _{2}+\upsilon ){\textbf{R}}_{{\textbf{h}}},~~~~if~~{\textbf{t}}\in [{\mathbb {T}}_{1},{\mathbb {T}}_{2}],\\ \,_{0}^{CF}{\textbf{D}}_{{\textbf{t}}}^{\chi }{{\textbf{P}}_{{\textbf{r}}}}=\Upsilon _{{\textbf{r}}}{\textbf{X}}_{{\textbf{r}}}-(\alpha _{2}+\vartheta _{2}+\upsilon ){\textbf{P}}_{{\textbf{h}}},\\ \,_{0}^{CF}{\textbf{D}}_{{\textbf{t}}}^{\chi }{{\textbf{Q}}_{{\textbf{r}}}}=\alpha _{2}{\textbf{P}}_{{\textbf{r}}}-(\upsilon +\vartheta _{2}){\textbf{Q}}_{{\textbf{r}}},\\ \,_{0}^{CF}{\textbf{D}}_{{\textbf{t}}}^{\chi }{{\textbf{G}}_{{\textbf{s}}}}=\beta _{1}{\textbf{Q}}_{{\textbf{h}}{\textbf{a}}}+\beta _{2}{\textbf{Q}}_{{\textbf{h}}{\textbf{s}}}+\beta _{3}{\textbf{Q}}_{{\textbf{r}}}-(\xi _{2}+\xi _{3}){\textbf{G}}_{{\textbf{s}}},\\ \,_{0}^{CF}{\textbf{D}}_{{\textbf{t}}}^{\chi }{{\textbf{G}}_{{\textbf{a}}}}=\xi _{3}{\textbf{G}}_{{\textbf{s}}}-\xi _{2}{\textbf{G}}_{{\textbf{a}}}, \\ \end{array}\right. } \end{aligned}$$

80 $$\begin{aligned} {\left\{ \begin{array}{ll} d{\textbf{X}}_{{\textbf{h}}}=\big [\Lambda _{1}-\Upsilon _{{\textbf{h}}}{\textbf{X}}_{{\textbf{h}}}-\vartheta _{1}{\textbf{X}}_{{\textbf{h}}}\big ]d{\textbf{t}}+\sigma _{1}{\textbf{X}}_{{\textbf{h}}}d{\mathcal {B}}_{1}({\textbf{t}}),\\ d{\textbf{P}}_{{\textbf{h}}}=\big [\Upsilon _{{\textbf{h}}}{\textbf{X}}_{{\textbf{h}}}-(\alpha _{1}+\vartheta _{1}){\textbf{P}}_{{\textbf{h}}}\big ]d{\textbf{t}}+\sigma _{2}{\textbf{P}}_{{\textbf{h}}}d{\mathcal {B}}_{2}({\textbf{t}}),\\ d{\textbf{Q}}_{{\textbf{h}}{\textbf{a}}}=\big [\mu \alpha _{1}{\textbf{P}}_{{\textbf{h}}}-(\phi _{1}+\vartheta _{1}){\textbf{Q}}_{{\textbf{h}}{\textbf{a}}}\big ]d{\textbf{t}}+\sigma _{3}{\textbf{Q}}_{{\textbf{h}}{\textbf{a}}}d{\mathcal {B}}_{3}({\textbf{t}}),\\ d{\textbf{Q}}_{{\textbf{h}}{\textbf{s}}}=\big [(1-\mu )\alpha _{1}{\textbf{P}}_{{\textbf{h}}}-(\delta +\phi _{2}+\vartheta _{1}){\textbf{Q}}_{{\textbf{h}}{\textbf{s}}}\big ]d{\textbf{t}}+\sigma _{4}{\textbf{Q}}_{{\textbf{h}}{\textbf{s}}}d{\mathcal {B}}_{4}({\textbf{t}}),\\ d{\textbf{R}}_{{\textbf{h}}}=\big [\phi _{1}{\textbf{P}}_{{\textbf{h}}}+\phi _{2}{\textbf{Q}}_{{\textbf{h}}{\textbf{s}}}-\vartheta _{1}{\textbf{R}}_{{\textbf{h}}}\big ]d{\textbf{t}}+\sigma _{5}{\textbf{R}}_{{\textbf{h}}}d{\mathcal {B}}_{5}({\textbf{t}}),~~~~~~{\textbf{t}}\in [{\mathbb {T}}_{2},{\mathbb {T}}],\\ d{\textbf{X}}_{{\textbf{r}}}=\big [\Lambda _{2}-\Upsilon _{{\textbf{r}}}{\textbf{X}}_{{\textbf{r}}}-(\vartheta _{2}+\upsilon ){\textbf{R}}_{{\textbf{h}}}\big ]d{\textbf{t}}+\sigma _{6}{\textbf{X}}_{{\textbf{r}}}d{\mathcal {B}}_{6}({\textbf{t}}),\\ d{\textbf{P}}_{{\textbf{r}}}=\big [\Upsilon _{{\textbf{r}}}{\textbf{X}}_{{\textbf{r}}}-(\alpha _{2}+\vartheta _{2}+\upsilon ){\textbf{P}}_{{\textbf{h}}}\big ]d{\textbf{t}}+\sigma _{7}{\textbf{P}}_{{\textbf{r}}}d{\mathcal {B}}_{7}({\textbf{t}}),\\ d{\textbf{Q}}_{{\textbf{r}}}=\big [\alpha _{2}{\textbf{P}}_{{\textbf{r}}}-(\upsilon +\vartheta _{2}){\textbf{Q}}_{{\textbf{r}}}\big ]d{\textbf{t}}+\sigma _{8}{\textbf{Q}}_{{\textbf{r}}}d{\mathcal {B}}_{8}({\textbf{t}}),\\ d{\textbf{G}}_{{\textbf{s}}}=\big [\beta _{1}{\textbf{Q}}_{{\textbf{h}}{\textbf{a}}}+\beta _{2}{\textbf{Q}}_{{\textbf{h}}{\textbf{s}}}+\beta _{3}{\textbf{Q}}_{{\textbf{r}}}-(\xi _{2}+\xi _{3}){\textbf{G}}_{{\textbf{s}}}\big ]d{\textbf{t}}+\sigma _{9}{\textbf{G}}_{{\textbf{s}}}d{\mathcal {B}}_{9}({\textbf{t}}),\\ d{\textbf{G}}_{{\textbf{a}}}=\big [\xi _{3}{\textbf{G}}_{{\textbf{s}}}-\xi _{2}{\textbf{G}}_{{\textbf{a}}}\big ]d{\textbf{t}}+\sigma _{10}{\textbf{G}}_{{\textbf{a}}}d{\mathcal {B}}_{10}({\textbf{t}}),\end{array}\right. } \end{aligned}$$

Now, we use the method outlined in^37^ to analyze the piecewise configuration (78–80) in the context of Caputo-Fabrizio derivative. We immediately begin the methodology by doing the following:

81 $$\begin{aligned} {\left\{ \begin{array}{ll} \frac{d\aleph _{{\textbf{k}}}({\textbf{t}})}{d{\textbf{t}}}=\Omega ({\textbf{t}},\aleph _{{\textbf{k}}}).~\aleph _{{\textbf{k}}}(0)=\aleph _{{\textbf{k}},0},~{\textbf{k}}=1,2,...,{\mathfrak {n}}~if~{\textbf{t}}\in [0,{\mathbb {T}}_{1}],\\ \,_{{\mathbb {T}}_{1}}^{CF}{\textbf{D}}_{{\textbf{t}}}^{\chi }\aleph _{{\textbf{k}}}({\textbf{t}})=\Omega ({\textbf{t}},\aleph _{{\textbf{k}}}),~\aleph _{{\textbf{k}}}({\mathbb {T}}_{1})=\aleph _{{\textbf{k}},1},~if~{\textbf{t}}\in [{\mathbb {T}}_{1},{\mathbb {T}}_{2}],\\ d\aleph _{{\textbf{k}}}({\textbf{t}})=\Omega ({\textbf{t}},\aleph _{{\textbf{k}}})d{\textbf{t}}+\wp _{{\textbf{k}}}\aleph _{{\textbf{k}}}d{\mathcal {B}}_{{\textbf{k}}}({\textbf{t}}),~\aleph _{{\textbf{k}}}({\mathbb {T}}_{2})=\aleph _{{\textbf{k}},2},~if~{\textbf{t}}\in [{\mathbb {T}}_{2},{\mathbb {T}}]. \end{array}\right. } \end{aligned}$$

It follows that

82 $$\begin{aligned} \aleph _{{\textbf{k}}}^{{\textbf{v}}}={\left\{ \begin{array}{ll}\aleph _{{\textbf{k}}}(0)+\sum \limits _{{\textbf{m}}=2}^{{\textbf{v}}}\Big \{\frac{23}{12}\Omega ({{\textbf{t}}}_{{\textbf{m}}},\aleph ^{{\textbf{m}}})\Delta {\textbf{t}}-\frac{4}{3}\Omega ({{\textbf{t}}}_{{\textbf{m}}-1},\aleph ^{{\textbf{m}}-1})\Delta {\textbf{t}}+\frac{5}{12}\Omega ({{\textbf{t}}}_{{\textbf{m}}-2},\aleph ^{{\textbf{m}}-2})\Delta {\textbf{t}}\Big \},~~{\textbf{t}}\in [0,\mathbb {T_{1}}].\\ \aleph _{{\textbf{k}}}({\mathbb {T}}_{1})+\frac{1-\chi }{{\mathbb {M}}(\chi )}\Omega ({{\textbf{t}}}_{{\mathfrak {n}}},\aleph ^{{\mathfrak {n}}})+\frac{\chi }{{\mathbb {M}}(\chi )}\sum \limits _{{\textbf{m}}=2}^{{\textbf{v}}}\Big \{\frac{5}{12}\Omega ({{\textbf{t}}}_{{\textbf{m}}-2},\aleph ^{{\textbf{m}}-2})\Delta {\textbf{t}}-\frac{4}{3}\Omega ({{\textbf{t}}}_{{\textbf{m}}-1},\aleph ^{{\textbf{m}}-1})\Delta {\textbf{t}}\\ \quad +\frac{23}{12}\Omega ({{\textbf{t}}}_{{\textbf{m}}},\aleph ^{{\textbf{m}}})\Delta {\textbf{t}}\Big \},~~{\textbf{t}}\in [{\mathbb {T}}_{1},{\mathbb {T}}_{2}],\\ \aleph _{{\textbf{k}}}({\mathbb {T}}_{2})+\sum \limits _{{\textbf{m}}={\textbf{v}}+3}^{{\mathfrak {n}}}\Big \{\frac{5}{12}\Omega ({{\textbf{t}}}_{{\textbf{m}}-2},\aleph ^{{\textbf{m}}-2})\Delta {\textbf{t}}-\frac{4}{3}\Omega ({{\textbf{t}}}_{{\textbf{m}}-1},\aleph ^{{\textbf{m}}-1})\Delta {\textbf{t}}+\frac{23}{12}\Omega ({{\textbf{t}}}_{{\textbf{m}}},\aleph ^{{\textbf{m}}})\Delta {\textbf{t}}\Big \}\\ \quad +\sum \limits _{{\textbf{m}}={\textbf{v}}+3}^{{\mathfrak {n}}}\Big \{\frac{5}{12}\big ({\mathcal {B}}({{\textbf{t}}}_{{\textbf{m}}-1})-{\mathcal {B}}({{\textbf{t}}}_{{\textbf{m}}-2})\big )\wp \aleph ^{{\textbf{m}}-2}- \frac{4}{3}\big ({\mathcal {B}}({{\textbf{t}}}_{{\textbf{m}}})-{\mathcal {B}}({{\textbf{t}}}_{{\textbf{m}}-1})\big )\wp \aleph ^{{\textbf{m}}-1}\\ \quad +\frac{23}{12}\big ({\mathcal {B}}({{\textbf{t}}}_{{\textbf{m}}+1})-{\mathcal {B}}({{\textbf{t}}}_{{\textbf{m}}})\big )\wp \aleph ^{{\textbf{m}}}\Big \},~~{\textbf{t}}\in [{\mathbb {T}}_{2},{\mathbb {T}}].\end{array}\right. } \end{aligned}$$

### Fractional derivative with generalized Mittag-Leffler kernel

In what follows, we shall illustrate the dynamical analysis of Lassa fever models (3) and (5) to ascertain how distinctive pathogen advancement mechanisms, which include those that are typically disregarded, like particulate and environmental interfacial pathways, affect both individuals and rodents using classical, Mittag-Leffler law and subsequently stochastic methods. The computational mechanism will be established in the initial process utilizing the classical derivative enactment, followed by the generalized Mittag-Leffler kernel in the second level, and thus the stochastic surroundings in the final stages, if we describe ${\mathbb {T}}$ as the ultimate propagation duration, such that the final attempt. The explanation for this hypothesis is then given using the corresponding formulaic framework:

83 $$\begin{aligned} {\left\{ \begin{array}{ll} \dot{{\textbf{X}}_{{\textbf{h}}}}=\Lambda _{1}-\Upsilon _{{\textbf{h}}}{\textbf{X}}_{{\textbf{h}}}-\vartheta _{1}{\textbf{X}}_{{\textbf{h}}},\\ \dot{{\textbf{P}}_{{\textbf{h}}}}=\Upsilon _{{\textbf{h}}}{\textbf{X}}_{{\textbf{h}}}-(\alpha _{1}+\vartheta _{1}){\textbf{P}}_{{\textbf{h}}},\\ \dot{{\textbf{Q}}_{{\textbf{h}}{\textbf{a}}}}=\mu \alpha _{1}{\textbf{P}}_{{\textbf{h}}}-(\phi _{1}+\vartheta _{1}){\textbf{Q}}_{{\textbf{h}}{\textbf{a}}},\\ \dot{{\textbf{Q}}_{{\textbf{h}}{\textbf{s}}}}=(1-\mu )\alpha _{1}{\textbf{P}}_{{\textbf{h}}}-(\delta +\phi _{2}+\vartheta _{1}){\textbf{Q}}_{{\textbf{h}}{\textbf{s}}},\\ \dot{{\textbf{R}}_{{\textbf{h}}}}=\phi _{1}{\textbf{P}}_{{\textbf{h}}}+\phi _{2}{\textbf{Q}}_{{\textbf{h}}{\textbf{s}}}-\vartheta _{1}{\textbf{R}}_{{\textbf{h}}},\\ \dot{{\textbf{X}}_{{\textbf{r}}}}=\Lambda _{2}-\Upsilon _{{\textbf{r}}}{\textbf{X}}_{{\textbf{r}}}-(\vartheta _{2}+\upsilon ){\textbf{R}}_{{\textbf{h}}},~~~~if~~{\textbf{t}}\in [0,{\mathbb {T}}_{1}],\\ \dot{{\textbf{P}}_{{\textbf{r}}}}=\Upsilon _{{\textbf{r}}}{\textbf{X}}_{{\textbf{r}}}-(\alpha _{2}+\vartheta _{2}+\upsilon ){\textbf{P}}_{{\textbf{h}}},\\ \dot{{\textbf{Q}}_{{\textbf{r}}}}=\alpha _{2}{\textbf{P}}_{{\textbf{r}}}-(\upsilon +\vartheta _{2}){\textbf{Q}}_{{\textbf{r}}},\\ \dot{{\textbf{G}}_{{\textbf{s}}}}=\beta _{1}{\textbf{Q}}_{{\textbf{h}}{\textbf{a}}}+\beta _{2}{\textbf{Q}}_{{\textbf{h}}{\textbf{s}}}+\beta _{3}{\textbf{Q}}_{{\textbf{r}}}-(\xi _{2}+\xi _{3}){\textbf{G}}_{{\textbf{s}}},\\ \dot{{\textbf{G}}_{{\textbf{a}}}}=\xi _{3}{\textbf{G}}_{{\textbf{s}}}-\xi _{2}{\textbf{G}}_{{\textbf{a}}}, \\ \end{array}\right. } \end{aligned}$$

84 $$\begin{aligned} {\left\{ \begin{array}{ll} \,_{0}^{CF}{\textbf{D}}_{{\textbf{t}}}^{\chi }{{\textbf{X}}_{{\textbf{h}}}}=\Lambda _{1}-\Upsilon _{{\textbf{h}}}{\textbf{X}}_{{\textbf{h}}}-\vartheta _{1}{\textbf{X}}_{{\textbf{h}}},\\ \,_{0}^{CF}{\textbf{D}}_{{\textbf{t}}}^{\chi }{{\textbf{P}}_{{\textbf{h}}}}=\Upsilon _{{\textbf{h}}}{\textbf{X}}_{{\textbf{h}}}-(\alpha _{1}+\vartheta _{1}){\textbf{P}}_{{\textbf{h}}},\\ \,_{0}^{CF}{\textbf{D}}_{{\textbf{t}}}^{\chi }{{\textbf{Q}}_{{\textbf{h}}{\textbf{a}}}}=\mu \alpha _{1}{\textbf{P}}_{{\textbf{h}}}-(\phi _{1}+\vartheta _{1}){\textbf{Q}}_{{\textbf{h}}{\textbf{a}}},\\ \,_{0}^{CF}{\textbf{D}}_{{\textbf{t}}}^{\chi }{{\textbf{Q}}_{{\textbf{h}}{\textbf{s}}}}=(1-\mu )\alpha _{1}{\textbf{P}}_{{\textbf{h}}}-(\delta +\phi _{2}+\vartheta _{1}){\textbf{Q}}_{{\textbf{h}}{\textbf{s}}},\\ \,_{0}^{CF}{\textbf{D}}_{{\textbf{t}}}^{\chi }{{\textbf{R}}_{{\textbf{h}}}}=\phi _{1}{\textbf{P}}_{{\textbf{h}}}+\phi _{2}{\textbf{Q}}_{{\textbf{h}}{\textbf{s}}}-\vartheta _{1}{\textbf{R}}_{{\textbf{h}}},\\ \,_{0}^{CF}{\textbf{D}}_{{\textbf{t}}}^{\chi }{{\textbf{X}}_{{\textbf{r}}}}=\Lambda _{2}-\Upsilon _{{\textbf{r}}}{\textbf{X}}_{{\textbf{r}}}-(\vartheta _{2}+\upsilon ){\textbf{R}}_{{\textbf{h}}},~~~~if~~{\textbf{t}}\in [{\mathbb {T}}_{1},{\mathbb {T}}_{2}],\\ \,_{0}^{CF}{\textbf{D}}_{{\textbf{t}}}^{\chi }{{\textbf{P}}_{{\textbf{r}}}}=\Upsilon _{{\textbf{r}}}{\textbf{X}}_{{\textbf{r}}}-(\alpha _{2}+\vartheta _{2}+\upsilon ){\textbf{P}}_{{\textbf{h}}},\\ \,_{0}^{CF}{\textbf{D}}_{{\textbf{t}}}^{\chi }{{\textbf{Q}}_{{\textbf{r}}}}=\alpha _{2}{\textbf{P}}_{{\textbf{r}}}-(\upsilon +\vartheta _{2}){\textbf{Q}}_{{\textbf{r}}},\\ \,_{0}^{CF}{\textbf{D}}_{{\textbf{t}}}^{\chi }{{\textbf{G}}_{{\textbf{s}}}}=\beta _{1}{\textbf{Q}}_{{\textbf{h}}{\textbf{a}}}+\beta _{2}{\textbf{Q}}_{{\textbf{h}}{\textbf{s}}}+\beta _{3}{\textbf{Q}}_{{\textbf{r}}}-(\xi _{2}+\xi _{3}){\textbf{G}}_{{\textbf{s}}},\\ \,_{0}^{CF}{\textbf{D}}_{{\textbf{t}}}^{\chi }{{\textbf{G}}_{{\textbf{a}}}}=\xi _{3}{\textbf{G}}_{{\textbf{s}}}-\xi _{2}{\textbf{G}}_{{\textbf{a}}}, \\ \end{array}\right. } \end{aligned}$$

85 $$\begin{aligned} {\left\{ \begin{array}{ll} d{\textbf{X}}_{{\textbf{h}}}=\big [\Lambda _{1}-\Upsilon _{{\textbf{h}}}{\textbf{X}}_{{\textbf{h}}}-\vartheta _{1}{\textbf{X}}_{{\textbf{h}}}\big ]d{\textbf{t}}+\sigma _{1}{\textbf{X}}_{{\textbf{h}}}d{\mathcal {B}}_{1}({\textbf{t}}),\\ d{\textbf{P}}_{{\textbf{h}}}=\big [\Upsilon _{{\textbf{h}}}{\textbf{X}}_{{\textbf{h}}}-(\alpha _{1}+\vartheta _{1}){\textbf{P}}_{{\textbf{h}}}\big ]d{\textbf{t}}+\sigma _{2}{\textbf{P}}_{{\textbf{h}}}d{\mathcal {B}}_{2}({\textbf{t}}),\\ d{\textbf{Q}}_{{\textbf{h}}{\textbf{a}}}=\big [\mu \alpha _{1}{\textbf{P}}_{{\textbf{h}}}-(\phi _{1}+\vartheta _{1}){\textbf{Q}}_{{\textbf{h}}{\textbf{a}}}\big ]d{\textbf{t}}+\sigma _{3}{\textbf{Q}}_{{\textbf{h}}{\textbf{a}}}d{\mathcal {B}}_{3}({\textbf{t}}),\\ d{\textbf{Q}}_{{\textbf{h}}{\textbf{s}}}=\big [(1-\mu )\alpha _{1}{\textbf{P}}_{{\textbf{h}}}-(\delta +\phi _{2}+\vartheta _{1}){\textbf{Q}}_{{\textbf{h}}{\textbf{s}}}\big ]d{\textbf{t}}+\sigma _{4}{\textbf{Q}}_{{\textbf{h}}{\textbf{s}}}d{\mathcal {B}}_{4}({\textbf{t}}),\\ d{\textbf{R}}_{{\textbf{h}}}=\big [\phi _{1}{\textbf{P}}_{{\textbf{h}}}+\phi _{2}{\textbf{Q}}_{{\textbf{h}}{\textbf{s}}}-\vartheta _{1}{\textbf{R}}_{{\textbf{h}}}\big ]d{\textbf{t}}+\sigma _{5}{\textbf{R}}_{{\textbf{h}}}d{\mathcal {B}}_{5}({\textbf{t}}),~~~~~~{\textbf{t}}\in [{\mathbb {T}}_{2},{\mathbb {T}}],\\ d{\textbf{X}}_{{\textbf{r}}}=\big [\Lambda _{2}-\Upsilon _{{\textbf{r}}}{\textbf{X}}_{{\textbf{r}}}-(\vartheta _{2}+\upsilon ){\textbf{R}}_{{\textbf{h}}}\big ]d{\textbf{t}}+\sigma _{6}{\textbf{X}}_{{\textbf{r}}}d{\mathcal {B}}_{6}({\textbf{t}}),\\ d{\textbf{P}}_{{\textbf{r}}}=\big [\Upsilon _{{\textbf{r}}}{\textbf{X}}_{{\textbf{r}}}-(\alpha _{2}+\vartheta _{2}+\upsilon ){\textbf{P}}_{{\textbf{h}}}\big ]d{\textbf{t}}+\sigma _{7}{\textbf{P}}_{{\textbf{r}}}d{\mathcal {B}}_{7}({\textbf{t}}),\\ d{\textbf{Q}}_{{\textbf{r}}}=\big [\alpha _{2}{\textbf{P}}_{{\textbf{r}}}-(\upsilon +\vartheta _{2}){\textbf{Q}}_{{\textbf{r}}}\big ]d{\textbf{t}}+\sigma _{8}{\textbf{Q}}_{{\textbf{r}}}d{\mathcal {B}}_{8}({\textbf{t}}),\\ d{\textbf{G}}_{{\textbf{s}}}=\big [\beta _{1}{\textbf{Q}}_{{\textbf{h}}{\textbf{a}}}+\beta _{2}{\textbf{Q}}_{{\textbf{h}}{\textbf{s}}}+\beta _{3}{\textbf{Q}}_{{\textbf{r}}}-(\xi _{2}+\xi _{3}){\textbf{G}}_{{\textbf{s}}}\big ]d{\textbf{t}}+\sigma _{9}{\textbf{G}}_{{\textbf{s}}}d{\mathcal {B}}_{9}({\textbf{t}}),\\ d{\textbf{G}}_{{\textbf{a}}}=\big [\xi _{3}{\textbf{G}}_{{\textbf{s}}}-\xi _{2}{\textbf{G}}_{{\textbf{a}}}\big ]d{\textbf{t}}+\sigma _{10}{\textbf{G}}_{{\textbf{a}}}d{\mathcal {B}}_{10}({\textbf{t}}),\end{array}\right. } \end{aligned}$$

Now, we use the method outlined in^37^ to analyze the piecewise configuration (83–85) in the perspective of Atangana-Baleanu-Caputo derivative. We immediately begin the methodology by doing the following:

$$\begin{aligned} {\left\{ \begin{array}{ll} \frac{d\aleph _{{\textbf{k}}}({\textbf{t}})}{d{\textbf{t}}}=\Omega ({\textbf{t}},\aleph _{{\textbf{k}}}).~\aleph _{{\textbf{k}}}(0)=\aleph _{{\textbf{k}},0},~{\textbf{k}}=1,2,...,{\mathfrak {n}}~if~{\textbf{t}}\in [0,{\mathbb {T}}_{1}],\\ \,_{{\mathbb {T}}_{1}}^{ABC}{\textbf{D}}_{{\textbf{t}}}^{\chi }\aleph _{{\textbf{k}}}({\textbf{t}})=\Omega ({\textbf{t}},\aleph _{{\textbf{k}}}),~\aleph _{{\textbf{k}}}({\mathbb {T}}_{1})=\aleph _{{\textbf{k}},1},~if~{\textbf{t}}\in [{\mathbb {T}}_{1},{\mathbb {T}}_{2}],\\ d\aleph _{{\textbf{k}}}({\textbf{t}})=\Omega ({\textbf{t}},\aleph _{{\textbf{k}}})d{\textbf{t}}+\wp _{{\textbf{k}}}\aleph _{{\textbf{k}}}d{\mathcal {B}}_{{\textbf{k}}}({\textbf{t}}),~\aleph _{{\textbf{k}}}({\mathbb {T}}_{2})=\aleph _{{\textbf{k}},2},~if~{\textbf{t}}\in [{\mathbb {T}}_{2},{\mathbb {T}}]. \end{array}\right. } \end{aligned}$$

**Figure 3:**
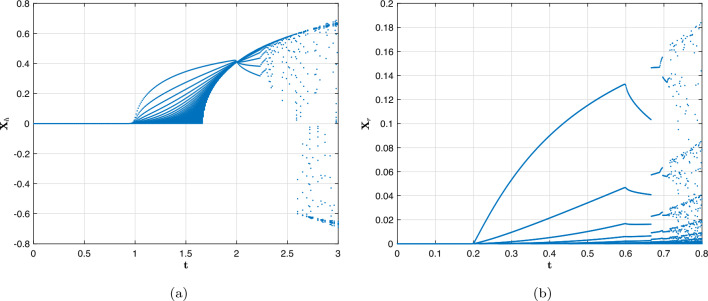
Bifurcation plots with respect to Theorem 2.2 with assumptions (i) and (ii).

Simple computation yields

$$\begin{aligned} \aleph _{{\textbf{k}}}^{{\textbf{v}}}={\left\{ \begin{array}{ll}\aleph _{{\textbf{k}}}(0)+\sum \limits _{{\textbf{m}}=2}^{{\textbf{v}}}\Big \{\frac{23}{12}\Omega ({{\textbf{t}}}_{{\textbf{m}}},\aleph ^{{\textbf{m}}})\Delta {\textbf{t}}-\frac{4}{3}\Omega ({{\textbf{t}}}_{{\textbf{m}}-1},\aleph ^{{\textbf{m}}-1})\Delta {\textbf{t}}+\frac{5}{12}\Omega ({{\textbf{t}}}_{{\textbf{m}}-2},\aleph ^{{\textbf{m}}-2})\Delta {\textbf{t}}\Big \},~~{\textbf{t}}\in [0,\mathbb {T_{1}}].\\ \aleph _{{\textbf{k}}}({\mathbb {T}}_{1})+\frac{\chi }{ABC(\chi )}\Omega ({{\textbf{t}}}_{{\mathfrak {n}}},\aleph ^{{\mathfrak {n}}})+\frac{\chi (\Delta {\textbf{t}})^{\chi -1}}{ABC(\chi )\Gamma (\chi +1)}\sum \limits _{{\textbf{m}}=2}^{{\textbf{v}}}\Omega ({{\textbf{t}}}_{{\textbf{m}}-2},\aleph ^{{\textbf{m}}-2})\Psi _{1}\\ \quad + \frac{\chi (\Delta {\textbf{t}})^{\chi -1}}{ABC(\chi )\Gamma (\chi +2)}\sum \limits _{{\textbf{m}}=2}^{{\textbf{v}}}\Big \{\Omega ({{\textbf{t}}}_{{\textbf{m}}-1},\aleph ^{{\textbf{m}}-1})-\Omega ({{\textbf{t}}}_{{\textbf{m}}-2},\aleph ^{{\textbf{m}}-2})\Big \}\Psi _{2}\\ \quad +\frac{\chi (\Delta {\textbf{t}})^{\chi -1}}{2ABC(\chi )\Gamma (\chi +3)}\sum \limits _{{\textbf{m}}=2}^{{\textbf{v}}}\Big \{\Omega ({{\textbf{t}}}_{{\textbf{m}}},\aleph ^{{\textbf{m}}})-2\Omega ({{\textbf{t}}}_{{\textbf{m}}-1},\aleph ^{{\textbf{m}}-1})+\Omega ({{\textbf{t}}}_{{\textbf{m}}-2},\aleph ^{{\textbf{m}}-2})\Big \}\Psi _{3},~~{\textbf{t}}\in [{\mathbb {T}}_{1},{\mathbb {T}}_{2}],\\ \aleph _{{\textbf{k}}}({\mathbb {T}}_{2})+\sum \limits _{{\textbf{m}}={\textbf{v}}+3}^{{\mathfrak {n}}}\Big \{\frac{5}{12}\Omega ({{\textbf{t}}}_{{\textbf{m}}-2},\aleph ^{{\textbf{m}}-2})\Delta {\textbf{t}}-\frac{4}{3}\Omega ({{\textbf{t}}}_{{\textbf{m}}-1},\aleph ^{{\textbf{m}}-1})\Delta {\textbf{t}}+\frac{23}{12}\Omega ({{\textbf{t}}}_{{\textbf{m}}},\aleph ^{{\textbf{m}}})\Delta {\textbf{t}}\Big \}\\ \quad +\sum \limits _{{\textbf{m}}={\textbf{v}}+3}^{{\mathfrak {n}}}\Big \{\frac{5}{12}\big ({\mathcal {B}}({{\textbf{t}}}_{{\textbf{m}}-1})-{\mathcal {B}}({{\textbf{t}}}_{{\textbf{m}}-2})\big )\wp \aleph ^{{\textbf{m}}-2}- \frac{4}{3}\big ({\mathcal {B}}({{\textbf{t}}}_{{\textbf{m}}})-{\mathcal {B}}({{\textbf{t}}}_{{\textbf{m}}-1})\big )\wp \aleph ^{{\textbf{m}}-1}\\ \quad +\frac{23}{12}\big ({\mathcal {B}}({{\textbf{t}}}_{{\textbf{m}}+1})-{\mathcal {B}}({{\textbf{t}}}_{{\textbf{m}}})\big )\wp \aleph ^{{\textbf{m}}}\Big \},~~{\textbf{t}}\in [{\mathbb {T}}_{2},{\mathbb {T}}],\end{array}\right. } \end{aligned}$$

where $\Psi _{1},\Psi _{2}$ and $\Psi _{3}$ are stated before in (75–77) (Fig. 3).

## Results and discussion

**Figure 4:**
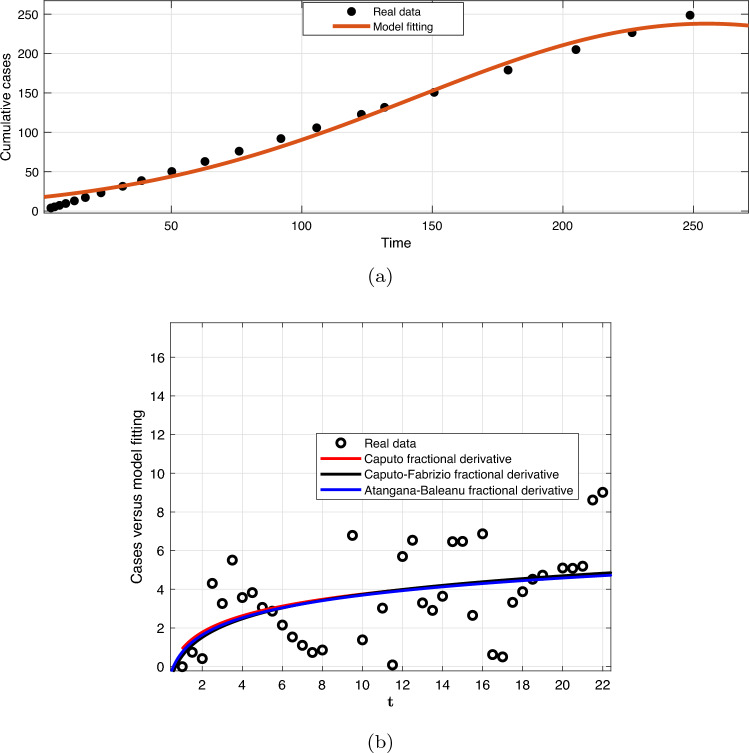
Lassa fever fitting outcomes considering the data obtained from Nigeria Centre for Disease Control^19^. (**a**) Cumulative cases (**b**) Weekly cases.

In this section, we will supply numerous practical instances along with numerical experiments to validate our above findings from analysis using the well-known piecewise fractional differential equations numerical technique provided by Seda and Atangana^37^. The associated parameter specifications of system (5) are presented in Table 2. We displayed the predictions compared to the data using real cases reported in Nigeria and acquired the outcomes visually in Figure 4. The cases are for the time frame of from November 28, 2022 to April 13, 2023 and are the most recently identified instances in the country. When ${\textbf{t}}=1$ and $\chi =1$ the data in Figure 4(a) has been fitted to the model.

### Parameter estimation

Currently, the computation of simulation parameters from indicated statistical data is attracting considerable interest among scholars and is regarded as a vital part of mathematical epidemiological research. We used the widely used nonlinear least squares technique to incorporate this component into the present work. The parameters were calculated using the technique mentioned above, and the framework was fitted to real Lassa fever cases identified in Nigeria for a selected time period. Particularly, the model’s parameters were estimated by using the total number of confirmed infected people and deaths between the period of November 28, 2022 to April 13, 2023, Nigeria. The Ordinary Least Square solution was utilized for reducing the error terms with the help of (86), and the related relative error is used in the goodness of fit,

86 $$\begin{aligned} \min \Big \{\frac{\sum _{i=1}^{n}(I_{i}-{\hat{I}}_{i})^{2}}{\sum _{i=1}^{n}I^{2}_{i}}\Big \}. \end{aligned}$$

Here, the notion $I_{i}$ is the reported cumulative infected cases and ${\hat{I}}_{i}$ is the cumulative infected cases obtained from simulating the model. The simulated values of cumulative infection are calculated by summing up the individuals, which moves from the infected compartment to the quarantined class each day. The infected population predicted by the proposed system (3). It is clear from the figure that the deterministic curves are in good agreement with the real data. All the parameters are estimated except $\zeta _{1}=0.167$, which is assumed. Estimated values of parameters are shown in Table 2.

Figure 3 illustrates the bifurcations with respect to susceptible humans and rodents, respectively.

Following that, we intend to concentrate our efforts by examining the following two scenarios. We intend to concentrate on the three resulting components:

(i)  If the criterion $\Re _{0}>1$ entails, there is a unique ergodic stationary distribution.

(ii) The effect of environmental noise on mechanisms (5) illness extermination.

(iii) The effect of transmission rate $\rho \in [0,1]$ on mechanism (5) illness dynamics.

### Experimental examples

In what follows, we illustrate two examples in order to support the mathematical outcomes provided in previous sections.

#### Example 5.1

**Figure 5:**
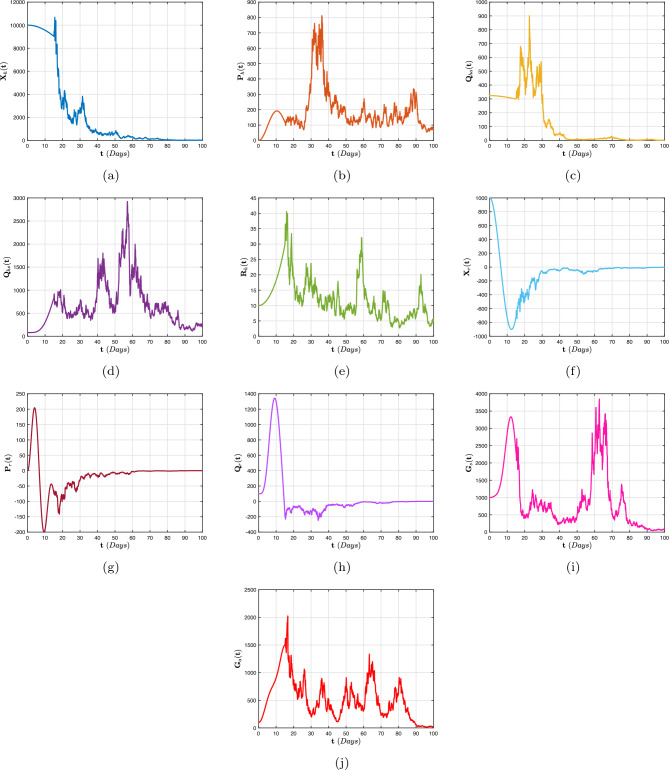
Convoluted two-dimensional view of the Lassa fever model (72–74) by means of a power-law kernel with $\chi =0.95$ and random perturbations.

Assume that the random perturbations $(\sigma _{1},\sigma _{2},...,\sigma _{10})=(0.001,0.001,...,0.001),$ we determined $\Re _{0}=3.1301>1$ and $\Re _{0}^{s}=13.001>1,$ which indicates that the illness will persist over time in a framework that is deterministic (3). Furthermore, we can deduce from Theorems 3.1 and 3.6 that model (5) confesses a global non-negative stationary outcomes on $\Re _{+}^{10}$, as shown in the Fig. 5(a,b,c,d,e,f,g,h,i and j). According to the biological significance of Fig. 5(i,j), the most efficient interaction given between ${\textbf{X}}_{{\textbf{h}}}$ and ${\textbf{Q}}_{{\textbf{r}}}$ causes the greatest harm in terms of pathological advancement when power law kernels have been employed. This is accompanied by a low transmission rate within ${\textbf{X}}_{{\textbf{h}}}$ and fewer transmissible infectious asymptomatic humans when fractional-order is assumed to be $\chi =0.95.$ By interacting with polluted atmospheres, dirty air and spreading indicative individuals, one can diminish the virus infection. We find that each means of dissemination contributes to the spread of Lassa fever, but certain are more important than others as well. It is clear that there is a huge disparity in the extent of dissemination of the pathways in ${\mathbb {N}}_{{\textbf{r}}}$ as well as infection individuals. This demonstrates that while certain routes prove more lethal than others, every process contributes to a distinctive approach.

#### Example 5.2

**Figure 6:**
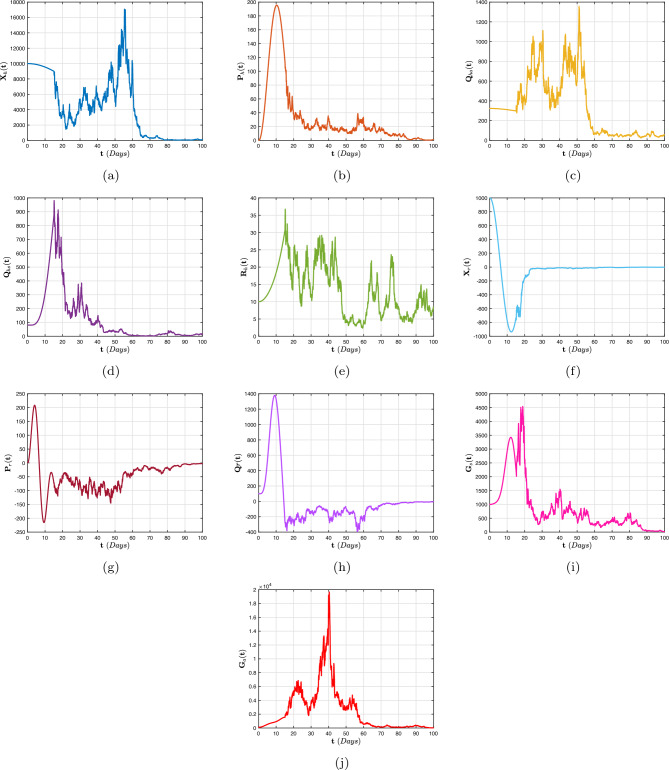
Convoluted two-dimensional view of the Lassa fever model (78–80) by means of a exponential decay kernel with $\chi =0.95$ and random perturbations.

We surmise the random perturbations $(\sigma _{1},...,\sigma _{10})=(0.001,...,0.001),$ a simple computation yields $\Re _{0}^{s}=0.05789<1$ and according to Theorem 3.4 the outcomes of system (5) must satisfies

$$\begin{aligned}{} & {} \lim \limits _{{\textbf{t}}\mapsto \infty }\sup \frac{\log {\textbf{Q}}_{{\textbf{h}}{\textbf{a}}}}{{\textbf{t}}}\le \big (\phi _{1}+\vartheta _{1}+\frac{\sigma _{3}^{2}}{2}\big )\big [\Re _{0}^{s}-1\big ]<0~~a.s,\nonumber \\ {}{} & {} \lim \limits _{{\textbf{t}}\mapsto \infty }\sup \frac{\log {\textbf{Q}}_{{\textbf{h}}{\textbf{s}}}}{{\textbf{t}}}\le \big (\delta +\phi _{2}+\vartheta _{1}+\frac{\sigma _{4}^{2}}{2}\big )\big [\Re _{0}^{s}-1\big ]<0~~a.s, \end{aligned}$$

and

$$\begin{aligned} \lim \limits _{{\textbf{t}}\mapsto \infty }\sup \frac{\log {\textbf{Q}}_{{\textbf{r}}}}{{\textbf{t}}}\le \big (\upsilon +\vartheta _{2}+\frac{\sigma _{8}^{2}}{2}\big )\big [\Re _{0}^{s}-1\big ]<0~~a.s. \end{aligned}$$

shows that model (5) has a stable endemic equilibrium $\Re _{+}^{10}$. On the contrary, we can derive the condition systemic from Theorem (3.4) will become disappearing with a unit probability. Figure 6(a,b,c,d,e,f,g,h,i, and j) indicate the appropriate numerical modeling of the solution ${\tilde{\Theta }}\in \Re _{+}^{10}$ to model (5) with the low random intensities and piecewise fractional differential equations scheme.

Taking into account the fractional calculus and biophysical approach, Fig. 6(i,j) shows that combining two dissemination processes improves the prevalence of diseases compared to an individual process when an exponential decay type fractional derivative has been applied with a fractional-order $\chi =0.95$. We additionally discover that specific blends are more lethal than other people. Any interaction involving the efficient interaction rate among ${\textbf{X}}_{{\textbf{h}}}$ and ${\textbf{Q}}_{{\textbf{r}}}$ results in a spike of transmission, which is subsequently accompanied by any pairing via the successful interaction rate between ${\textbf{X}}_{{\textbf{h}}}$ and ${\textbf{Q}}_{{\textbf{h}}{\textbf{a}}}$ and then additional processes.

#### Example 5.3

**Figure 7:**
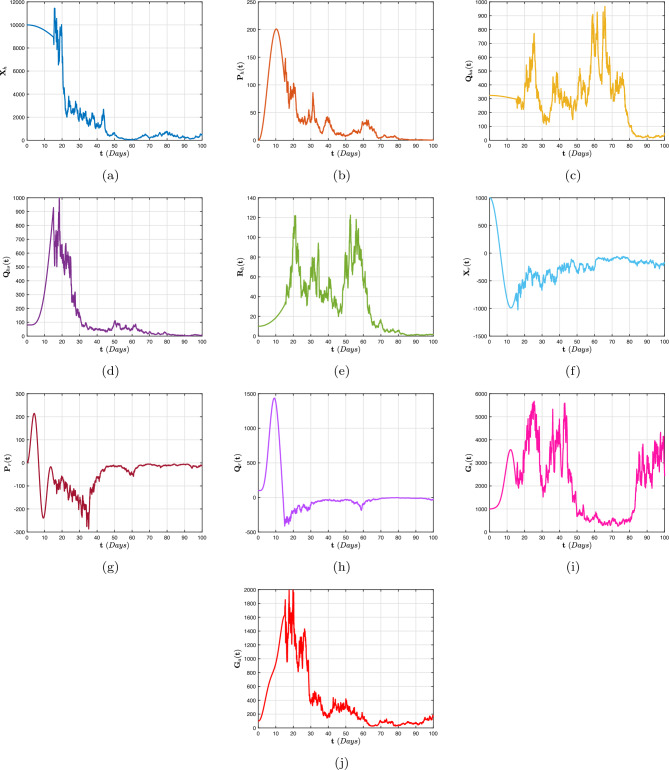
Convoluted two-dimensional view of the Lassa fever model (83–85) by means of a generalized Mittag-Leffler kernel with $\chi =0.95$ and random perturbations.

Now fixing the random perturbations $(\sigma _{1},...,\sigma _{10})=(0.001,...,0.001),$ Fig. 7(a,b,c,d,e,f,g,h,i and j),

depicts the accompanying variability developments of $\Re _{0}$ and $\Re _{0}^{p}$ with varying $\rho \in (0,1)\times 10^{-5}$ are presented. $\Re _{0}^{p}>1$ is obviously obtained when $\rho >0.501\times 10^{-6}$ and $\Re _{0}>0.25$ when $\rho <0.07536\times 10^{-7}$ is obtained. Utilizing Theorems 233.6, it is shown that the infection model (5) will be widespread when $\rho >0.501\times 10^{-6}$, but vanish when $\rho <0.07536\times 10^{-7}$. We specifically examine the subsequent three cases of: **(i)** $\rho =5\times 10^{-5},$ **(ii)** $\rho =5\times 10^{-6}$ and **(iii)** $\rho =5\times 10^{-7}.$ Fig. 7(i,j) depict the appropriate computational models for ${\textbf{X}}_{{\textbf{h}}}$ and ${\textbf{P}}_{{\textbf{h}}}$ within the framework (5). As the duration at which it propagates declines, the number of ${\textbf{X}}_{{\textbf{h}}}$ increases, the infectious disease ${\textbf{Q}}_{{\textbf{h}}{\textbf{a}}},~{\textbf{Q}}_{{\textbf{h}}{\textbf{s}}}$ and ${\textbf{Q}}_{{\textbf{r}}}$ are Fig. 7 depicts the appropriate computational models for individuals ${\textbf{X}}_{{\textbf{h}}}$ and ${\textbf{P}}_{{\textbf{h}}}$ within the framework (5). As the duration at which it propagates declines, the number of susceptible individuals increases, and the infectious disease is wiped out when low perturbations and a generalized Mittag-Leffler kernel are utilized with the scheme of piecewise fractional differential equations.

Furthermore, in Fig. 7, each of the dissemination pathways is shown in addition to the prevailing point dissemination route with the ABC fractional derivative operator. The distinction within both charts takes into consideration the impact of additional processes as well as the efficient interaction rate within ${\textbf{X}}_{H_{}1}$ and ${\textbf{Q}}_{{\textbf{r}}}$. This demonstrates that, while the efficient interaction rate in ${\textbf{X}}_{{\textbf{h}}}$ and ${\textbf{Q}}_{{\textbf{r}}}$ is prevailing, different processes cannot be overlooked since, if they function together, they result in a supplementary rise in the impact of Lassa fever periodically. The design is additionally essential to point out that horizontal transfers between ${\textbf{X}}_{{\textbf{r}}}$ and ${\textbf{Q}}_{{\textbf{r}}}$ perform a noteworthy function in boosting the prevalence of infection along with interaction rates in ${\textbf{X}}_{{\textbf{r}}}$ and affected surroundings.

According to our fractional and stochastic design exercises, every possible transmission process has an effect on the advancement of Lassa fever. Interestingly, certain means of transmission produce substantially higher amounts than individuals. Prevention strategies should focus on lowering collision ranks within ${\textbf{X}}_{{\textbf{h}}}$ and ${\textbf{Q}}_{{\textbf{r}}}$ (particularly in locations that have elevated rodent usage) and interaction ranks between ${\textbf{X}}_{{\textbf{h}}}$ and ${\textbf{Q}}_{{\textbf{h}}{\textbf{a}}}$. While confronted by ${\textbf{X}}_{{\textbf{h}}}$ and ${\textbf{Q}}_{{\textbf{h}}{\textbf{a}}}$, a major obstacle emerges as these infections are hard to recognize by means of indicators. This necessitates the use of detection strategies, which include widespread sampling in native regions, immunizations and advanced fractional calculus operators.

### Bifurcation analysis

**Figure 8:**
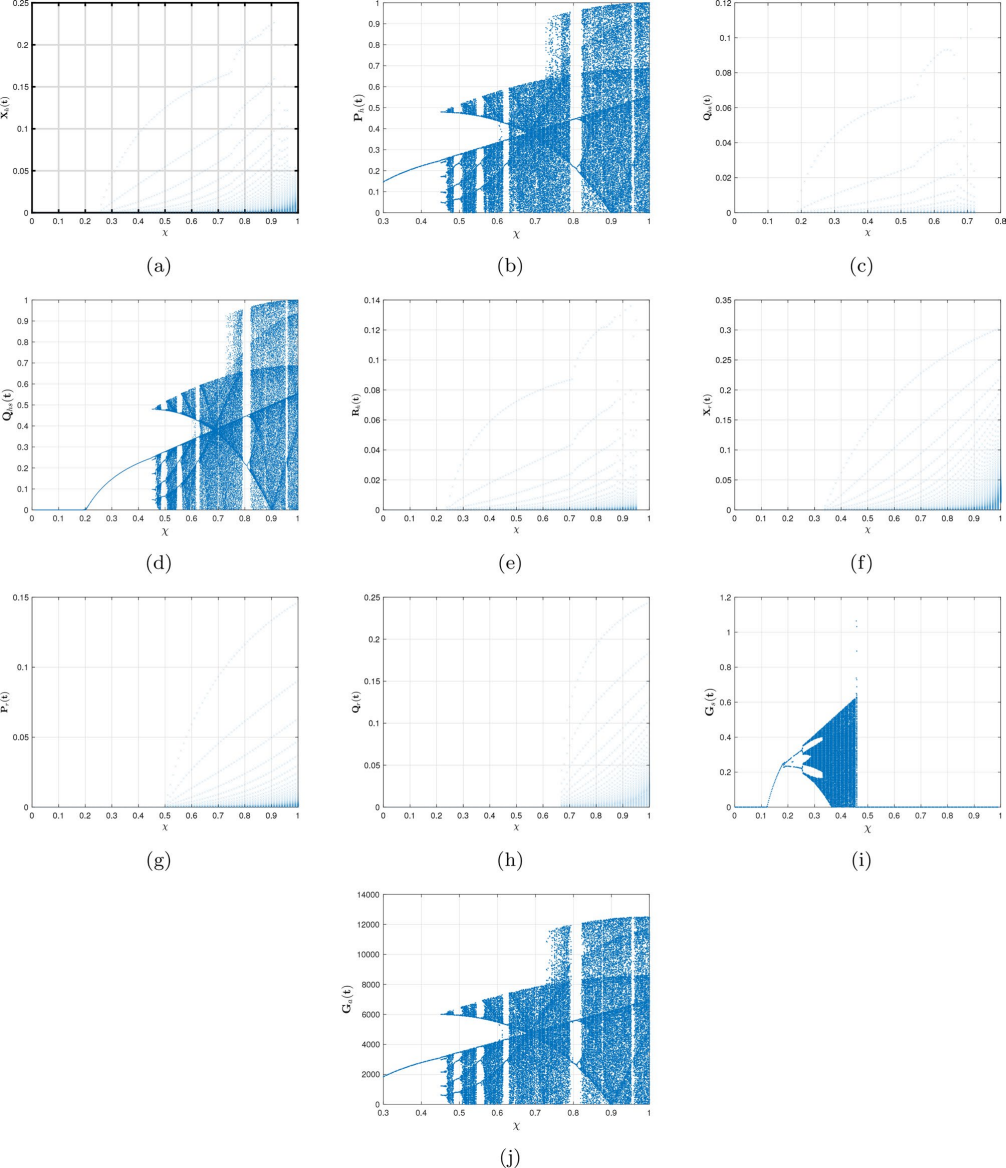
Bifurcation plots for the Caputo fractional derivative operator for (73) when $\chi =0.95$.

Bifurcations play a vital role in dynamics research for fractional-order systems. Therefore, in this section, bifurcation analysis is conducted to study the rich dynamical behavior of the fractional-order system (7) in the two cases of commensurate-order and incommensurate-order, respectively. Using the parametric values in Table 2, the roots of the (11) are −0.6213, −1.3423, −0.1236, 0.1122, −0.2345, −1.2345, −0.3245, −0.5677, 0.5624$\pm 0.3425\iota .$ Thus we obtain $\Re _{c}(0.1122)\ne 0.$ The bifurcation diagram is shown in Fig. 8(a) through Fig. 8(j). For $\Upsilon _{{\textbf{h}}}^{*}=32.45$ and $\Upsilon _{{\textbf{r}}}^{*}=0.3456$ and ${\mathcal {E}}_{1}=\big ({\textbf{X}}_{{\textbf{h}}}^{*},{\textbf{P}}_{{\textbf{h}}}^{*},{\textbf{Q}}_{{\textbf{h}}{\textbf{a}}}^{*},{\textbf{Q}}_{{\textbf{h}}{\textbf{s}}}^{*},{\textbf{R}}_{{\textbf{h}}}^{*},{\textbf{X}}_{{\textbf{r}}}^{*},{\textbf{P}}_{{\textbf{r}}}^{*},{\textbf{Q}}_{{\textbf{r}}}^{*},{\textbf{G}}_{{\textbf{s}}}^{*},{\textbf{G}}_{{\textbf{a}}}^{*}\big )$, we obtain ${\mathcal {E}}_{1}=\big (7.8345,295.6534,120.2340,56.7823,35.6547,23.4501,19.2301,13.456,11.9002,10.2090\big ).$ Now, ${\mathcal {E}}_{1}$ is locally asymptotically stable when $\chi \in (0,1]$ confirming our theoretical results in Theorem 2.3 (assertion  (**aiii**)). The system (7) produces a bifurcation when $\chi =0.95.$

## Conclusion

In this manuscript, we construct and verify a deterministic-stochastic scheme for analyzing the Lassa fever infection, including several modes of transmission, to address their effect on contamination growth in a community. To begin, we illustrate that the framework (5) has a single global positive findings for any particular initial conditions. Following that, we employ the stochastic Lyapunov candidate technique to identify sufficient requirements for determining the presence and distinctive characteristics of an ergodic stationary distribution, known as a distribution of chances exhibiting certain inflexible features. In view of the distinct features of $\Re _{0}^{s},$ we succeeded in demonstrating the way including multiple dissemination processes influences illness incidence. We employed stochastic tools to determine the extinction. We obtained mathematical formulas from our investigation that demonstrate the situations that dictate whether the illness can endure or can be regulated in the framework, as well as the manner in which the response of setting modifications results in system operation improvements. According to our system modeling, every single propagation route has an effect on the advancement of Lassa fever. But certain pathways of transmission are contributing substantially greater amounts than others as well. Our findings indicate that measures in these fields ought to be avoided and overlooked when developing health strategies. Additional research can be conducted:

(i) an amalgam of various methods for propagation that takes into account the unpredictable nature of disease,
(ii) Effectively sanitary conditions, approaches to intervention, and multifaceted prevention initiatives that incorporate such several dissemination processes can aid in the reduction of illness incidence in the overall health sector,
(iii) Employing the technique of cost-effective assessment, optimize the expense of multiple intervention strategies to ensure people in regions struggling with impoverishment challenges can be adequately aided.

Being able to get to actual information and involving Lévy noise, Poisson noise and telegraph noise may additionally enhance the model’s anticipatory capability. Vertical propagation of Lassa fever in rodents may additionally be included in future research. Other approaches, such as system scaling, may be employed to assist in the evaluation when the settings are without dimensions and convey proportions of tangible repercussions instead of capacities of specific implications.

## Data Availability

The datasets used and/or analyzed during the current study available from the corresponding author on reasonable request.
