# Author Correction: Novel insights for a nonlinear deterministic-stochastic class of fractional-order Lassa fever model with varying kernels

**DOI:** 10.1038/s41598-023-44496-7

**Published:** 2023-10-12

**Authors:** Saima Rashid, Shazia Karim, Ali Akgül, Abdul Bariq, S. K. Elagan

**Affiliations:** 1 Department of Mathematics, Government College University, Faisalabad, 38000 Pakistan; 2 Department of Computer Science and Mathematics, Lebanese American University, Beirut, Lebanon; 3 Department of Basic Sciences, UET Lahore, , FSD Campus, Lahore, 54800 Pakistan; 4 Department of Mathematics, Art and Science Faculty, Siirt University, 56100 Siirt, Turkey; 5 Department of Mathematics, Mathematics Research Center, Near East University, Near East Boulevard, 99138 Nicosia/Mersin 10, Turkey; 6 Department of Mathematics, Laghman University, Mehtarlam City 2701, Laghman, Afghanistan; 7 Department of Mathematics and Statistics, Taif University, Taif, Saudi Arabia

Correction to: *Scientific Reports* 10.1038/s41598-023-42106-0, published online 15 September 2023

The original version of this Article contained an error. In the original version of this article, the Acknowledgements section was missing. The Acknowledgements section now reads:

“The researchers would like to acknowledge the Deanship of Scientific Research at Taif University for funding this work.”

The original Article has been corrected.

